# Imbalanced Regulation of Fungal Nutrient Transports According to Phosphate Availability in a Symbiocosm Formed by Poplar, Sorghum, and *Rhizophagus irregularis*

**DOI:** 10.3389/fpls.2019.01617

**Published:** 2019-12-12

**Authors:** Silvia Calabrese, Loic Cusant, Alexis Sarazin, Annette Niehl, Alexander Erban, Daphnée Brulé, Ghislaine Recorbet, Daniel Wipf, Christophe Roux, Joachim Kopka, Thomas Boller, Pierre-Emmanuel Courty

**Affiliations:** ^1^Department of Environmental Sciences, Botany, Zurich-Basel Plant Science Center, University of Basel, Basel, Switzerland; ^2^Laboratoire de Recherche en Sciences Végétales, Université de Toulouse, UPS, CNRS, Castanet-Tolosan, France; ^3^Department of Biology at the Swiss Federal Institute of Technology Zurich, Zurich, Switzerland; ^4^Max Planck Institute of Molecular Plant Physiology, Potsdam-Golm, Germany; ^5^Agroécologie, AgroSup Dijon, CNRS, INRAE, Univ. Bourgogne, Univ. Bourgogne Franche-Comté, Dijon, France

**Keywords:** arbuscular mycorrhizal fungi, symbiocosm, extraradical mycelium, intraradical mycelium, phosphorus, ammonium, carbohydrates transporters, lipid metabolism

## Abstract

In arbuscular mycorrhizal (AM) symbiosis, key components of nutrient uptake and exchange are specialized transporters that facilitate nutrient transport across membranes. As phosphate is a nutrient and a regulator of nutrient exchanges, we investigated the effect of P availability to extraradical mycelium (ERM) on both plant and fungus transcriptomes and metabolomes in a symbiocosm system. By perturbing nutrient exchanges under the control of P, our objectives were to identify new fungal genes involved in nutrient transports, and to characterize in which extent the fungus differentially modulates its metabolism when interacting with two different plant species. We performed transportome analysis on the ERM and intraradical mycelium of the AM fungus *Rhizophagus irregularis* associated to *Populus trichocarpa* and *Sorghum bicolor* under high and low P availability in ERM, using quantitative RT-PCR and Illumina mRNA-sequencing. We observed that mycorrhizal symbiosis induces expression of specific phosphate and ammonium transporters in both plants. Furthermore, we identified new AM-inducible transporters and showed that a subset of phosphate transporters is regulated independently of symbiotic nutrient exchange. mRNA-Sequencing revealed that the fungal transportome was not similarly regulated in the two host plant species according to P availability. Mirroring this effect, many plant carbohydrate transporters were down-regulated in *P. trichocarpa* mycorrhizal root tissue. Metabolome analysis revealed further that AM root colonization led to a modification of root primary metabolism under low and high P availability and to a decrease of primary metabolite pools in general. Moreover, the down regulation of the sucrose transporters suggests that the plant limits carbohydrate long distance transport (i.e. from shoot to the mycorrhizal roots). By simultaneous uptake/reuptake of nutrients from the apoplast at the biotrophic interface, plant and fungus are both able to control reciprocal nutrient fluxes.

## Highlight

Gene expression analysis in arbuscular mycorrhizal symbiosis upon phosphate variations reveals imbalanced fungal gene regulation between perennial/C3 and annual/C4 host, highlights new mycorrhiza-inducible transporters and suggests active fungal carbohydrate uptake.

## Introduction

Phosphorus (P), and nitrogen (N), are among the most essential nutrients for plants. As P is present in many biological compounds and is involved in many key metabolic processes, it can make up to 0.2% of the dry weight of a plant (Schachtman et al., 1998). In living plants, the cellular inorganic phosphate (Pi) concentration ranges between 1 and 10 mM whereas the soil water Pi concentration, is about 10,000-times less (Rausch and Bucher, 2002; Ai et al., 2009; Branscheid et al., 2010): Due to its negative charge and its low mobility, Pi is rapidly sequestered by cations and organic substances in the soil (e.g. clay) and therefore only barely accessible to plants (Poirier and Bucher, 2002; Aung et al., 2006; Chiou et al., 2006; Javot et al., 2007a; Tatry et al., 2009). To prevent mineral nutrient deficiency, and P deficiency in particular, a majority of land plants form symbioses with the so called arbuscular mycorrhizal (AM) fungi. In the AM symbiosis the AM fungus provides macro and micro nutrients to the plants and in return receives carbohydrates from the host plant (Smith and Read, 2008). AM fungi have a broad host range: each fungal species, each genet can colonize several plant individuals, also from different species, connected each other and forming a so called common mycorrhizal network (Walder et al., 2012; Fellbaum et al., 2014). The extraradical mycelium (ERM) of the AM fungi engaged in such a network is able to acquire nutrients that are out of reach or not accessible to the plant partners. For a given plant, this makes the mycorrhizal uptake pathway often more effective than the direct uptake pathway (Wipf et al., 2019). The nutrients taken up by the ERM are transported to the hyphal network inside the host root *via* the intraradical mycelium (IRM), which forms highly branched tree-like structures (arbuscules) inside the root cortical cells. The arbuscules are still surrounded by the plant cell-derived periarbuscular membrane and the inter-membrane interstice, the periarbuscular space. Within this structure, the mineral nutrients acquired by the AM fungus are exported to the periarbuscular space, and taken up by plant nutrient transporters of the periarbuscular membrane (Smith and Smith, 2011).

With respect to Pi, the extent to which plants cover their Pi-demand through the AM fungus ranges from only a small percentage to 100% (Paszkowski, 2006; Javot et al., 2007a). The Pi taken up by the ERM is incorporated into poly-P, transported to the arbuscules, hydrolyzed and translocated to the periarbuscular space (Ezawa et al., 2002; Javot et al., 2007a; Kikuchi et al., 2016). Essential key players in this process are transporters and permeases that facilitate uptake and transport of nutrients across membranes. The expression of transporters is regulated by nutrient availability. In this way, a steady and efficient translocation of nutrients adapted to given environmental conditions can be guaranteed (Smith and Smith, 2011; Courty et al., 2015; Garcia et al., 2016; Wipf et al., 2019).

In AM fungi, only few phosphate transporters (PT) were characterized so far: one in *Funneliformis mosseae* (GmosPT, Benedetto et al., 2005) and *Glomus versiforme* (GvPT, Harrison and Buuren 1995), and seven in *Rhizophagus irregularis* (formerly *Glomus intraradices*, RiPT1-RiPT7; Maldonado-Mendoza et al., 2001; Walder et al., 2016). Expression of the three high affinity transporters, *RiPT1*, *GvPT*, and *GmosPT*, is dependent on external Pi concentrations in the ERM (Harrison and Buuren, 1995; Maldonado-Mendoza et al., 2001; Benedetto et al., 2005). Reduced expression of *GmosPT* in the IRM compared to the ERM suggested a concentration-dependent regulation of PTs in the symbiotic root tissue.

In plants, the family of PTs can be divided into three subfamilies. Subfamily I transporters (Pht1) are membrane bound H^+^/P symporters driven by an H^+^ gradient. They are a subgroup of the major facilitator superfamily to which most of the PTs known to date belong (Pao et al., 1998). Subfamily II members are located in the plastids and function as antiporters (Poirier and Bucher, 2002; Rausch and Bucher, 2002; Javot et al., 2007b); members of the subfamily III are located in the mitochondrial inner membrane and are predicted to function as H^+^/P symporters or as P/OH^-^ antiporters (Takabatake et al., 1999; Javot et al., 2007b). In mycorrhizal plants, some Pht1 PT are specifically induced during symbiotic interaction. The first mycorrhiza-inducible PT was identified in *Solanum tuberosum* (StPT3) and was localized in arbusculated root sections (Rausch et al., 2001). Then, more mycorrhiza-inducible transporters were identified in several other plants (Harrison et al., 2002; Glassop et al., 2005; Nagy et al., 2005; Loth-Pereda et al., 2011). StPT3 and MtPT4 from *Medicago truncatula* were present in the periarbuscular membrane only. Furthermore, it was demonstrated that MtPT4-deficient plants accumulated Pi as poly-P in the arbuscules, which resulted in a premature collapse of the arbuscules and in inefficient symbiosis (Javot et al., 2007a; Breuillin-Sessoms et al., 2015).

For N, it was long assumed that AM symbiosis plays only a minor role in plant nutrition. In the soil, inorganic N is mostly present as nitrate or ammonium, both of which are readily mobile in the soil. Therefore, it was assumed that AM fungi take up N with the same efficiency as plants (Marschner and Dell, 1994; Hodge et al., 2010; Smith and Smith, 2011). But, it was shown that AM fungi can contribute up to 42% of the plant N demand (Frey and Schüepp, 1993; Mäder et al., 2000; Govindarajulu et al., 2005). In addition to the uptake of ammonium and nitrate, it was shown that AM fungi can take up N in the form of organic molecules (*i.e.* small peptides and amino acids) (Bago et al., 1996; Hawkins et al., 2000; Govindarajulu et al., 2005; Jin et al., 2005) and possibly also in the form of more complex organic compounds (Leigh et al., 2009; Hodge et al., 2010). However, in plants as well as in AM fungi, it was shown that ammonium is the preferred N source, as it can be directly incorporated into glutamine by the glutamine synthetase/glutamine oxoglutarate aminotransferase (GS/GOGAT) pathway whereas nitrate needs to be reduced to ammonium before N assimilation in the GS/GOGAT pathway (Villegas et al., 1996; Hawkins et al., 2000; Toussaint et al., 2004). In the ERM of AM fungi, the glutamine is further metabolized into amino acids such as arginine, alanine and asparagine for transport. Studies using ^15^N showed that arginine was the most common labelled amino acid in the ERM of AM fungi (Govindarajulu et al., 2005). Arginine is then transported from the ERM to the IRM where it is cleaved by arginases in the arbuscules. The released ammonium is transported to the periarbuscular space where it can be taken up by the plant ammonium transporters (AMT). So far, only six transporters have been identified and functionally characterized in Glomeromycotina, three in *R. irregularis* GintAMT1, GintAMT2 and GintAMT3 (López-Pedrosa et al., 2006; Pérez-Tienda et al., 2011; Calabrese et al., 2016), and three in *Geosiphon pyriformis* (GpAmt1, GpAmt2, GpAmt3; Ellerbeck et al., 2013).

In plants, the family of AMT can be divided into two subfamilies: subfamily I and subfamily II (reviewed in Courty et al., 2015). While members of the subfamily I were found to be mostly expressed in roots, members of the subfamily II were preferentially expressed in shoots (Couturier et al., 2007). Several mycorrhiza-inducible AMTs have been identified through gene expression analysis in several plant species (Gomez et al., 2009; Guether et al., 2009), including *PtAMT1.2* in poplar (Selle et al., 2005; Couturier et al., 2007) and *SbAMT3.1* and *SbAMT4* in sorghum (Koegel et al., 2013).

In return for the mineral nutrient, the AM fungi receives photosynthetates from the plants. In absence of gene encoding fatty acid synthase in their genome (Wewer et al., 2014; Tang et al., 2016), AM fungi are dependent to host fatty acids. Although the mechanism of transfer remains unknown, it was demonstrated that palmitic acid is transferred from plant roots to symbiotic mycelium, putatively esterified as mono-acyl-glycerol (Bravo et al., 2017; Jiang et al., 2017; Luginbuehl et al., 2017). Research on sugar transporter (SUT) expression in plants is not as consistent. Mycorrhization caused either increased or decreased expression of SUTs in root and shoots of the host plants (Ge et al., 2008; Boldt et al., 2011; Doidy et al., 2012a). A new class of SUT, the SWEETs, was identified. These transporters are located in the plasma membrane and have been shown to function as bidirectional sugar uniporters (Chen et al., 2010). Due to their involvement in rhizobial symbiosis it is assumed that they may also play a role in other biotrophic plant symbioses such as the AM symbiosis (Gamas et al., 1996; Doidy et al., 2012b).

In the AM fungus *R. irregularis*, four carbohydrate transporters have been identified (Helber et al., 2011). The gene encoding the monosaccharide transporter (MST) *RiMST2* was the most highly expressed transporter in symbiotic tissue, and it could be localized in the arbuscules and in the IRM. Within this line, An et al. (2019) recently showed that the expression of the gene encoding *M. truncatula SWEET1b* transporter is strongly enhanced in arbuscule-containing cells compared to roots and localizes to the peri-arbuscule membrane.

A comprehensive view of symbiosis under environmental and nutritional stress is important in times of climate change and resource shortening, as AM symbiosis is a key component of nutrient uptake and exchange through their transport systems. However, despite accumulating knowledge about transporter expression and metabolic activity in AM symbiosis, we still lack a precise understanding about their regulation and their importance for metabolism. Here, our objectives were to define the effects of P availability on two plants connected through a mycorrhizal network. Therefore, we analyzed the effects of mycorrhization and contrasting P nutrition on the transporter expression and metabolite accumulation in *Populus trichocarpa* (poplar) *and Sorghum bicolor* (sorghum) when colonized by the AM fungus *R. irregularis*. Our main focus was on the regulatory role of the mycorrhization and P nutrition on the expression of the Pht1 PTs in the plants and in the AM fungus. Further we assessed the effect of the applied conditions on AMTs and carbohydrate transporters. In the AM fungus *R. irregularis*, we determined expression of PTs, AMTs, MST, and fatty acid metabolism in the ERM and in the IRM of colonized poplar and sorghum roots. We identified new specific mycorrhiza-inducible PTs and AMTs in poplar and sorghum. Moreover, our data allowed us to gain further insight into symbiotic carbon exchange.

## Material and Methods

### Experimental Set-Up

Experiments were performed with *P. trichocarpa* cuttings (clone 10174, Orléans, France) and *S. bicolor* (L.) Moench, cv Pant-5. Sorghum seeds were kindly provided by Indian Grassland and Fodder Research Institute (Chaudhary Charan Singh Agriculture University of Hissar, Haryana, India) and Govind Ballabh Pant University of Agriculture and Technology (Pantanagar, Uttaranchal, India). Seeds were surface-sterilized in 2.5% KClO for 10 min, rinsed several times with sterile deionized water and soaked overnight in sterile deionized water. Seeds were germinated in the dark at 25°C for 3 days. Plants were inoculated with 1 ml liquid inoculum of *R. irregularis*, isolate BEG75 (Inoculum Plus, Dijon, France), in 0.01 M citrate buffer (pH 6) with about 110 spores/ml. The microcosms were set up in tripartite compartments (mycorrhizal treatment) or single compartments (non-mycorrhizal treatments). Compartments were filled with an autoclaved (120°C, 20 min) quartz sand (Alsace, Kaltenhouse, Trafor AG, Basel): zeolite (Symbion, Czech Republic) substrate (1:1, w:w). In the tripartite compartment system poplar cuttings were planted in the left compartment and sorghum seedlings in the right compartment. Both plants were inoculated with *R. irregularis* to create a common mycorrhizal network and to increase poplar root colonization (**Figure S1**). Compartments were separated by two 21 µm meshes and one 3 mm mesh, to allow the AM fungus to grow from one compartment to the other but to avoid plant roots protruding the neighboring compartment. As control, non-inoculated poplar and sorghum plantlets grew in single compartments receiving the Pi containing fertilizer treatments directly to their roots. Plants were fertilized once a week with 10 ml Hoagland solution without P, until all plants showed signs of P depletion, indicated by anthocyan accumulation. From the 22^nd^ week high-P (560 µM) or low-P (28 µM) containing Hoagland solution was applied to the first compartment for 9 weeks, to obtain ERM and to ensure that P was delivered *via* the mycorrhizal uptake pathway. Control plants received fertilizer treatment directly to their root systems.

### Harvest

The ERM was harvested by dispersing the substrate with tap water and fishing it from the surface using a 32 µM mesh. These steps were repeated several times. Afterwards the cleaned ERM samples were snap frozen in liquid N and stored at -80°C.

For RNA extractions, two leaves from the top of Poplar plants and two young leaves of Sorghum plants were snap frozen in liquid N and stored at -80°C. The rest of the shoots was harvested and dried in an oven at 55°C for 4 days for total P measurement.

Roots were removed from substrate under tap water and cut into ∼1 cm small pieces. Two subsamples of about 100 mg were immediately frozen in liquid N and stored at -80°C. One subsample of about 100 mg was taken for root colonization measurements. The remaining roots were placed in a paper bag and dried at 55°C for 3.5 days for determination of total P and N content.

### Colonization Measurements and P Extraction

Roots were immersed in 10% KOH and stored at 4°C overnight. The next day, the roots were rinsed with tap water and immersed in 2% HCl for 1 h at room temperature. Then, the roots were rinsed with tap water, immersed in 0.005% trypan blue (w:v in lactic-acid: glycerol: water, 1:1:1, v:v:v) and stored at 4°C o/n. The next day, the roots were rinsed and immersed in lactic-acid glycerol water (1:1:1, v:v:v) for destaining (Brundrett et al., 1984). Total colonization count was performed using the magnified intersection method (McGonigle et al., 1990). Differences between means of variables were assessed by t-test (p < 0.05), using Microsoft Excel 2010.

For determination of P concentration in the plants, dried root and shoot samples of six biological replicates were ground using a ball mill. Up to 500 mg were used for the modified P extraction method by Murphy and Riley (1962).

### RNA Extraction

Total RNA was extracted from six biological replicates per plant species and mycelium, respectively. Total RNA was extracted from lyophilized extraradical mycelia, root and leaf samples using the RNeasy Plant Mini Kit (Qiagen, Courtaboeuf, France). RNA extracts were DNase treated with the DNA-free™ Kit, DNase Treatment and Removal Reagents (AMBION^®^ by life technologies). Total RNA was quantified with the Qbit RNA BR Assay kit and purity was estimated using the Nanodrop (ND-1000, Witec, Switzerland).

### Reverse Transcription and qRT-PCR

cDNAs from three biological replicates were obtained using the iScript™ cDNA Synthesis Kit (BIO RAD Laboratories, Paolo Alto, CA, United States), using 200 ng of total RNA per reaction, using same RNA extracts as for mRNA sequencing. For quantification a two-step quantitative RT-PCR approach was used. Gene specific primers were designed in Primer 3 (http://frodo.wi.mit.edu/cgi-bin/primer3/primer3_www.cgi) and tested as well in amplify 3.1 (http://engels.genetics.wisc.edu/amplify). Target gene expressions were normalized to the expression of the reference gene ubiquitin in Poplar (Potri.015G013600) and Sorghum (Sb10g026870) and translation elongation factor in *R. irregularis*, respectively. All primers used are listed in **Table S1**. qRT-PCRs were run in a 7500 real-time PCR system (Roche) using the following settings: 95°C for 3 min and then 40 cycles of 95°C for 30 s, 60°C for 1 min and 72°C for 30 s. There were three biological and three technical replicates per treatment. Differences in gene expression between applied conditions were tested by a one-way ANOVA using SPSS Statistics, version 22 (IBM, Chicago, USA).

### RNA Sequencing and Data Analysis

Total RNA sequencing was done for three biological replicates per condition. Eighteen libraries were prepared and paired-end Illumina HiSeq mRNA sequencing (2x100bp RNA-Seq) was performed by Beckman Coulter Genomics France (Grenoble, France), which produced around 2 × 80 million reads per library in average. After quality check using FastQC, adaptor sequences were removed using FASTX-Toolkit. Only inserts of at least 30-nt were conserved for further analysis. Reads were mapped with CLC Genomics Workbench 11 (CLC Bio workbench, Qiagen, Aarhus, Denmark) using manufacturer’s recommendations on Poplar genome and gene annotation Ptrichocarpa_210_v3.0 (Tuskan et al., 2006), on Sorghum genome and gene annotation Sbicolor_313_v3.1 (McCormick et al., 2018), and on *R. irregularis* gene annotation Rhiir2-1 (Morin et al., 2019). The mapped reads for each transcript were calculated and normalized as RPKM for calculating gene expression (reads per kilobase of transcripts per million reads mapped—Mortazavi et al., 2008). Intact and broken pairs were both counted as one. The RPKMs of each transcript in different conditions were compared using proportion-based test statistics (Baggerly et al., 2003) implemented in CLC genomic Workbench suite. This beta-binomial test compares the proportions of counts in a group of samples against those of another group of samples. Different weights are given to the samples, depending on their sizes (total counts). The weights are obtained by assuming a Beta distribution on the proportions in a group, and estimating these, along with the proportion of a binomial distribution, by the method of moments. The result is a weighted *t*-type test statistic. We then calculated false discovery rate correction for multiple-hypothesis test (Benjamini and Hochberg, 1995). Only genes showing a difference of 10 reads between compared conditions were considered as significantly expressed. Genes were considered as differentially expressed when meeting the requirements of fold change ≥|2| and false discovery rate ≤ 0.05.The expression was then normalized using RPKM. Raw RNAseq data and global mapping data analyses were deposited at GEO (GSE138316).

### Metabolite Profiling and Data Analysis

For extraction of soluble metabolites about 90 mg of deep frozen poplar root and ERM samples (three biological replicates per condition) were pulverized in liquid N. Metabolite profiling was performed as described in (Dethloff et al., 2014) by gas chromatography coupled to electron impact ionization/time-of-flight mass spectrometry (GC-EI/TOF-MS) using an Agilent 6890N24 gas chromatograph (Agilent Technologies, Böblingen, Germany; http://www.agilent.com). Guidelines for manually supervised metabolite identification were the presence of at least 3 specific mass fragments per compound and a retention index deviation < 1.0% (Strehmel et al., 2008). For quantification purposes all mass features were evaluated for best specific, selective and quantitative representation of observed analytes. Laboratory and reagent contaminations were evaluated and removed according to non-sample control experiments. Metabolites were routinely assessed by log2-transformed relative changes expressed as response ratios. Statistical testing, namely two-way analysis of variance (ANOVA) and Wilcoxon–Mann–Whitney testing of significance were performed using relative abundances or log2-transformed ratios. Statistical assessments and data visualizations were performed using the multi-experiment viewer software, MeV (Version 4.9; http://www.tm4.org/mev.html; Saeed et al., 2006) and the Microsoft-Excel 2010 program.

## Results

### Colonization, and N and P Measurements

AM colonization of roots was between 79 and 87% (poplar) and about 93% (sorghum) (**Table S2**). Non-AM plants were not colonized. The hyphal colonization and the percentage of vesicles were not significantly different between low-P and high-P treatments in sorghum and poplar plants. However, significantly, sorghum roots contained three times more arbuscules in the low-P treatment, indicating that P starvation supported mycorrhization.

P treatment had significant effects on P content in the shoots and roots of poplar and sorghum (**Figure S2**). High-P treatment on ERM increased P content in roots and shoots, except for the roots from non-AM sorghum plants. Under the same P supply conditions, non-AM poplar accumulated more P than AM poplar whereas in AM sorghum, P accumulation was comparable to non-AM plants.

P treatment had significant effects on N content in the shoots of poplar, but marginally on sorghum (**Figure S2**). High-P treatment increased N content in poplar shoots. Under the same P supply conditions, AM poplar accumulated more P than non-AM poplar whereas in AM sorghum, N accumulation was comparable to non-AM plants.

### Patterns of Extraradical Mycelium and Intraradical Mycelium Expressomes in the Symbiocosm

#### Incidence of Phosphate Availability

**Figure 1:**
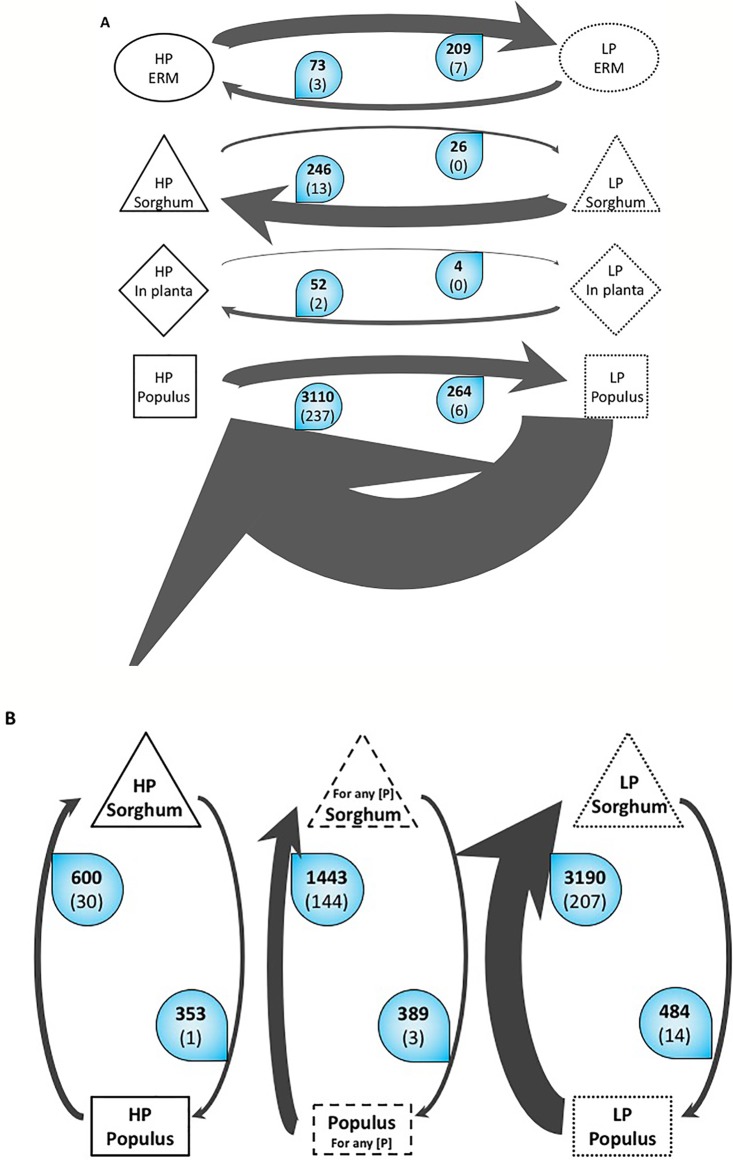
Overall variations of the number of Differentially Expressed Genes (DEG) of *R. irregularis* **(A)** Variations according to P. The arrows represent the number of differentially expressed genes in *R. irregularis*. Black bold numbers represent the number of genes with a fold change ≥2 and a false discovery rate ≤ 0.05. White numbers in brackets represent the number of genes involved in the transportome. The width of the arrow is proportional to the number of up-regulated genes. For example, the top arrow means that in the ERM grown in Low Phosphate, 209 genes are significantly up regulated at least two times compared to the ERM grown in High Phosphate. Among these 209 genes, seven are involved in the transportome. **(B)**. Variations according to host plant. The arrows represent number of differentially expressed genes in *R. irregularis* between the two compared conditions. Black bold numbers represent the number of genes with a fold change ≥2 and a false discovery rate ≤ 0.05. White numbers in brackets represent the number of genes involved in the transportome. The width of the arrow is proportional to the number of up-regulated genes. ERM, extraradical mycelium; HP, high phosphate; LP, low phosphate; *in planta*, intraradical mycelium of both sorghum and poplar.

The symbiotic part of the mycelium in roots (IRM) showed different gene expression patterns according to phosphate concentration and plant host. The total number of differentially expressed genes (DEG) differs around 10 fold between the two plants with 298 genes differentially regulated in *S. bicolor* plants against 3,162 in *P. trichocarpa* in response to phosphate treatment (**Figure 1A**). High phosphate concentration has a fewer incidence on the IRM transcriptome in *S. bicolor* than in *P. trichocarpa* whereas the fungus is connected to both plants, indicating that the allocation of mineral nutrients is more tightly regulated in *P. trichocarpa* than in *S. bicolor*. In both plants, the number of up regulated fungal genes is 10 times the number of down regulated fungal genes in high-P condition compared to low-P. This indicates that the fungus is metabolically more active in high-P condition, consistent with the fact that adding a high-P solution in the common mycorrhizal network compartment enhances the fungal-dependent Pi acquisition pathway. Only two fungal genes were significantly up regulated in both plants in response to high-P treatment and are involved in vesicular cargo. In Sorghum, the few up regulated genes were involved in vesicular trafficking, lipid binding, nitrate reduction and general transport. In *P. trichocarpa*, the genes up regulated by high-P belonged to vesicle metabolism, fatty acid metabolism and transport of N, Pi, sugars, and water. Interestingly, only one gene (a short chain dehydrogenase) was significantly up regulated in both plants and in the ERM in response to high-P treatment.

It is noteworthy that on the one hand, while cultivated in high-P, 3,408 genes were overexpressed in IRM (either in *S. bicolor*, or in *P. trichocarpa* or in both host plants) against 73 in the ERM compartment. On the other hand, while grown in low-P, the number of up regulated genes *in planta* dropped to 294 against 209 in the ERM. These overall expressome patterns suggest that when Pi is abundant in soil, the fungus invests little energy in recruiting the Pi but is highly active in exchanging this nutrient with the plants. On the opposite, when Pi is scarce in the medium, the extra radicle mycelium has to put a lot of energy in recovering the mineral in the soil but has fewer nutrients to exchange with the plants.

#### Incidence of Host Plant

We correlated the difference of DEG in the IRM in the two host plants connected by the same fungus at a given time point to the amount of nutrient resources that the fungus allocates to the two plants. Even though AM fungus has a very broad host range, their expressome differs according to host independently to P concentration (**Figure 1B**). In poplar, only 389 DEG were found among which only three are involved in the transportome (one vesicle transport and two transporters). In *S. bicolor*, a set of 1,443 fungal genes were found up-regulated, indicating active exchanges. A tenth of them (144) is involved in the transportome, representing all metabolisms (fatty acids, N, phosphate, sugars, potassium, general transporters, water, and vesicle transport). This difference indicates that AM symbiosis is more active and more tightly regulated in sorghum than in poplar (**Figure 1B**). The total P concentrations measured in the two mycorrhizal plants is consistent with this observation as more P was accumulated in mycorrhizal sorghum than in mycorrhizal poplar under low P fertilization (**Figure S2**).

In the mesocosm grown in low-P, the fungus does not allocate nutrients equally between the different host plants. The number of genes up regulated in *S. bicolor* is 6.5 times the number of genes overexpressed in *P. trichocarpa* (3,190 and 484 respectively). Not all these genes are supposed to be involved in trophic exchanges but the genes involved in transportome between the two plants showed a similar pattern. In *S. bicolor*, 207 fungal genes were up regulated, corresponding to all the different metabolisms and transporters we investigated (fatty acids, N, phosphate, sugars, transporters, water and vesicle transport, with the exception of potassium). In *P. trichocarpa* however, only 14 genes belonged to our transportome list and were involved in sugar transport, vesicle trafficking, and general transporters. In low-P condition, the AMF seems to favor the exchanges with the Sorghum at the expense of the Poplar. This observation correlates with the total phosphate measured in the two plants (**Figure S2**).

Under high-P, the difference in gene expression is lower than in low-P. We observe only a 2-fold difference between the number of over expressed genes in *S. bicolor* and *P. trichocarpa* (600 and 353 respectively). Moreover, the number of genes involved in transportome is much lower. Only one gene involved in vesicular trafficking is specifically expressed in *P. trichocarpa*. In *S. bicolor*, 30 genes involved in fatty acid metabolism, sugar transport, vesicle transport and general transport were recovered. The high-P makes it easily recoverable by the AM fungus and improves symbiotic exchanges with both plants, thus minimizing the preference for *S. bicolor* over *P. trichocarpa*. This balanced fungal transcriptome is supported by plant P concentration as the measurements in the two plants are very close, with a slight abundance in poplar.

### Regulation of Phosphate Transporter Expression

#### Gene Expression of Plant Pht1 Transporters

Using qRT-PCR, we measured the expression of the twelve Pht1 PT in roots and shoots of AM and non-AM poplar plants grown in high-P and low-P conditions. *PtPT8* and *PtPT10* were induced in AM-roots only, which implies an important role of these two transporters in symbiotic Pi uptake at the periarbuscular space (**Figure S3**).

Low-P treatment and mycorrhization induced expression of *PtPT1.1*, *PtPT1.2*, *PtPT1.4*, *PtPhT1.7*, and *PtPT1.11* in roots (**Figure S3B**). *PtPT1.2*, *PtPT1.4* and *PtPT1.11* were strongly induced in shoots, suggesting that these transporters are involved in intercellular Pi transfer and Pi transport over long distances, respectively (**Figure S3A**). *PtPT1.3* and *PtPT1.6* were neither expressed in roots nor in shoots. mRNA-Seq analysis independently confirmed our results for Pht1 expression (**Figure S4**).

In sorghum, *SbPT1.8* and *SbPT1.10* were induced in AM roots like *PtPT1.8* and *PtPT1.10* (**Figure S5**). *SbPT1.1*, *SbPT1.2*, *SbPT1.4*, *SbPT1.6*, and *SbPT1.7* were significantly induced in the non-AM low-P treatment. It seems that mycorrhization complemented sufficiently the P deficiency by increased Pi transfer to its host plant (**Figure S2**). In comparison to poplar Pht1, sorghum Pht1 was more susceptible to mycorrhization than to Pi concentration.

#### Gene Expression of Mycorrhizal Phosphate Transporters

**Figure 2:**
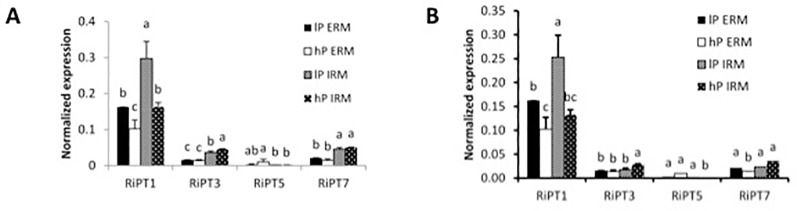
Quantification by qRT-PCR of the transcript abundances of phosphate transporters in *R. irregularis* when associated *with P. trichocarpa* **(A)** *and S. bicolor* **(B)**. Quantification of transcript levels of transporters in the ERM and in the IRM of the host plant under high (hP) and low (lP) availability of P. Values are means of three biological and three technical replicates. Error bars represent the SE. Translational elongation factor was used as a reference transcript. Statistical analysis was performed by analysis of variance for each gene, followed by Tuckey honest significant difference. Lower case letters indicate significant difference(Tukey’s t-test; p < 0.05).

In mycorrhizal poplar plants, qRT-PCR analysis of PT in the AM fungus *R. irregularis* in the ERM and IRM revealed expression of *RiPT1*, *RiPT3*, *RiPT5*, and *RiPT7*, with highest expression values for *RiPT1* (**Figure 2A**). *RiPT1* was significantly more expressed in the IRM compared to the ERM in low-P treatment. *RiPT1* expression was significantly higher under low-P treatment in the ERM and the IRM compared to high-P treatment. *RiPT7* tended to be highly expressed in the IRM compared to the ERM and *RiPT3* was lowly expressed in the ERM and induced in high-P treatment in the IRM. In Sorghum, we observed similar expression patterns except for *RiPT3* and *RiPT7*, significantly induced in the IRM than in the ERM (**Figure 2B**).

### Regulation of Transporters Involved in Nitrogen Exchange

#### Gene Expression of Plant Ammonium Transporters

**Figure 3:**
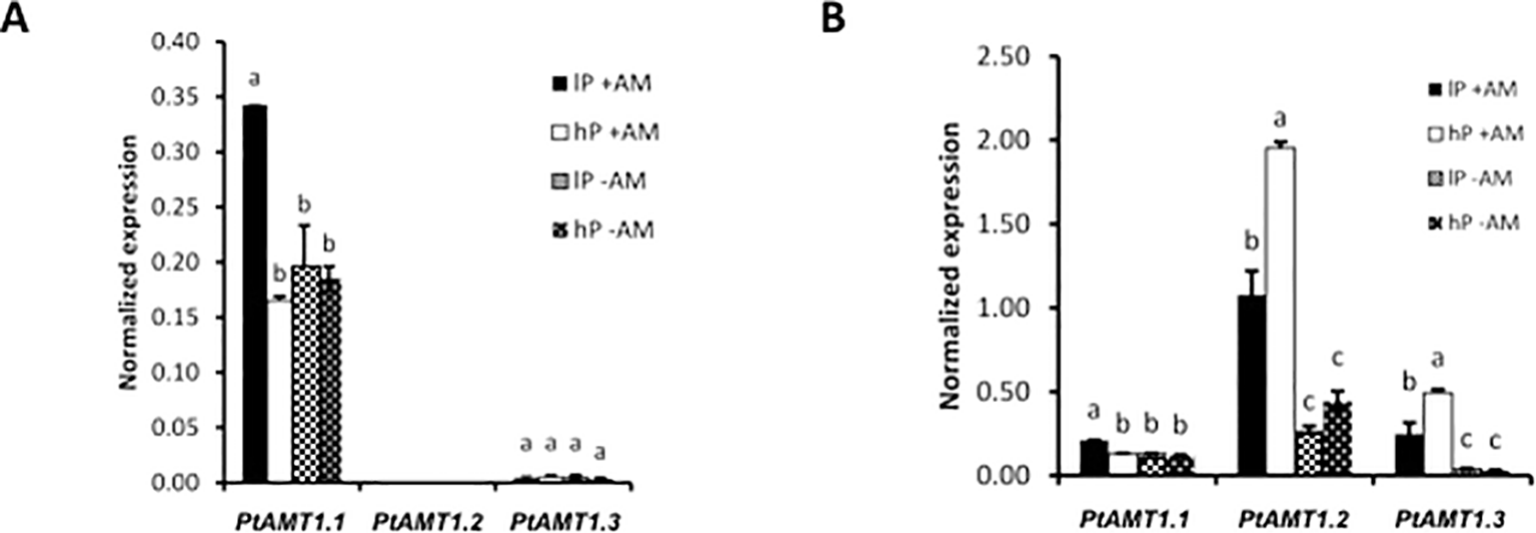
Quantification by qRT-PCR of the transcript abundances of the three ammonium transporters of poplar *PtAMT1.1*, *PtAMT1.2*, *and PtAMT3.1* in the shoot **(A)** and root **(B)** of mycorrhized (+AM) and non-mycorrhized (-AM) *P. trichocarpa* under low P (lP) and high P (hP) condition. Values are the means of three biological replicates and three technical replicates, each. Error bars represent the SE. Ubiquitin was used as reference transcript. Statistical analysis was performed by analysis of variance (ANOVA) per gene, followed by Tuckey honest significant difference test (Tuckey HSD; p < 0.05). Lower case letters indicate significant difference (Tukey’s t-test; p < 0.05).

As N is a major component of AM symbiosis, transcript abundances of AMTs in poplar were analyzed (**Figure S6**). The expression of three of them (*PtAMT1.1*, *PtAMT1.2*, and *PtAMT3.1*) was dependent of either mycorrhizal symbiosis of P-treatment or both. We then measured their expression in poplar by qRT-PCR. While *PtAMT1.1* and *PtAMT1.2* were described as induced in poplar upon mycorrhization with the ectomycorrhizal fungi *Paxillus involutus* (Couturier et al., 2007) and *Amanita muscaria* (Selle et al., 2005), no previous expression data were available for *PtAMT3.1*. Interestingly, *PtAMT3.1* clustered next to the three AM-inducible transporters GmAMT3.*1* (Kobae et al., 2010), *SbAMT3.1* (Koegel et al., 2013), and *OsAMT3.1* (Pérez-Tienda et al., 2014) (**Figure S6**). Here, *PtAMT1.1* was induced in the AM low-P treatment and the *PtAMT1.2* and *PtAMT3.1* were induced in the AM treatments (**Figure 3**). In addition, *PtAMT1.2* and *PtAMT3.1* were even higher expressed in the high-P condition, suggesting that both AMTs play a major role in symbiotic N transfer. Higher expression of these transporters under AM high-P condition might point to an increased N transfer when the AM fungus has access to more Pi. In shoots, we *PtAMT1.1* has a similar expression pattern as in roots. *PtAMT3.1* was only marginally expressed and *PtAMT1.2* was not expressed in leaves (**Figure 3**). mRNA-Seq confirmed our observations for AMT expression levels in the roots of Poplar (**Figure S6**). In a previous transcriptome study (Calabrese et al., 2017), in which AM poplar plants were exposed to N deficiency, *PtAMT4.1*, *PtAMT4.2*, and *PtAMT4.3* were AM-induced. Consistent with this previous study, we observed a specific induction of *PtAMT2.2*, *PtAMT4.1*, *PtAMT4.2*, and *PtAMT4.3* upon mycorrhization even though these transporters were expressed at lower levels compared to *PtAMT1.2* and *PtAMT3.1*.

In Sorghum, *SbAMT3.1* was specifically induced in AM-roots, and *SbAMT1.1* and *SbAMT1.2* were induced in the non-AM low-P treatment (**Figure S7**). However, *SbAMT1.1* and *SbAMT1.2* were nearly twice more expressed in shoots compared to roots.

#### Gene Expression Ammonium Transporters in *Rhizophagus Irregularis*

**Figure 4:**
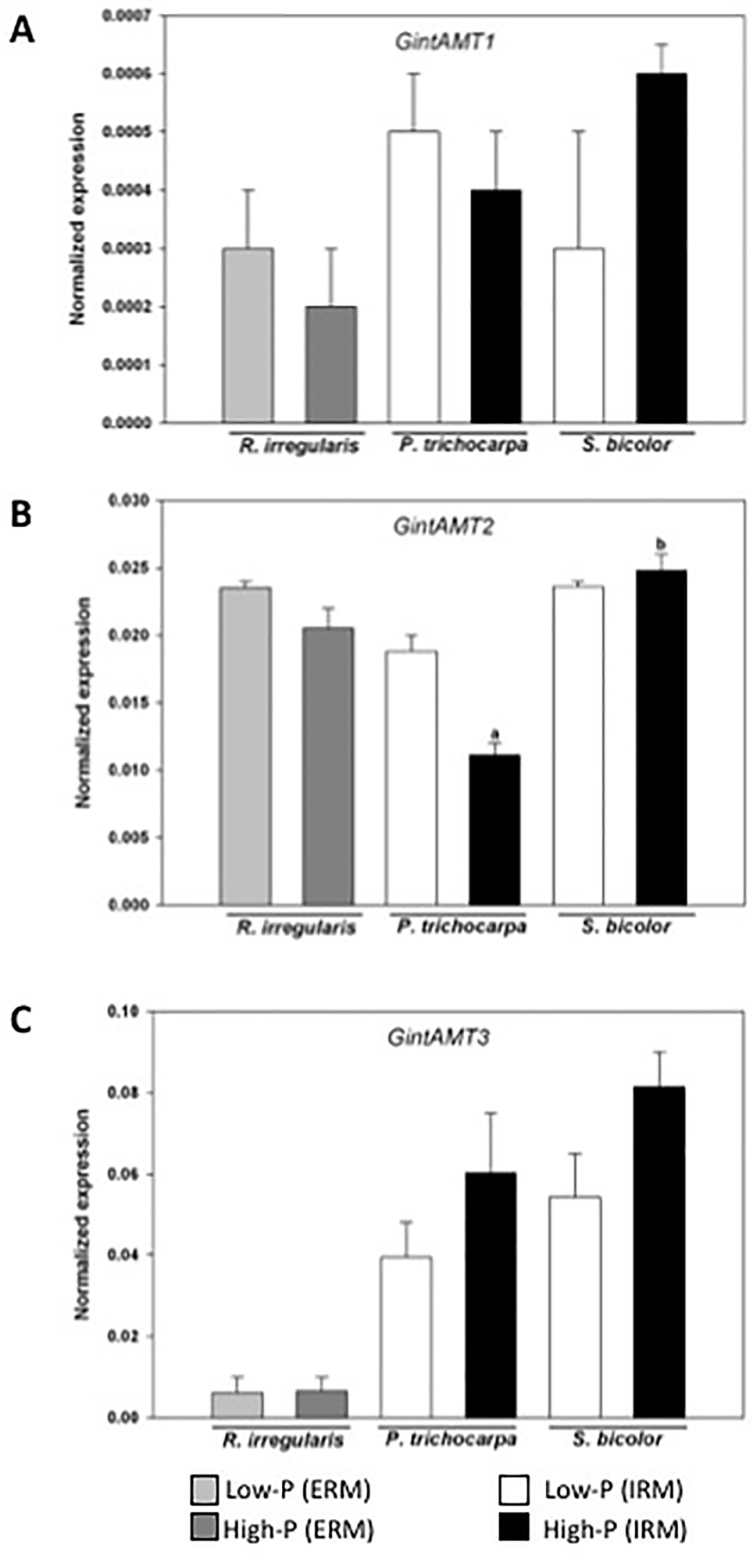
Quantification by qRT-PCR of the transcript abundances of ammonium transporters in *R. irregularis*. Transcript abundances were measured under low-P and high-P availability. Transcript abundances of the three transporters GintAMT1 (A), GintAMT2 (B), and GintAM3 (C) were measured in the extra-radical mycelium (ERM) and in the intraradical mycelium (IRM) when associated to the host plants poplar and sorghum. Values are means of three biological and three technical replicates. Translational elongation factor was used as a reference transcript. Statistical analysis was performed by analysis of variance for each gene, followed by Tuckey honest significant difference test (Tuckey HSD; p < 0.05). Lower case letters indicate significant difference(Tukey’s t-test; p < 0.05).

Quantitative expression analysis of the three AMTs in the AM fungus revealed that their expression is not significantly different in the ERM between the low-P and high-P treatments. The expression of *GintAMT3* was significantly higher in the IRM compared to the ERM (Calabrese et al., 2017). *GintAMT2* and *GintAMT1* were equally expressed in the ERM and IRM in poplar and sorghum (**Figure 4**). *GintAMT2* was significantly highly expressed in the IRM of sorghum compared to poplar. Specific induction of *GintAMT3* in the IRM might indicate a possible localization of the transporter at the arbuscular side for the transfer of ammonium to the periarbuscular side to enable ammonium uptake for the plant.

#### Gene Expression of Amino Acid and H+/Oligopeptide Transporters

In our study, we identified several amino acid transporters and H+/oligopeptide symporters that were either specifically induced or repressed upon mycorrhization (**Table S3**). Here, root colonization highly induced expression of two H^+^/oligopeptide transporters. Potri.005G233500, a homologous gene of *AtPTR1* shown to transport di-/tripeptides with low selectivity in *Arabidopsis thaliana*, was highly induced. AtPTR1 is situated in the plasma membrane of vascular tissue which indicates a role in long-distance transport (Dietrich et al., 2004). *AtPTR3*, a homologue of Potri.002G258900, was induced upon salt stress and was shown to be regulated by methyl jasmonate, salicylic acid and abscisic acid. Further AtPTR3 was induced upon inoculation of the plant with pathogens. A reduced activation of AtPTR3 in *hrpA* mutant indicated that it is a defense related gene protecting the plant against abiotic and biotic stress (Karim et al., 2005; Karim et al., 2006). The differential expression of these transporters upon mycorrhization may suggest a role of the transporters in N uptake but also a role in AM root colonization.

### Gene Expression Related to Carbon Exchange

#### Gene Expression of Sugar Transporters

**Figure 5:**
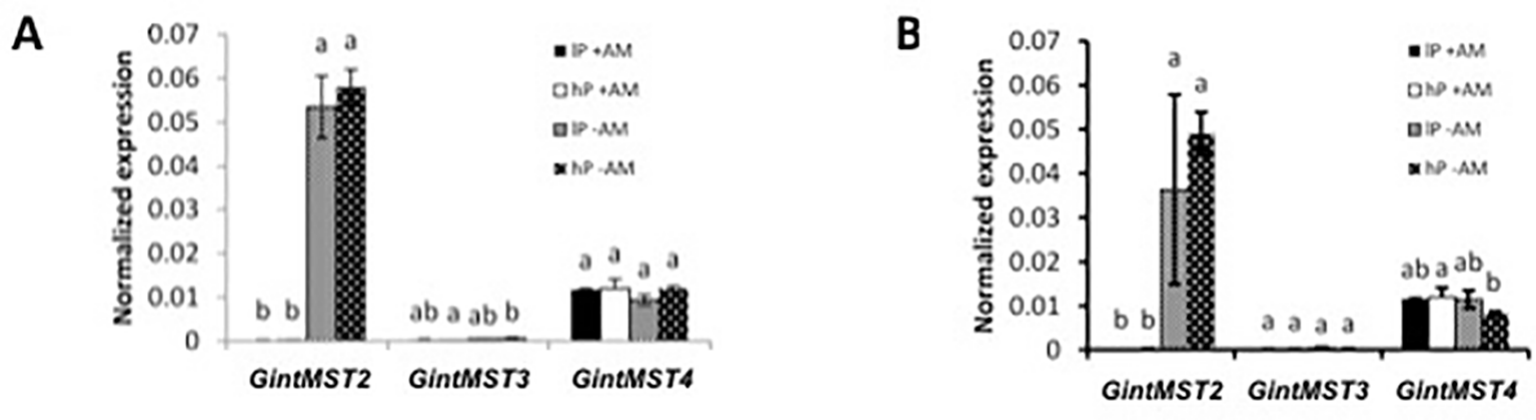
Quantification by qRT-PCR of the transcript abundances of monosaccharide transporters (MST) in *R. irregularis when associated with P. trichocarpa* **(A)** *and S. bicolor* **(B)**. Quantification of transcript levels of transporters in the ERM and in the IRM of the host plant under high (hP) and low (lP) availability of P. Values are means of three biological and three technical replicates. Error bars represent the SE. Translational elongation factor was used as a reference transcript. Statistical analysis was performed by analysis of variance for each gene, followed by Tuckey honest significant difference. Lower case letters indicate significant difference(Tukey’s t-test; p < 0.05).

Quantitative expression analysis using qRT-PCR of five SUT (*SUT1* and *SUT3* to *SUT6*) in poplar revealed that all were expressed (**Figures S8** and **S9**). *SUT1* was only marginally expressed while *SUT4* was strongly expressed in roots and shoots. While *SUT1* and *SUT4* were not differentially expressed upon mycorrhization, surprisingly, *SUT3* was down-regulated upon mycorrhization in roots and shoots of poplar. Interestingly, *SUT6* was down-regulated in shoots by mycorrhization. In sorghum, *SUT1* was also down-regulated (**Figure S10**). In Poplar, we screened our transcriptome data set for other carbohydrate transporters. We found three carbohydrate transporters induced and four repressed upon mycorrhization (**Table S3**). Among them, only the transporter/spinster transmembrane protein Potri.001G286600 presents characteristics for plasma membrane localization. The UDP-galactose transporter related proteins are localized at the lumen of the Golgi cisternae (Norambuena et al., 2002). On the fungal side, the MST *GintMST2* was specifically induced in the IRM of mycorrhizal poplar and sorghum and its expression was not affected by P concentration (**Figure 5**). The induction of *GintMST2* gene expression suggested a role in symbiotic carbohydrate transfer but functional properties still need to be determined. The down-regulation of plant MSTs expression in mycorrhizal conditions whereas the fungal *GintMST2* is still expressed in the IRM indicate that the AM fungus obtains the sugar from the intercellular space without the cooperation of the plant itself, turning the AM root into a sink for sugars.

#### Expression of Fatty Acid Genes

Expression of different genes involved in fatty acid synthesis and transfer were screened. The expression of these genes is mandatory in order to accommodate the AM fungus and allow the formation of the arbuscules. *Ram2*, *FatM*, and *STR1* were specifically expressed in mycorrhizal Sorghum and *STR2*, *CCaMK*, *RAD1*, *Cyclops*, and *Castor*/*pollux* were upregulated when the plant was in symbiosis (**Table S4**). These genes followed the same pattern of expression in poplar either in low-P or in high-P condition.

On the fungal side, no fatty acid transporter has been identified yet, suggesting another form of acquisition. However, many genes involved in fatty acid metabolism were overexpressed in the different plants. Multiple lipases and lipid recognition proteins are over expressed *in planta* compared to the ERM (**Table S5**). These might play a role both in managing the TAG stock or acquiring fatty acids from the host plant. Three desaturases and one elongase show a slight increase in expression *in planta* compared to *ex planta*. Pi addition had little effect on the ERM but increased the expression of several lipases *in planta*.

### Primary Metabolism of Poplar and Sorghum Roots, and the ERM of *R. Irregularis*

To gain further insights into the changes of root primary metabolism caused by the interaction between poplar, sorghum and *R. irregularis* we conducted GC-MS metabolite profiling experiments. Under our experimental conditions, the ERM metabolite profile of *R. irregularis* was not significantly affected by P conditions (**Tables 1** and **S6**). In the ERM, we observed slight but mostly non-significant increases of organic acids, glucose, trehalose, glycine and of amino acids with branched aliphatic side chains, with leucine as the only significant increase in our experiment (**Tables 1** and **S6**).

**Table 1:** Relative abundances of metabolites detected in the ERM of *R. irregularis* in response to low (lP) and high P (hP) concentrations.

Class	Name	log2 ratio lP vs hP	p-value
Acids	Aconitic acid, cis-	-0.53	0.275
Acids	Benzoic acid	0.02	0.827
Acids	Benzoic acid, 4-hydroxy-	0.04	0.827
Acids	Citric acid	0.25	0.827
Acids	Fumaric acid	0.36	0.513
Acids	Isocitric acid	0.32	0.513
Acids	Lactic acid	-0.34	0.275
Acids	Malic acid	0.60	0.275
Acids	Pyruvic acid	0.11	0.275
Acids	Quinic acid	-1.88	0.513
Acids	Succinic acid	0.26	0.827
Alcohols	Benzylalcohol	-0.18	0.513
Amino Acids	Aspartic acid	-0.24	0.513
Amino Acids	Glutamic acid	-0.44	0.513
Amino Acids	Glycine	0.80	0.275
Amino Acids	Isoleucine	0.63	0.513
Amino Acids	Leucine	0.19	**0.050**
Amino Acids	Lysine	0.11	0.564
Amino Acids	Ornithine	-1.28	0.127
Amino Acids	Phenylalanine	-0.12	0.827
Amino Acids	Pyroglutamic acid	-0.06	0.513
Amino Acids	Serine	0.19	0.827
Amino Acids	Valine	0.62	0.513
N- Compounds	Ethanolamine	0.15	0.275
N- Compounds	Putrescine	0.14	0.513
N- Compounds	Pyridine, 2-hydroxy-	0.02	0.827
Phenylpropanoids	Caffeic acid, trans-	-0.03	1.000
Phosphates	Phosphoric acid	0.25	0.513
Phosphates	Phosphoric acid monomethyl ester	-0.06	0.827
Polyols	Arabitol	-0.07	0.827
Polyols	Glycerol	-0.24	0.513
Polyols	Inositol, myo-	0.05	0.827
Polyols	Mannitol	-0.92	0.513
Sugar Conjugates	Galactinol	-2.29	0.248
Sugar Conjugates	Salicin	-4.25	1.000
Sugars	Glucose	0.41	0.127
Sugars	Glucose, 1,6-anhydro-, beta-	0.11	0.513
Sugars	Rhamnose	0.16	0.513
Sugars	Ribose	-0.32	0.564
Sugars	Sucrose	-0.06	0.827
Sugars	Trehalose, alpha,alpha’-	0.39	0.127

Data was log transformed and tested by Wilcoxon rank sum test (p < 0.05, n = 3) in MeV v4.9 (http://www.tm4.org/mev.html). Significant p-values are highlighted in bold.

In contrast, non-AM low-P treatment on poplar roots increased general organic acid and amino acid pools and coincidently decreased pools of glucose-6-phosphate or fructose-6-phosphate (**Tables 2** and **S7**). Analysis of variance indicated significant general accumulation of only few metabolites, *i.e.*, 4-amino-butanoic acid (GABA), isoleucine, phenylalanine, serine, threonic acid, ribonic acid, and arabinonic acid-1,4-lactone independently of the mycorrhizal status (**Tables 2** and **S7**). AM colonization enhanced nutrient acquisition in the low-P treatment (**Tables 2** and **S7**). In addition, mycorrhization modified the root primary metabolism under low-P and high-P and caused a general decrease of the metabolite pools. Thirty-eight out of 79 monitored primary metabolite pools were decreased with only one exception besides few still non-identified metabolites, namely trehalose (**Tables 2** and **S7**). Trehalose is a major storage carbohydrate of AM fungi (Bécard et al., 1991). Mycorrhization not only decreased the main organic acids of the TCA cycle, e.g., malic acid, aconitic acid, 2-oxo-glutaric acid, succinic acid, and fumaric acid, but also many amino acids including aspartic and glutamic acid, as well as phenylalanine, glycine, serine, leucine, isoleucine and valine. In addition, mycorrhization decreased the glucose-6-phosphate, fructose-6-phosphate, *myo*-inositol, and galactinol pools and additional carbohydrates including maltose. For sorghum, the effect of P treatment on AM colonized plants was studied only. Compared to poplar, AM sorghum plants are slightly affected by the low-P treatment; we observed only a slight significant decrease of fructose-6-phosphate, glucose-6-phosphate, and phosphoric acid under low-P treatment (**Table 3**).

**Table 2:** Relative abundances of metabolites detected in poplar roots. Abundances were measured in the mycorrhized (+AM) and non-mycorrhized (-AM) poplar roots under high (hP) and low (lP) P availability.

Class	Name	log2 ratios					2-way ANOVA		
		+AM vs -AM	lP+AM vs hP+AM	lP-AM vs hP -AM	lP+AM vs lP -AM	hP +AM vs hP-AM	Effect of mycorrhization	Effect of p-availability	Effect of interaction

Acids	Aconitic acid, cis-	-1.62	-0.01	-0.17	-1.54	-1.69	**0.006**	0.863	0.934
Acids	Benzoic acid	-0.63	-0.18	0.72	-1.03	-0.13	**0.013**	0.182	**0.034**
Acids	Benzoic acid, 3,4-dihydroxy-	-0.16	-0.09	0.13	-0.27	-0.05	0.750	0.839	0.602
Acids	Benzoic acid, 4-hydroxy-	0.09	-0.10	0.83	-0.32	0.62	0.767	0.426	0.162
Acids	Citric acid	0.46	0.06	0.65	0.20	0.78	0.054	0.125	0.172
Acids	Fumaric acid	-0.87	0.25	0.34	-0.92	-0.82	**0.038**	0.325	0.814
Acids	Glutaric acid, 2-hydroxy-	-1.59	-0.28	0.58	-2.00	-1.14	**0.003**	0.546	0.420
Acids	Glutaric acid, 2-oxo-	-1.70	0.18	1.64	-2.21	-0.75	**0.009**	0.111	0.180
Acids	Glutaric acid, 3-hydroxy-3-methyl-	-0.87	-0.37	0.80	-1.41	-0.24	0.065	0.549	0.199
Acids	Isocitric acid	0.57	0.18	0.86	0.29	0.97	0.054	0.084	0.190
Acids	Lactic acid	-0.74	-0.68	1.36	-1.65	0.39	0.083	0.389	**0.013**
Acids	Malic acid	0.07	-0.21	0.72	-0.35	0.57	0.958	0.412	0.135
Acids	Malic acid, 2-isopropyl-	ND	ND	1.04	ND	ND	ND	ND	ND
Acids	Malic acid, 2-methyl-	ND	ND	0.85	ND	ND	ND	ND	ND
Acids	Pyruvic acid	0.31	0.05	0.29	0.20	0.44	0.147	0.400	0.454
Acids	Quinic acid	-0.15	-0.79	0.92	-0.98	0.72	0.895	0.723	0.092
Acids	Shikimic acid	-2.32	-0.73	0.82	-3.08	-1.53	**0.000**	0.658	**0.008**
Acids	Succinic acid	-0.85	0.04	0.72	-1.14	-0.46	**0.032**	0.212	0.552
Acids	Vanillic acid	-0.29	0.18	0.51	-0.44	-0.10	0.353	0.258	0.631
Alcohols	Benzylalcohol	-0.37	-0.09	0.95	-0.81	0.23	0.479	0.180	0.104
Amino Acids	Aspartic acid	-4.52	0.82	0.59	-4.43	-4.66	**0.000**	0.134	0.845
Amino Acids	Butanoic acid, 4-amino-	-3.03	0.68	0.51	-2.96	-3.13	**0.000**	**0.032**	0.572
Amino Acids	Glutamic acid	-2.87	1.27	-0.85	-1.88	-4.01	**0.001**	0.574	0.090
Amino Acids	Glycine	-0.92	0.44	0.63	-1.01	-0.80	**0.023**	0.112	0.736
Amino Acids	Isoleucine	-1.75	0.51	0.66	-1.81	-1.67	**0.000**	**0.041**	0.873
Amino Acids	Leucine	-1.25	-0.06	0.34	-1.44	-1.04	**0.001**	0.483	0.306
Amino Acids	Phenylalanine	-3.55	0.76	0.65	-3.51	-3.61	**0.000**	**0.048**	0.964
Amino Acids	Pyroglutamic acid	-1.45	0.24	-0.05	-1.31	-1.60	**0.001**	0.532	0.762
Amino Acids	Serine	-1.09	0.45	0.58	-1.14	-1.02	**0.000**	**0.014**	0.765
Amino Acids	Valine	-2.32	-0.09	0.78	-2.71	-1.83	**0.000**	0.224	0.118
Aromatic	Catechol	-1.33	1.43	1.85	-1.43	-1.00	0.245	0.276	0.598
N- Compounds	Ethanolamine	-0.05	-0.16	-0.02	-0.13	0.02	0.927	0.722	0.594
N- Compounds	Phenol, 2-amino-	-1.28	0.26	0.87	-1.53	-0.91	**0.019**	0.242	0.306
N- Compounds	Putrescine	0.19	0.02	0.19	0.11	0.27	0.362	0.499	0.616
N- Compounds	Pyridine, 2-hydroxy-	-0.08	-0.33	0.34	-0.41	0.25	0.803	0.872	0.161
Phenylpropanoids	Caffeic acid, cis-	0.15	0.03	0.91	-0.21	0.66	0.298	0.070	0.075
Phenylpropanoids	Caffeic acid, trans-	-0.05	0.06	1.14	-0.48	0.60	0.666	0.056	0.070
Phenylpropanoids	Cinnamic acid, 4-hydroxy-	ND	ND	0.60	ND	-0.47	ND	ND	ND
Phenylpropanoids	Epicatechin	-0.14	-0.16	0.60	-0.50	0.26	0.668	0.323	0.138
Phenylpropanoids	Ferulic acid, trans-	-0.12	0.10	0.94	-0.47	0.37	0.703	0.066	0.121
Phenylpropanoids	Quinic acid, 3-caffeoyl-, cis-	1.01	0.16	0.07	1.05	0.96	0.102	0.745	0.572
Phenylpropanoids	Quinic acid, 3-caffeoyl-, tran	0.80	0.13	0.42	0.68	0.96	0.155	0.487	0.347
Phosphates	Fructose-6-phosphate	-1.85	-0.51	-0.22	-2.01	-1.73	**0.003**	0.420	0.806
Phosphates	Glucose-6-phosphate	-2.22	-1.10	-0.43	-2.64	-1.97	**0.000**	0.065	0.453
Phosphates	myo-Inositol-phosphate	ND	ND	-1.11	-1.17	ND	ND	ND	ND
Phosphates	Phosphoric acid	-3.48	0.45	-2.86	-1.22	-4.54	**0.042**	0.634	0.468
Phosphates	Phosphoric acid monomethyl ester	-2.35	-0.39	-0.05	-2.53	-2.20	**0.001**	0.648	0.676
Polyhydroxy Acids	Arabinonic acid-1,4-lactone	-0.18	0.51	1.57	-0.52	0.53	0.914	**0.001**	**0.041**
Polyhydroxy Acids	Galactaric acid	-1.18	-0.25	0.81	-1.66	-0.60	**0.011**	0.455	0.209
Polyhydroxy Acids	Galactonic acid	-1.45	-0.63	0.64	-2.08	-0.81	**0.002**	0.963	0.100
Polyhydroxy Acids	Gluconic acid	-1.14	-0.22	1.22	-1.73	-0.29	**0.018**	0.217	0.139
Polyhydroxy Acids	Glyceric acid	-1.62	-0.28	0.20	-1.87	-1.38	**0.000**	0.900	0.238
Polyhydroxy Acids	Gulonic acid	-0.36	0.04	0.62	-0.61	-0.03	0.425	0.257	0.311
Polyhydroxy Acids	Lyxonic acid-1,4-lactone	-0.11	0.30	0.91	-0.35	0.26	0.772	0.080	0.292
Polyhydroxy Acids	Ribonic acid	-1.15	0.42	0.51	-1.19	-1.10	**0.001**	**0.048**	0.686
Polyhydroxy Acids	Saccharic acid	-1.77	0.01	1.37	-2.30	-0.94	**0.001**	0.065	**0.039**
Polyhydroxy Acids	Threonic acid	-2.04	0.50	1.05	-2.24	-1.69	**0.000**	**0.019**	0.292
Polyols	Arabitol	-1.19	-0.20	0.26	-1.42	-0.97	**0.004**	0.722	0.471
Polyols	Galactitol	-0.16	0.54	-0.46	0.33	-0.67	0.507	0.853	0.143
Polyols	Glycerol	0.17	-0.86	-0.12	-0.27	0.48	0.927	0.307	0.475
Polyols	Inositol, myo-	-2.84	0.98	0.26	-2.56	-3.28	**0.000**	0.123	0.422
Polyols	Mannitol	-0.23	0.26	-0.86	0.39	-0.74	0.896	0.895	0.468
Sugar Conjugates	Galactinol	-2.34	0.70	-0.99	-1.45	-3.14	**0.010**	0.829	0.391
Sugar Conjugates	Salicin	-0.94	0.72	1.58	-1.21	-0.35	0.193	0.137	0.275
Sugar Conjugates	Salicylic acid-glucopyranoside	-2.48	-0.06	-1.13	-1.84	-2.90	**0.038**	0.913	0.998
Sugars	Arabinose	-1.21	-0.18	0.38	-1.48	-0.92	**0.008**	0.776	0.575
Sugars	Fructose	0.65	0.30	> 0.01	0.79	0.49	0.300	0.769	0.986
Sugars	Galactose	0.22	-0.38	0.64	-0.26	0.75	0.984	0.813	0.615
Sugars	Glucose	0.18	0.23	-0.23	0.41	-0.05	0.728	0.791	0.941
Sugars	Glucose, 1,6-anhydro-, beta-	-0.75	-0.13	0.70	-1.13	-0.29	**0.001**	0.076	**0.018**
Sugars	Maltose	-1.35	0.36	0.85	-1.55	-1.05	**0.006**	0.139	0.622
Sugars	Mannose	0.05	-0.68	-0.03	-0.32	0.33	0.800	0.791	0.866
Sugars	Raffinose	-1.59	1.23	-4.14	2.12	-3.25	0.602	0.766	0.173
Sugars	Rhamnose	-1.00	-0.04	0.48	-1.24	-0.72	**0.009**	0.674	0.351
Sugars	Ribose	-1.96	0.13	0.89	-2.28	-1.52	**0.001**	0.258	0.258
Sugars	Sucrose	0.10	-0.02	0.11	0.04	0.17	0.534	0.669	0.658
Sugars	Trehalose, alpha,alpha’-	1.20	-0.49	-0.18	1.03	1.33	**0.046**	0.883	0.841
Sugars	Xylose	-0.50	-0.12	0.94	-0.95	0.10	0.639	0.526	0.363
Sugars	Xylulose	ND	ND	1.14	-2.36	ND	ND	ND	ND

Data was log transformed and tested by y 2-way analysis of variance (ANOVA) (p < 0.05, n = 3) in MeV v4.9 (http://www.tm4.org/mev.html). Significant p-values are highlighted in bold. Log2 values >1 and <-1 are color-marked (blue and orange, respectively) if p < 0.05.

**Table 3:** Relative abundances of metabolites detected in sorghum roots. Abundances were measured in the mycorrhized (+AM) sorghum roots under high (hP) and low (lP) P availability.

Class	Name	log2 ratios	2-way ANOVA
		lP+AM vs hP+AM	Effect of p-availability
Acids	Aconitic acid, cis-	0.31	**0.04**
Acids	Benzoic acid	0.97	0.51
Acids	Benzoic acid. 3,4-dihydroxy-	0.63	0.22
Acids	Benzoic acid, 4-hydroxy-	0.98	0.51
Acids	Citric acid	1.30	0.51
Acids	Fumaric acid	1.34	0.51
Acids	Glutaric acid, 2-hydroxy-	1.44	**0.04**
Acids	Glutaric acid, 2-oxo-	0.97	0.51
Acids	Glutaric acid, 3-hydroxy-3-methyl-	ND	ND
Acids	Isocitric acid	1.29	0.51
Acids	Lactic acid	2.08	0.13
Acids	Malic acid	1.19	0.83
Acids	Malic acid, 2-isopropyl-	ND	ND
Acids	Malic acid, 2-methyl-	ND	ND
Acids	Pyruvic acid	1.10	0.51
Acids	Quinic acid	1.22	0.83
Acids	Shikimic acid	1.71	0.28
Acids	Succinic acid	2.21	0.51
Acids	Vanillic acid	1.10	0.83
Alcohols	Benzylalcohol	1.18	0.51
Amino Acids	Aspartic acid	1.97	0.51
Amino Acids	Butanoic acid, 4-amino-	2.32	0.28
Amino Acids	Glutamic acid	1.30	0.51
Amino Acids	Glycine	1.03	0.83
Amino Acids	Isoleucine	1.05	0.83
Amino Acids	Leucine	1.13	0.22
Amino Acids	Phenylalanine	1.13	0.83
Amino Acids	Pyroglutamic acid	0.91	0.28
Amino Acids	Serine	1.01	0.51
Amino Acids	Valine	1.14	0.83
Aromatic	Catechol	ND	ND
N- Compounds	Ethanolamine	1.11	0.83
N- Compounds	Phenol, 2-amino-	ND	ND
N- Compounds	Putrescine	1.08	0.83
N- Compounds	Pyridine, 2-hydroxy-	1.15	0.83
Phenylpropanoids	Caffeic acid, cis-	ND	ND
Phenylpropanoids	Caffeic acid, trans-	1.22	0.28
Phenylpropanoids	Cinnamic acid, 4-hydroxy-, trans-	1.19	0.83
Phenylpropanoids	Epicatechin	2.13	0.51
Phenylpropanoids	Ferulic acid, trans-	ND	ND
Phenylpropanoids	Quinic acid, 3-caffeoyl-, cis-	1.48	0.83
Phenylpropanoids	Quinic acid, 3-caffeoyl-, trans-	7.08	0.22
Phosphates	Fructose-6-phosphate	0.63	**0.04**
Phosphates	Glucose-6-phosphate	0.61	**0.04**
Phosphates	myo-Inositol-phosphate	ND	ND
Phosphates	Phosphoric acid	0.69	**0.04**
Phosphates	Phosphoric acid monomethyl ester	0.75	0.28
Polyhydroxy Acids	Arabinonic acid-1,4-lactone	ND	ND
Polyhydroxy Acids	Galactaric acid	0.73	0.28
Polyhydroxy Acids	Galactonic acid	1.06	0.83
Polyhydroxy Acids	Gluconic acid	0.70	0.51
Polyhydroxy Acids	Glyceric acid	1.34	0.22
Polyhydroxy Acids	Gulonic acid	ND	ND
Polyhydroxy Acids	Lyxonic acid-1,4-lactone	1.50	0.83
Polyhydroxy Acids	Ribonic acid	1.12	0.51
Polyhydroxy Acids	Saccharic acid	1.34	0.51
Polyhydroxy Acids	Threonic acid	0.94	0.83
Polyols	Arabitol	0.53	0.28
Polyols	Galactitol	2.07	0.51
Polyols	Glycerol	1.08	0.83
Polyols	Inositol, myo-	1.26	0.83
Polyols	Mannitol	0.71	0.51
Sugar Conjugates	Galactinol	1.67	0.28
Sugar Conjugates	Salicin	1.05	0.51
Sugar Conjugates	Salicylic acid-glucopyranoside	ND	ND
Sugars	Arabinose	1.06	0.51
Sugars	Fructose	0.86	0.83
Sugars	Galactose	1.71	0.51
Sugars	Glucose	1.47	0.83
Sugars	Glucose, 1,6-anhydro-, beta-	1.09	0.51
Sugars	Maltose	2.33	0.22
Sugars	Mannose	ND	ND
Sugars	Raffinose	1.21	0.83
Sugars	Rhamnose	0.54	0.05
Sugars	Ribose	1.48	0,51
Sugars	Sucrose	1.18	0.51
Sugars	Trehalose, alpha,alpha’-	0.93	0.83
Sugars	Xylose	2.44	0.51
Sugars	Xylulose	2.17	1.00

Data was log transformed and tested by Wilcoxon rank sum test (p < 0.05, n = 3) in MeV v4.9 (http://www.tm4.org/mev.html). Significant p-values are highlighted in bold. Log2 values >1 are color-marked in blue if p < 0.05.

## Discussion

In this study, we described the effects of P supply and AM colonization on PTs, AMTs, carbohydrate transporters and lipid genes in poplar and sorghum, and in the ERM and IRM of the AM fungus *R. irregularis*.

### Differential Resource Allocation in the Common Mycorrhizal Network

In the symbiocosm, we observed disequilibrium in fungal DEG patterns according to sorghum and poplar symbiotic tissues in response to high-P concentration supplied to ERM. Sorghum is an annual grass with a C4 metabolism, and poplar a perennial tree with a C3 metabolism. The fungal transportome in the fast growing Poaceae is always more important than in poplar in any given P concentration. It has been shown that the fungus is able to adapt its resource allocation to the best symbiont available (Fellbaum et al., 2014; Whiteside et al., 2019). Our data provide same conclusion at the transcriptomic level. However, we have no information about the extent of the ERM developed from roots of each of the two host plants, so differences observed between the two hosts may depend also on a differential ability of the symbiont to form functional ERM. Anyway, we could hypothesize that the perennial poplar plant is less mycorrhiza-dependent than the annual sorghum.

### Symbiotic Phosphorus Exchange

**Figure 6:**
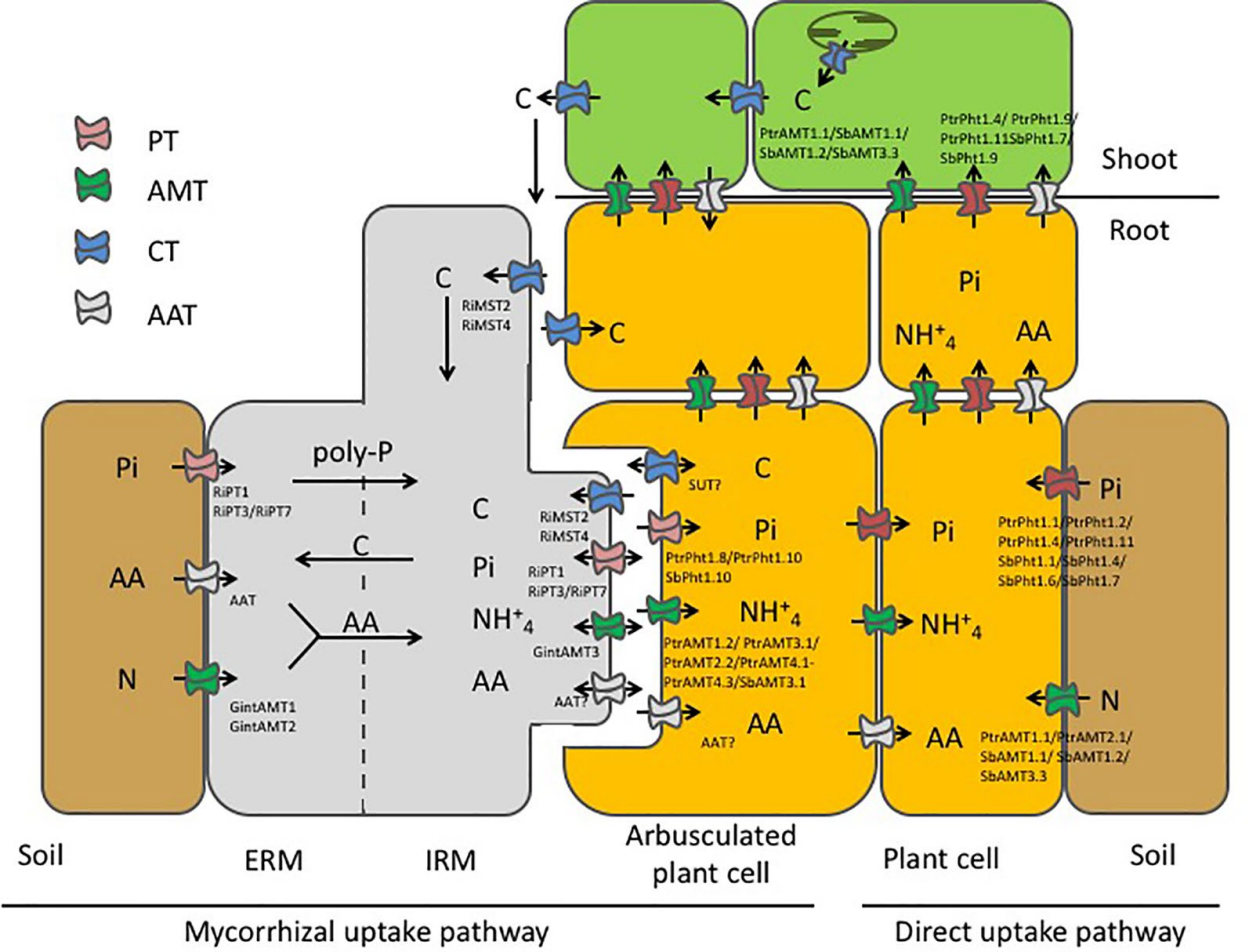
Schematic representation of the mycorrhizal nutrient uptake pathway and the direct nutrient uptake pathway in our model systems poplar and sorghum when colonized by *R. irregularis*. In the direct uptake pathway (right hand side) nutrients, *i.e.* inorganic phosphate (Pi) and nitrogen (N) are taken up from the rhizosphere by phosphate transporters (PT) and ammonium transporters (AMT) and are transported to the shoot. In symbiotic interaction, the AMF partially takes over nutrition of the plant. Nutrients are taken up by specialized transporters in the extraradical mycelium (ERM) and are further transported to the intraradical mycelium (IRM) where they are transferred to the periarbuscular space, to be taken up by plant transporters. N has been suggested to be additionally taken up from the soil in form of amino acids (AA) by predicted amino acid transporters (AAT). In exchange for the transfer of the mineral nutrients the mycorrhizal fungus is rewarded with essential carbohydrates from the plant. As some transporters were specifically induced by mycorrhization a possible localization at the periarbuscular membrane was assumed for the plant transporters. High induction of mycorrhizal transporters in the IRM compared to the extraradical mycelium ERM suggest that these transporters are mainly involved in nutrient exchange at the symbiotic interface. The reality is probably much more complex, with reuptake of nutrients (double-headed arrow) at the biotrophic interface, allowing both partners to, at least partially, control the exchanges.

In our study, we confirmed the expression of *PtPT1.10* in Poplar AM roots only (**Figure S3**) as already reported (Loth-Pereda et al., 2011). In addition, *PtPT1.8* was also expressed in AM roots only. In sorghum roots, AM colonization induced the specific expression of *SbPT1.8*, *SbPT1.10* and partially of *SbPT1.11* (**Figure S5**). The specific induction of *PtPT1.8* and *PtPT1.10* in poplar and of *SbPT1.8*, *SbPT1.10* in sorghum upon mycorrhization strongly suggested that there is a symbiosis-dependent Pi uptake system. In *M. truncatula*, it has been shown that *MtPT4* is specifically expressed in the periarbuscular membrane (Harrison et al., 2002). The specific induction of *PtPT1.8* and *PtPT1.10* in poplar and of *SbPT1.8*, *SbPT1.10* in sorghum suggested that these transporters are localized at the periarbuscular membrane (**Figure 6**).

On the fungal side, the PTs of *R. irregularis* were expressed in the ERM and in the IRM of poplar and sorghum roots. The high affinity transporter *RiPT1* was previously found to be regulated in the ERM by external Pi concentration (Maldonado-Mendoza et al., 2001) and expressed at the arbuscular side (Fiorilli et al., 2013). Here, *RiPT1* was up-regulated by external low-P concentrations in the ERM but also in the IRM. High expression of *RiPT1* suggests that *RiPT1* is the main transporter for Pi uptake and symbiotic Pi transfer in our conditions. Maldonado-Mendoza et al. (2001) predicted the existence of other PTs operating at high external P concentrations. Indeed, *RiPT3* and *RiPT7* were expressed but not affected by the nutrient conditions in the ERM. It might be that higher Pi concentrations are needed to increase expression of these possible high affinity transporters. Clearly, *RiPT3* and *RiPT7* were induced in the IRM with sorghum, with a similar tendency in poplar, suggesting that they participate also in Pi transfer/exchanges in the symbiosis.

### P-Dependent Regulation of Phosphate Transporter Expression

Low-P conditions induced expression of *PtPT1.1*, *PtPT1.2*, *PtPT1.4*, and *PtPT1.11* in poplar roots, showing that expression and regulation of PTs is dependent on Pi availability (**Figure S3**). In the mycorrhizal microcosm, the only Pi source was the AM fungal symbiont, and the absence of a direct Pi source further increased expression of these four transporters. As we still observed a Pi-dependent regulation of transporter expression, our data suggest that these PTs are regulated by external and internal Pi-concentrations and are regulated independently by the mycorrhizal pathway. Increased expression of PTs in low-P condition further suggests that the AM fungus supplies the host plant with more Pi if the fungus itself has increased access to Pi. By regulating these PTs independently poplar ensures a P nutrition uncoupled from the AM symbiont. Symbiosis-independent P-nutrition is necessary for perennial plants as mycorrhizal abundance varies in nature with the seasons (Courty et al., 2008; Dumbrell et al., 2011).

The fact that *PtPT1.2*, *PtPT1.4*, and *PtPT1.11* expression was also induced upon P-limiting conditions in the shoots suggests that they function in Pi uptake at the root-soil interface as well as in intercellular distribution and translocation of Pi from root to shoot. *PtPT1.9* was mainly expressed in the shoot, which suggests that it is mainly responsible for Pi allocation in the shoots.

Sorghum on the other hand turned out to be more mycorrhiza-dependent than poplar. Under mycorrhization PTs were equally low expressed as under a non-mycorrhizal high-P condition, indicating that the AM fungus was able to cover the Pi needs of sorghum. Induction of PT in the shoots in the non-mycorrhizal low-P condition further showed that the plant suffered of P deficiency and therefore probably reallocated Pi from old leaves (source) to young leaves (sink). The stronger dependency of sorghum to the AM fungus was also indicated by Pi-accumulation in mycorrhizal and non-mycorrhizal sorghum (**Figure S2**).

### Symbiotic Nitrogen Exchange

As symbiotic N transfer is also an important aspect of AM symbiosis we analyzed the effects of mycorrhization and Pi availability on plant and AM fungal AMT expression (**Figure 6**). P-content has a stronger effect on poplar N contain than mycorrhization. Sorghum N nutrition is not affected by Pi availability. In poplar, mycorrhization induced expression of three AMTs. Our results are supported by previous studies which showed that *PtAMT1.1* and *PtAMT1.2* were mycorrhiza-inducible when poplar was mycorrhiza, suggesting a sensor role l with the ectomycorrhizal fungi *P. involutus* (Couturier et al., 2007) and *A. muscaria* (Selle et al., 2005) (**Figure S6**). In addition, we found that *AMT3.1* is as well a mycorrhiza-inducible transporter in the roots, which is in contrast to the data of Couturier et al. (2007) who detected *PtAMT3.1* solely in senescing leaves. In shoots, the expression of *PtAMT1.1* is mediated by Pi availability under mycorrhizal conditions. Further, increased expression of *PtAMT1.2* and *PtAMT3.1* suggests an increased ammonium transfer when the fungal needs of Pi are accomplished. Analysis of the transcriptome dataset revealed that *PtAMT4.1*, *PtAMT4.2*, and *PtAMT4.3* were also induced upon mycorrhization independently from the P supply of the fungus as it was the case in our previous study where mycorrhizal poplar was set under N stress (Calabrese et al., 2017).

As there is an ongoing debate on whether amino acids as an organic N source can be taken up by AM fungi and transferred from the fungus to the plant (reviewed in Hodge and Storer, 2015) we screened the transcriptome data of poplar and identified several amino acid transporters and H^+^/oligopeptide symporters that were either induced or repressed upon mycorrhization (**Table S3**). Specific induction of amino acid transporters and one of the H^+^/oligopeptide transporters indicate that amino acids are transferred from the AM fungus to the plant as an alternative N source. However, our metabolome analysis on mycorrhizal and non-mycorrhizal poplar roots showed that mycorrhization reduced the abundance of most metabolites including amino acids in the colonized roots tissue (**Tables 2** and **S7**), suggesting high rates of metabolic turnover by the fungus or the host roots or, alternatively, transport to the shoots. Interestingly, an accumulation of relevant metabolites might be detectable in the shoots as it was demonstrated by Whiteside et al. (2012) for the amino acids phenylalanine, lysine, asparagine, arginine, histidine, cysteine, methionine, and tryptophan with quantum dot analysis.

In the well-established mycorrhizal symbiosis described here, *GintAMT3* was significantly induced in the IRM in both hosts, poplar and sorghum (**Figure 4**), indicating a major participation of this transporter in the exchanges of ammonium at the arbuscule (Calabrese et al., 2017). High *GinAMT2* expression levels independent of Pi-supply and localization in the ERM and IRM indicate that it is a low affinity transporter for ammonium. Moreover, *GinAMT2* displays high sequence similarity to *GintAMT3* which is a low affinity transporter (Calabrese et al., 2017). *GintAMT1* on the other hand, a high affinity transporter, was expressed at low levels in the ERM and IRM independent of the P availability. Together, these findings indicate that GintAMT1 and *GintAMT2* are mainly involved in the uptake of ammonium in the ERM and in the IRM, and that GintAMT3 is mainly involved in the exchanges/competition of/for ammonium at the biotrophic interface between the AMF and its host plant.

In sorghum, only *SbAMT3.1* was induced in mycorrhizal roots (**Figure S7**). In contrast to Koegel et al. (2013)*SbAMT4* was not expressed in our experimental conditions. Induced expression of *SbAMT1.1* and *SbAMT1.2* in the low-P condition suggests that upon sensing of nutrient stress the plant activates a general nutrient uptake program to avoid running short on one or more essential nutrients. In addition our data show that regulation of sorghum AMTs is less mycorrhiza-dependent than nutrient-dependent. Under mycorrhization the plant is supplied with sufficient P and N to keep the P and N level constant within the plant. This scenario may explain the unchanged expression of the SbAMTs. Interestingly, mycorrhization induced expression of *SbAMT1.1* and *SbAMT1.2* in the shoots in low-P and of *SbAMT3.3* in high-P conditions. An increased translocation rate of ammonium from root to shoot upon mycorrhization may be a possible explanation. Moreover, *SbAMT1.1* and *SbAMT1.2* expression levels have a tendency to be also concentration dependent. Strong induction of *SbAMT3.3* in the shoots indicated an increased ammonium flux during high-P and mycorrhizal condition.

### Symbiotic Carbon Exchange

In order to shed some light on symbiotic carbon exchange we used qRT-PCR and mRNA-Seq analysis to identify possible transporters that enable carbohydrate transport from the plant to the fungus. Interestingly, quantitative RT-PCR expression analysis and mRNA-Seq analysis revealed downregulation of carbohydrate transporters in poplar (**Figure S8** and **Figure 6**) and sorghum (**Figure S9**), which might indicate that carbohydrates are actively sequestered by the fungus. But we also identified two carbohydrate transporters induced upon mycorrhization (**Table S3**). The UDP-galactose transporters are intracellular transporters situated in the cisternae of the Golgi lumen where UDP-galactose is used for synthesis of non-cellulosic polysaccharides and glycoproteins (Norambuena et al., 2002). Only recently, it was shown that they also transport rhamnose (Rautengarten et al., 2014). In the Golgi-network and the early endosome the newly synthesized proteins are sorted either for the secretion to the plasma membrane, the extracellular matrix or for degradation (Feraru et al., 2012; Brandizzi and Barlowe, 2013; McFarlane et al., 2014). Induced expression of UDP-galactose therefore indicates an increased transport activity of proteins to colonized cells which goes along with increased plasma membrane synthesis. In *M. truncatula*, it has been shown that arbuscule development goes along with the synthesis of the periarbuscular membrane which is distinct from the plasma membrane (Pumplin and Harrison, 2009). Further it has been shown that AM-induced transporters are specifically directed to the periarbuscular membrane and an involvement of the trans-Golgi for PT has been implicated (Pumplin et al., 2012). The other transporter induced upon mycorrhization is a SUT/spinster transmembrane protein, a member of the major facilitator proteins, which makes it a candidate for being localized at the cell membrane eventually stimulating carbohydrate transport to the AM symbiont.

Interesting is also that a glucose-6-phosphate/phosphate and phosphoenolpyruvate/phosphate antiporter was induced. Glucose-6 phosphate/phosphate antiporters are normally expressed in non-green plastids and serves as carbohydrate importer. In amyloplasts glucose-6 phosphate/phosphate antiporters are located at the site of starch and fatty acids synthesis (Kammerer et al., 1998; Flügge, 2001). Phosphoenolpyruvate/phosphate antiporter mediates the transport of the transport of Phosphoenolpyruvate into the organelles as in amyloplasts, for the synthesis of aromatic amino acids and several secondary compounds. Increased activity of this transporter suggests a decrease of freely available sugars, of storing starch and of aromatic amino acids synthesis. These results shed light the link between carbon, phosphorus, but also N.

In the AM fungi *G. pyriformis* and *R. irregularis* several carbohydrate transporters were identified. For GpMST1 and RiMST2, it was shown that they specifically transport monosaccharides. *RiMST2* was found to be expressed at the arbuscular site and in the intraradical hyphae (Schüßler et al., 2006; Helber et al., 2011). Helber et al. (2011) proposed a model in which the absorbed sugars might derive from cell wall degradation but this is contradictory to previous observations in which carbohydrate transfer varied with the source strength of the host plant (Olsson et al., 2010; Fellbaum et al., 2014; Zhang et al., 2015). We demonstrated that RiMST2 was induced in the IRM of its host plants poplar (**Figure 3**) and sorghum (**Figure 3**) and that its expression level remained unchanged by Pi availability. The metabolome analysis revealed further that mycorrhization significantly decreased the abundance of monosaccharides (i.e. glucose, rhamnose and ribose) in AM poplar roots. In AM fungi it has been shown that hexoses received from the host plants are transformed to glycogen, trehalose. Our observations are consistent with these findings that monosaccharides are taken up mainly by RiMST2 from the apoplast, converted into disaccharides (*i.e.* trehalose) for transport and hydrolyzed in the ERM into monosaccharides, ready for direct usage or for storage. The mycorrhizal-dependent downregulation of carbohydrate transporters and monosaccharide abundance in poplar roots, along with reduced RiMST2 expression at the arbuscular site and in the IRM suggests that the fungus actively sequesters the sugars on its own demand.

Taken together our data suggest that the plant strictly regulates carbohydrate allocation on mycorrhizal roots. With the Sugar transporter/spinster transmembrane protein a possible candidate for symbiotic carbon transfer from the plant to the periarbuscular space is found. The fungus may take up sugars from there by RiMST2.

AMF are fatty acid (FA) auxotrophs as their fatty acids are provided by their host plant (McLean et al., 2017). To date, mechanisms of FA transport are unknown. We observed that fungal lipid metabolism is highly expressed in the IRM. While many lipases, elongases, and desaturases are expressed both in the ERM and IRM, they might play a role in the storage and use of triglycerides in lipid droplets. However, their over-expression *in planta* could be a clue of the recovering process of FA from the plant.

## Conclusion

Here, we demonstrated that mycorrhization leads to specific induction of a variety of transporters. As P and N are key elements of mycorrhizal symbiosis, we tested the expression of PT and AMT and show that mycorrhization specifically induces expression of a selection of PT and AMTs in poplar and in sorghum. Further, we identified one carbohydrate transporter specifically induced in mycorrhizal root tissue that might be a possible candidate for symbiotic carbon exchange. By contrast, other carbohydrate transporters were down-regulated upon mycorrhization indicating that the plant may not volunteer provision of carbohydrates in exchange for mineral nutrients. We further showed that some nutrient transporters are more strongly expressed in the IRM compared to the ERM, which indicates that they are directly involved in nutrient transfer at the arbuscular membrane or, as in the case of MST, in nutrient uptake into the intraradical hyphae (**Figure 6**). Nevertheless, despite its importance, the release of major nutrients taken up by the ERM into the root apoplasm occurs through widely unknown mechanisms, which must include the differentiation and polarization of the fungal membrane transport functions. Transport processes across the polarized membrane interfaces are of major importance in the functioning of the established mycorrhizal association as the symbiotic relation is based on an apparent ‘fair-trade’ between fungus and host plant. PTs and AMTs are active transporters and they are able to transport nutrients against the direction of concentration gradients using electrochemical potential differences build up by proton-pumping ATPases. In fact, the reality is probably much more complex, with reuptake of nutrients at the biotrophic interface allowing both partners to, at least partially, control the exchanges (**Figure 6**).

In agriculture, vast amounts of mineral fertilizers are applied to the field to increase crop yield. Indeed, the amount of applied fertilizer might actually exceed the plant needs for mineral nutrients. Collecting data such as ours will help to further deepen our understanding of the plant-AM symbiosis and may lead to development of a more sustainable agriculture with a reduced fertilizer input and improved adaptation to changing environmental conditions.

## Data Availability Statement

Raw RNAseq data and global mapping data analyses were deposited at GEO (GSE138316).

## Author Contributions

SC made the major part of the experiments. AS and LC made in silico analysis. JK and AE performed metabolite analysis. All co-authors participated in writing.

## Funding

This project was supported by the Swiss National Science Foundation (grants no. PZ00P3_136651 to P-EC and no. 127563 to TB). The authors thank the following Institutions for financial support: the Burgundy Franche Comté Regional Council, the division of Plant Health and Environment of the French National Institute for Agricultural Research (INRA).

## Conflict of Interest

The authors declare that the research was conducted in the absence of any commercial or financial relationships that could be construed as a potential conflict of interest.

## Abbreviations

AM, Arbuscular mycorrhiza; AMF, Arbuscular mycorrhizal fungi; AMT, Ammonium transporter; ERM, Extraradical mycelium; IRM, Intraradical mycelium; N, Nitrogen; P, Phosphorus; Pi, inorganic phosphate; PT, Phosphate transporter; GS/GOGAT, glutamine synthetase/glutamine oxoglutarate aminotransferase.

## Supplementary Material

Table S1 Primer used for qRT-PCR in P. trichocarpa, S. bicolor and R. irregularis.

Table S2 Percentage of colonized P. trichocarpa and S. bicolor roots colonized by R. irregularis. Statistical analysis was performed on six biological replicates by T.TEST (p<0.05) for each plant species. Significant differences are indicated by lower case letters.

Table S3 Differentially expressed amino acid and carbohydrate transporters in P. trichocarpa. Significant p-values (p<0.05) are highlighted in bold.

Table S4 P. trichocarpa and S. bicolor transporters regulated by mycorrhizal symbiosis and P treatment.

Table S5 Name of fungal genes regulated by P treatment and during the interaction with P. trichocarpa and S. bicolor.

Table S6 Relative abundances of metabolites detected in the ERM of R. irregularis. Data was log transformed and tested by Wilcoxon rank sum test (p<0.05) in MeV v4.9 (http://www.tm4.org/mev.html). Significant p-values are highlighted in bold.

Table S7 Relative abundances of metabolites detected poplar roots. Abundances were measured in the mycorrhized (+AM) and non-mycorrhized (-AM) poplar roots under high (hP) and low (lP) P availability. Data was log transformed and tested by y 2-way analysis of variance (ANOVA) (p<0.05) in MeV v4.9 (http://www.tm4.org/mev.html). Significant p-values are highlighted in bold.
