# Supplementary material for: Imbalanced Regulation of Fungal Nutrient Transports According to Phosphate Availability in a Symbiocosm Formed by Poplar, Sorghum, and Rhizophagus irregularis
Source: Front Plant Sci. 2019 Dec 12;10:1617. doi: 10.3389/fpls.2019.01617 (PMC6920215; doi:10.3389/fpls.2019.01617)
Supplement: Table S1 — Primer used for qRT-PCR in P. trichocarpa, S. bicolor and R. irregularis. [file Table_1.pdf]

Name	Sequence (5′-> 3′)	Transcript ID		Reference
PtPht1.1f	GCGATTCACGAGGTTTTTCA	Potri.010G072000		Loth-Pereda *et al* . (2011)
PtPht1.1r	GGCGAAGAAAAAGGTCAACG			
PtPht1.2f	CACAGACCGAACGAAGACTG	Potri.010G071700		Loth-Pereda *et al* . (2011)
PtPht1.2r	ATCACACTGAAGCCATCCTAGC			
PtPht1.3f	ACCACCGAATTGGCTTCG	Potri.010G071500		Loth-Pereda *et al* . (2011)
PtPht1.3r	ATGGCAAGTAGACCTCAATCTCG			
PtPht1.4f	GATCTTCCCTGCAAGGTTAAGG	Potri.005G223500		Loth-Pereda *et al* . (2011)
PtPht1.4r	TCCTTGTGCGGGCTCTG			
PtPht1.5f	GGACCCAATGCCACCAC	Potri.002G038900		Loth-Pereda *et al* . (2011)
PtPht1.5r	CTTGGCCTGGTCCTGATTC			
PtPht1.6f	ATCGCCAGGGCTCAATTC	Potri.005G175500		Loth-Pereda *et al* . (2011)
PtPht1.6r	CTATTATTGCGCCAGCCTTC			
PtPht1.7f	CGAATTGGATTCGTGGTTATG	Potri.005G223600		Loth-Pereda *et al* . (2011)
PtPht1.7r	CCTTGTCCTGGTCCTGAGC			
PtPht1.8f	GATAGGATCGGAAGGTTCATAATC	Potri.019G061900		Loth-Pereda *et al* . (2011)
PtPht1.8r	GGTGACCACCACAGAAATCC			
PtPht1.9f	GATCACCTACAATTTCTTCTG	Potri.002G005500		
PtPht1.9r	GGTGAACTAGGAGTAATCAGATAC			
PtPht1.10f	TGCTAATGCTTGGTGCTTG	Potri.015G022800		Loth-Pereda *et al* . (2011)
PtPht1.10r	GATGAACATGGCACGAGATG			
PtPht1.11f	GCCACCCAAAGACTTCTGTC	Potri.005G256100		
PtPht1.11r	TTCAATCATCTGATAGGACACCA			
PtPht1.12f	GGAAACCATGTGGGAGTGC	Potri.001G318500		
PtPht1.12r	CTGGCCAGCTAAAGTTCCAC			
PtrAMT1.1f	TTCGCAAGCTCAAGCTC	Potri.010G063500		Selle *et al* . (2005)
PtrAMT1.1r	AATATGAGGTCCCTCTTAGACG			Couturier *et al* . (2007)
PtrAMT1.2f	GCCTGCGAAGAACACCTC	Potri.019G023600		
PtrAMT1.2r	GCATCAAACTTGATCACACATTG			Couturier *et al* . (2007)
PtrAMT3.1f	GCCGTGCATGGTGAAGAG	Potri.001G305400		
PtrAMT3.1r	TTGATGACTTGCGCTCCA			
PtrSUT1f	CCCACCAGTAGTAGTGC	Potri.013G115200		
PtrSUT1r	CCAGTCACTAGTCTTGGAAGG			
PtrSUT3f	CTGGTGCTGGCCAAGG	Potri.019G085800		
PtrSUT3r	GGCATCCCAAGGTCCAC			
PtrSUT4f	AGGCAGGGTGAGGAGGAT	Potri.002G106900		
PtrSUT4r	AGCGACACGACCTTCCAG			
PtrSUT5f	GCGGTGGCCAAGGATT	Potri.008G148100		
PtrSUT5r	GAACGAAGGCTGGTATA			
PtrSUT6f	GGTCTACAGTCAGAGAGATGGTTC	Potri.010G093600		
PtrSUT6r	AGCGTTCTGCGCGCTTCT			
PtUBI f	GCAGGGAAACAGTGAGGAAGG	Potri.015G013600		
PtUBI r	TGGACTCACGAGGACAG			
SbPht1.1f	GGCCAAGGTGCTCAAGAAG	Sobic.001G502000		Walder *et al* . (2015)
SbPht1.1b	GGAGGAACTGCACCGAGAAG			
SbPht1.2f	ACTAAGCAGCAGCCTCCGTA	Sobic.006G027300		Walder *et al* . (2015)
SbPht1.2b	AAGCCACAAGGAAACCATTG			
SbPht1.3f	TACTCGCGTATGAACATGCC	Sobic.001G513400		Walder *et al* . (2015)
SbPht1.3b	TCCTCCTTATTGCCGATGTC			
SbPht1.4f	GGCGCCGTCGTACCAGGACAA	Sobic.001G234900		Walder *et al* . (2015)
SbPht1.4b	GAGCGCCGCCGGGATGGT			
SbPht1.5f	GAGAATCTGGACGAGATCAC	Sobic.001G502100		Walder *et al* . (2015)
SbPht1.5b	CAGGTTCTGGCTGTAGTAGG			
SbPht1.6f	CAAGCTCGGCCGTAAGAAGG	Sobic.007G164400		Walder *et al* . (2015)
SbPht1.6b	GCCAGAAGCGGAAGAAGCAC			
SbPht1.7f	GGACACCAGCAAGGACAAC	Sobic.001G234800		Walder *et al* . (2015)
SbPht1.7b	CGCGATGGAGCAGATGAC			
SbPht1.8f	GCAGCGAGGCCAATGAGACT	Sobic.002G116100		Walder *et al* . (2015)
SbPht1.8b	TTGGCTCCGGTAGGAAGCAG			
SbPht1.9f	GAGGACGAGCCGTTCAAGAG	Sobic.006G026900		Walder *et al* . (2015)
SbPht1.9b	CGCGACGGAGAAGAAGTACC			
SbPht1.10f	CACCATGTGCTGGTTACTTC	Sobic.006G026800		Walder *et al* . (2015)
SbPht1.10b	GATAATCGCCTGAGTACGTG			
SbPht1.11f	CGTGGTTCCTTCTGGACATA	Sobic.003G243400		Walder *et al* . (2015)
SbPht1.11b	TCTCGAACACCTCCTTGAGT			
SbSUT1f	GTGCTCCTGTAATCTTTGTGTCC	Sb01g045720		
SbSUT1r	ACTATACTGCACATTGATTGATCG			
SbSUT2f	GCACATGCATTGAATGAACC	Sb04g038030		
SbSUT2r	TTCGCATTTGGAAATTCCTC			
SbSUT3f	GGCCGGATCAAACAAGAT	Sb01g022430		
SbSUT3r	GGCATTGCGAAGGAATGA			
SbSUT4f	CGATCCATGATGATGTCCAG	Sb08g023310		
SbSUT4r	GTTCCAGGCCTTGCTGTC			
SbSUT5f	CCCGTAGTGTTGCGGAGTC	Sb04g023860		
SbSUT5r	CCAATGGATCGGAAAATAAAG			
SbSUT6f	GCACAACAGCACAAAGAAGG	Sb07g028120		
SbSUT6r	AGGCAGAAGAGGCTGAGATG			
SbAMT1;1f	GCTGTGGTTCGGCTGGTA	Sb06g022230		Koegel *et al* . (2013)
SbAMT1;1r	GGACTTGAGGATGGTGGTGAA			
SbAMT1;2f	TCCATTGCTCCTCGTTGC	Sb09g023030		Koegel *et al* . (2013)
SbAMT1;2r	GGCTTTGCTCCCTCTTCC			
SbAMT2;1f	TCCCGCCCGCCTACAGCT	Sb09g023030		Koegel *et al* . (2013)
SbAMT2;1r	GTCACCATTCAGCTGTAG			
SbAMT2;2f	GCGGCTTCCTCTACCAGTG	Sb03g038840		Koegel *et al* . (2013)
SbAMT2;2r	CCTCTCCCTGTCGCTCTTC			
SbAMT3;1f	GGCCTCGTCTGCATCACT	Sb03g041140		Koegel *et al* . (2013)
SbAMT3;1r	GGGTGTCGTCCACTTGCT			
SbAMT3;2f	CCGCACGCACTCTATCTGTA	Sb01g001970		Koegel *et al* . (2013)
SbAMT3;2r	TCGCTGCTTATTGGGGTTAG			
SbAMT3.3f	CGTCATTGCCTGGAACATC	Sb04g022390		Koegel *et al* . (2013)
SbAMT3.3r	AGCATCATCCCCGATAAGC			
SbAMT4f	CGAACAACATTCTCCTGACG	Sb01g008060		Koegel *et al* . (2013)
SbAMT4r	CCCGAACACGAAGCAGTC			
SbUBIf	CAAGGAGTGCCCCAACAC	Sb10g026870		Koegel *et al* . (2013)
SbUbir	TGGTAGGCGGGTAAAGCAAA			
		**Transcript ID**	**Protein ID**	
GintAMT1f	TGTGTCAGCATTGTCTTCAGT	337137	337025	López-Pedrosa *et al* . (2006)
GintAMT1r	GGCAAGTGCGGGTGTAATAG			
GintAMT2f	GTGCCAATGCCGCTAACA	314321	314209	Pérez-Tienda *et al* . (2011)
GintAMT2r	GCCAGAACAGAATCCCAAAG			
GintAMT3f	GGG CTT GAC TTT GCT GGT	218287	218175	
GintAMT3r	TTC GTC CCT TCC ATG ACC			
RiPT1f	ATGGGTTTCGCTGTCCTCAC	345640	345528	
RiPT1r	CCCCTGGAACGATGAATG			
RiPT3	CGGCGGTGATTATCCTCT	7378	7266	
RiPT3	GCCGCCATCATTGCTC			
RiPT5f	GGCGCGAATACGTCAGAA	346370	346258	
RiPT5r	GCTGCAACACCAACACCA			
RiPT7	GATCCTTGGACTGGAACAC	67530	67418	
RiPT7r	GCGATGACTCCCATATCACC			
RiMST2f	GGCAGGATATTTGTCTGATAG	341721	341609	Helber *et al* . (2011)
RiMST2r	GCAATAACTCTTCCCGTATAC			
RiMST3f	ATTCTCGATTCTTGGTGCATC	34584	34472	Helber *et al.* (2011)
RiMST3r	ATACGCCAGCAACGACTC			
RiMST4f	TAGCTACATTTGCTATTGGTTTAG	155358	155246	Helber *et al.* (2011)
RiMST4r	CCCTAACTTCCAAAAATAATGAAC			
RiTEFf	TGACAGGCGATCTGGTAAGG			
RiTEF	TCAGCGAAGGTCTCAACCAC			
