# Supplementary material for: Imbalanced Regulation of Fungal Nutrient Transports According to Phosphate Availability in a Symbiocosm Formed by Poplar, Sorghum, and Rhizophagus irregularis
Source: Front Plant Sci. 2019 Dec 12;10:1617. doi: 10.3389/fpls.2019.01617 (PMC6920215; doi:10.3389/fpls.2019.01617)
Supplement: Table S2 — Percentage of colonized P. trichocarpa and S. bicolor roots colonized by R. irregularis. Statistical analysis was performed on six biological replicates by T.TEST (p<0.05) for each plant species. Significant differences are indicated by lower case letters. [file Table_2.pdf]

Plant	Treatment	Hyphae (%)		Arbuscule (%)		Vesicle (%)	
P. trichocarpa	low Pi	79[a]	± 10	34[a]	± 8	10[a]	± 8
	high Pi	87[a]	± 6	34[a]	± 8	9[a]	± 4
S. bicolor	low Pi	94[a]	± 5	16[a]	± 7	37[a]	± 11
	high Pi	93[a]	± 5	5[b]	± 5	43[a]	± 17
