# Supplementary material for: Imbalanced Regulation of Fungal Nutrient Transports According to Phosphate Availability in a Symbiocosm Formed by Poplar, Sorghum, and Rhizophagus irregularis
Source: Front Plant Sci. 2019 Dec 12;10:1617. doi: 10.3389/fpls.2019.01617 (PMC6920215; doi:10.3389/fpls.2019.01617)
Supplement: Table S3 — Differentially expressed amino acid and carbohydrate transporters in P. trichocarpa. Significant p-values (p<0.05) are highlighted in bold. [file Table_3.pdf]

Gene ID	kogid	kogdefline	kogClass	kogGroup	TAIR10	TAIR10_defline	Mean number of reads						log2 fold change							padj						
							+AM	-AM	lP +AM	hP +AM	lP -AM	hP -AM	+AM vs -AM	+P-Gi vs -P-Gi	+P-Gi vs +P+Gi	+P+Gi vs -P-Gi	+P+Gi vs -P+Gi	-P+Gi vs +P-Gi	-P+Gi vs -P-Gi	+AM vs -AM	+P-Gi vs -P-Gi	+P-Gi vs +P+Gi	+P+Gi vs -P-Gi	+P+Gi vs -P+Gi	-P+Gi vs +P-Gi	-P+Gi vs -P-Gi
Potri.004G111400	KOG1305	Amino acid transporter protein	Amino acid transport and metabolism	METABOLISM	At3g30390	Transmembrane amino acid transporter family prote	9132,2	4260,3	7247,8	10175,9	4790,7	4131,3	-1,1	0,2	1,3	1,1	-0,4	0,9	0,7	**0,000**	0,916	**0,001**	**0,002**	0,615	**0,016**	**0,047**
Potri.001G007300	KOG1287	Amino acid transporters	Amino acid transport and metabolism	METABOLISM	At3g13620	Amino acid permease family protein	2422,2	1284,3	2132,1	2488,3	1372,6	1315,6	-0,9	0,0	0,9	1,0	-0,2	0,7	0,8	**0,002**	0,991	0,096	**0,000**	0,803	0,241	**0,008**
Potri.001G335200	KOG1303	Amino acid transporters	Amino acid transport and metabolism	METABOLISM	At5g40780	lysine histidine transporter 1	174,8	23,3	130,6	202,4	25,0	23,4	-2,6	0,0	2,5	2,8	-0,4	2,0	2,1	**0,000**	0,990	**0,000**	**0,000**	0,731	**0,006**	**0,000**
Potri.001G335300	KOG1303	Amino acid transporters	Amino acid transport and metabolism	METABOLISM	At5g40780	lysine histidine transporter 1	12148,7	5498,0	13220,6	9941,8	5871,3	5636,1	-1,1	0,0	0,9	0,8	0,4	1,2	1,2	**0,000**	0,990	**0,017**	**0,023**	0,682	**0,007**	**0,006**
Potri.002G078300	KOG1289	Amino acid transporters	Amino acid transport and metabolism	METABOLISM	At2g01170	bidirectional amino acid transporter 1	301,7	102,0	311,0	264,1	134,6	79,1	-1,5	0,5	1,6	1,1	0,2	1,8	1,2	**0,000**	0,646	**0,001**	**0,000**	0,869	**0,002**	**0,006**
Potri.002G079500	KOG1303	Amino acid transporters	Amino acid transport and metabolism	METABOLISM	At1g77380	amino acid permease 3	1037,3	130,3	1294,1	682,4	182,0	91,6	-2,5	0,7	2,5	1,8	0,5	3,0	2,3	**0,000**	0,553	**0,000**	**0,000**	NA	**0,000**	**0,000**
Potri.003G138100	KOG1303	Amino acid transporters	Amino acid transport and metabolism	METABOLISM	At5g41800	Transmembrane amino acid transporter family prote	721,7	294,8	603,1	773,9	427,4	191,9	-1,2	0,8	1,9	0,9	-0,3	1,6	0,6	**0,002**	0,278	**0,000**	**0,020**	0,795	**0,002**	0,284
Potri.008G036300	KOG1303	Amino acid transporters	Amino acid transport and metabolism	METABOLISM	At2g39130	Transmembrane amino acid transporter family prote	165,8	471,0	203,8	112,3	626,3	362,2	1,4	0,6	-1,4	-2,1	0,7	-0,6	-1,4	**0,000**	0,588	**0,014**	**0,000**	0,336	0,294	**0,000**
Potri.010G221900	KOG1303	Amino acid transporters	Amino acid transport and metabolism	METABOLISM	At5g19500	Tryptophan/tyrosine permease	112,2	34,3	110,0	103,8	39,7	32,6	-1,6	0,2	1,6	1,4	0,1	1,7	1,5	**0,000**	0,920	**0,000**	**0,000**	0,956	**0,003**	**0,002**
Potri.010G226000	KOG1303	Amino acid transporters	Amino acid transport and metabolism	METABOLISM	At2g39130	Transmembrane amino acid transporter family prote	3799,5	1519,8	2992,1	4257,2	1615,2	1565,4	-1,3	0,0	1,4	1,5	-0,4	1,0	1,0	**0,000**	0,995	**0,001**	**0,000**	0,569	0,072	**0,004**
Potri.013G030900	KOG1286	Amino acid transporters	Amino acid transport and metabolism	METABOLISM	At4g21120	amino acid transporter 1	816,5	187,7	658,0	899,8	200,9	192,1	-2,0	0,0	2,2	2,2	-0,3	1,6	1,6	**0,000**	0,985	**0,000**	**0,000**	0,795	**0,005**	**0,002**
Potri.002G258700	KOG1237	H+/oligopeptide symporter	Amino acid transport and metabolism	METABOLISM	At5g46050	peptide transporter 3	2477,2	543,7	1762,0	2965,7	790,6	351,7	-1,8	0,5	2,4	1,8	-0,5	1,8	1,1	**0,000**	0,715	**0,000**	**0,000**	0,579	**0,017**	0,065
Potri.002G258900	KOG1237	H+/oligopeptide symporter	Amino acid transport and metabolism	METABOLISM	At5g46050	peptide transporter 3	77,5	1,2	71,8	76,1	1,1	1,3	-4,3	0,0	3,4	3,3	0,0	3,7	3,8	**0,000**	NA	NA	NA	0,983	**0,000**	**0,000**
Potri.005G233500	KOG1237	H+/oligopeptide symporter	Amino acid transport and metabolism	METABOLISM	At3g54140	peptide transporter 1	702,2	11,7	724,8	614,6	11,4	12,8	-5,1	-0,1	4,1	4,5	0,1	4,8	5,2	**0,000**	0,968	**0,000**	**0,000**	0,922	**0,000**	**0,000**
Potri.010G068100	KOG1237	H+/oligopeptide symporter	Amino acid transport and metabolism	METABOLISM	At5g46050	peptide transporter 3	1367,5	371,7	1243,4	1364,7	322,6	453,6	-1,8	-0,4	1,6	2,1	-0,1	1,4	1,9	**0,000**	0,773	**0,000**	**0,000**	0,944	**0,022**	**0,000**
Potri.001G286600	KOG1330	Sugar transporter/spinster transmembrane	Carbohydrate transport and metabolism	METABOLISM	At5g64500	Major facilitator superfamily protein	1303,7	636,3	1483,6	1001,3	726,2	606,4	-1,0	0,2	0,8	0,6	0,5	1,3	1,1	**0,000**	0,877	**0,040**	0,076	0,540	**0,002**	**0,003**
Potri.001G111000	KOG1582	UDP-galactose transporter related protein	Carbohydrate transport and metabolism	METABOLISM	At4g23010	UDP-galactose transporter 2	1253,2	549,0	1435,1	953,9	587,1	562,1	-1,1	0,0	0,8	0,8	0,5	1,4	1,4	**0,000**	0,984	**0,022**	**0,001**	0,496	**0,002**	**0,000**
Potri.010G194100	KOG4852	Glucose-6-phosphate/phosphate and phosp	Carbohydrate transport and metabolism ;Amino a	METABOLISM	At3g11320	Nucleotide-sugar transporter family protein	5671,2	3045,8	6615,1	4195,6	3722,3	2662,1	-0,8	0,4	0,7	0,3	0,5	1,2	0,8	**0,040**	0,789	**0,016**	0,505	NA	0,056	0,195
Potri.002G112100	KOG1303	Amino acid transporters	Amino acid transport and metabolism	METABOLISM	At5g49630	amino acid permease 6	271,7	935,0	293,8	224,7	733,3	1218,3	1,7	-0,5	-2,0	-1,4	0,3	-1,7	-1,1	**0,000**	0,721	**0,000**	**0,000**	0,685	**0,002**	**0,007**
Potri.002G137000	KOG1286	Amino acid transporters	Amino acid transport and metabolism	METABOLISM	At1g05940	cationic amino acid transporter 9	1061,3	1601,5	896,2	1128,0	1699,1	1652,4	0,6	0,0	-0,4	-0,4	-0,3	-0,7	-0,7	**0,000**	0,992	0,077	**0,004**	0,707	0,066	**0,019**
Potri.004G172800	KOG1303	Amino acid transporters	Amino acid transport and metabolism	METABOLISM	At2g21050	like AUXIN RESISTANT 2	134,0	348,0	120,8	134,6	341,3	386,3	1,3	-0,2	-1,3	-1,1	-0,1	-1,4	-1,2	**0,000**	0,905	**0,001**	**0,003**	0,936	**0,009**	**0,011**
Potri.008G017100	KOG1286	Amino acid transporters	Amino acid transport and metabolism	METABOLISM	At5g04770	cationic amino acid transporter 6	47,7	149,5	37,8	52,6	117,9	193,9	1,5	-0,4	-1,4	-1,0	-0,3	-1,8	-1,4	**0,000**	0,788	**0,038**	**0,005**	0,754	**0,007**	**0,000**
Potri.008G066400	KOG1303	Amino acid transporters	Amino acid transport and metabolism	METABOLISM	At2g38120	Transmembrane amino acid transporter family prote	83,3	158,0	52,8	106,4	185,3	145,8	0,9	0,2	-0,3	-0,6	-0,8	-1,2	-1,5	**0,015**	0,867	0,567	0,109	0,181	**0,019**	**0,000**
Potri.010G191000	KOG1303	Amino acid transporters	Amino acid transport and metabolism	METABOLISM	At2g38120	Transmembrane amino acid transporter family prote	706,8	1135,0	616,2	732,0	1111,8	1261,8	0,7	-0,2	-0,6	-0,4	-0,2	-0,9	-0,7	**0,003**	0,784	0,124	0,223	0,850	**0,034**	0,057
Potri.014G036500	KOG1303	Amino acid transporters	Amino acid transport and metabolism	METABOLISM	At1g47670	Transmembrane amino acid transporter family prote	453,8	1105,5	330,0	536,3	1188,4	1125,6	1,2	0,0	-0,9	-1,0	-0,6	-1,5	-1,6	**0,000**	0,983	0,071	**0,000**	0,169	**0,002**	**0,000**
Potri.017G083700	KOG1303	Amino acid transporters	Amino acid transport and metabolism	METABOLISM	At5g15240	Transmembrane amino acid transporter family prote	267,5	1935,0	337,6	172,2	1320,1	2714,1	2,6	-0,8	-3,5	-2,6	0,7	-2,5	-1,6	**0,000**	0,348	**0,000**	**0,000**	0,328	**0,000**	**0,001**
Potri.019G039600	KOG1286	Amino acid transporters	Amino acid transport and metabolism	METABOLISM	At3g03720	cationic amino acid transporter 4	867,3	3767,5	834,4	819,7	3385,0	4487,4	2,1	-0,4	-2,2	-1,8	0,0	-2,2	-1,8	**0,000**	0,758	**0,000**	**0,000**	0,980	**0,000**	**0,000**
Potri.003G088800	KOG1237	H+/oligopeptide symporter	Amino acid transport and metabolism	METABOLISM	At1g32450	nitrate transporter 1.5	66,5	318,2	31,0	96,2	350,9	314,9	1,8	0,1	-1,1	-1,4	-1,0	-2,3	-2,9	**0,001**	0,980	0,218	**0,004**	0,148	NA	**0,000**
Potri.014G179400	KOG1237	H+/oligopeptide symporter	Amino acid transport and metabolism	METABOLISM	At1g32450	nitrate transporter 1.5	406,7	2599,7	316,6	459,7	2332,6	3099,9	2,1	-0,2	-1,5	-1,5	-0,3	-2,2	-2,3	**0,000**	0,928	0,072	**0,017**	0,845	NA	**0,000**
Potri.006G015200	KOG0569	Permease of the major facilitator superfami	Carbohydrate transport and metabolism	METABOLISM	At4g16480	inositol transporter 4	402,7	1750,0	404,2	363,6	1577,0	2080,1	2,1	-0,4	-2,3	-1,9	0,1	-2,1	-1,7	**0,000**	0,378	**0,000**	**0,000**	0,871	**0,000**	**0,000**
Potri.015G008100	KOG4332	Predicted sugar transporter	Carbohydrate transport and metabolism	METABOLISM	At4g27720	Major facilitator superfamily protein	726,7	1320,5	560,2	826,4	1401,2	1362,3	0,8	0,0	-0,6	-0,6	-0,5	-1,1	-1,1	**0,003**	0,996	0,260	**0,042**	0,537	**0,033**	**0,001**
Potri.005G075600	KOG2234	Predicted UDP-galactose transporter	Carbohydrate transport and metabolism	METABOLISM	At5g65000	Nucleotide-sugar transporter family protein	491,3	969,8	424,2	512,8	991,2	1037,7	1,0	-0,1	-0,9	-0,8	-0,2	-1,1	-1,0	**0,000**	0,953	**0,017**	**0,003**	0,796	**0,007**	**0,001**
Potri.010G180500	KOG2234	Predicted UDP-galactose transporter	Carbohydrate transport and metabolism	METABOLISM	At3g59360	UDP-galactose transporter 6	209,2	345,3	174,3	224,9	348,6	374,1	0,7	-0,1	-0,6	-0,5	-0,3	-0,9	-0,8	**0,000**	0,905	**0,003**	**0,034**	0,697	**0,015**	**0,023**
