# Supplementary material for: Imbalanced Regulation of Fungal Nutrient Transports According to Phosphate Availability in a Symbiocosm Formed by Poplar, Sorghum, and Rhizophagus irregularis
Source: Front Plant Sci. 2019 Dec 12;10:1617. doi: 10.3389/fpls.2019.01617 (PMC6920215; doi:10.3389/fpls.2019.01617)
Supplement: Table S4 — P. trichocarpa and S. bicolor transporters regulated by mycorrhizal symbiosis and P treatment. [file Table_4.pdf]

Name	Impact of mycorrhization (Myc+ LP VS. Myc- LP)		Impact of Phosphate (Myc+ HP VS. Myc+ LP)	
	Baggerley Fold Change	Baggerley FDR	Baggerley Fold Change	Baggerley FDR
Sobic.001G020350.1	1,690	0,056	1,955	0,002
Sobic.001G022700.1	10000,000	0,024	-1,348	1,000
Sobic.001G032000.1	-7,187	0,094	-1,246	0,959
Sobic.001G032200.1	3,308	0,000	-1,116	0,105
Sobic.001G059100.1	-1,709	0,000	-1,284	1,000
Sobic.001G071800.2	2,396	0,000	-1,323	0,000
Sobic.001G082700.1	13,460	0,002	1,137	1,000
Sobic.001G089400.1	26,619	0,051	1,172	1,000
Sobic.001G099300.1	10000,000	0,016	1,132	1,000
Sobic.001G099400.1	5,237	0,006	1,204	1,000
Sobic.001G099700.1	1,000	1,000	1,000	1,000
Sobic.001G106800.2	-7,208	0,382	4,646	0,551
Sobic.001G116400.2	-3,174	0,019	-2,101	0,456
Sobic.001G116500.1	1,609	0,000	-1,467	0,000
Sobic.001G120900.1	-2,045	0,004	1,136	0,879
Sobic.001G121600.1	1,000	1,000	1,000	1,000
Sobic.001G123600.1	-1,382	0,782	-1,051	1,000
Sobic.001G140000.1	-2,146	0,000	1,083	1,000
Sobic.001G142800.2	14,855	0,000	1,341	0,959
Sobic.001G142900.1	10000,000	0,000	-1,368	1,000
Sobic.001G149400.1	1,180	1,000	-1,141	1,000
Sobic.001G153100.1	18,825	0,000	1,199	1,000
Sobic.001G156800.2	-1,610	0,709	1,348	1,000
Sobic.001G183700.1	13,756	0,000	1,208	1,000
Sobic.001G184000.1	2,200	0,015	1,207	1,000
Sobic.001G184100.1	-1,094	1,000	-2,157	1,000
Sobic.001G184300.1	-1,817	0,443	-1,306	1,000
Sobic.001G187300.1	1,719	0,168	-1,923	0,196
Sobic.001G190200.1	-2,951	0,212	-1,008	1,000
Sobic.001G195800.1	-2,226	0,001	1,594	1,000
Sobic.001G208500.1	-10000,000	0,502	1,000	1,000
Sobic.001G217500.1	-2,227	0,013	1,260	0,690
Sobic.001G218900.1	1,000	1,000	1,000	1,000
Sobic.001G223100.2	1,133	1,000	1,237	0,952
Sobic.001G223200.1	-14,566	0,000	1,366	1,000
Sobic.001G233400.2	-1,548	0,205	1,324	0,962
Sobic.001G234800.1	6,090	0,000	-1,614	0,000
Sobic.001G234900.1	13,439	0,000	-2,038	0,000
Sobic.001G244500.2	1,000	1,000	1,000	1,000
Sobic.001G250200.1	5,164	0,003	-1,065	1,000
Sobic.001G254000.1	-1,970	0,931	-1,193	1,000
Sobic.001G261509.1	-7,561	0,039	2,570	0,624
Sobic.001G267100.1	-4,140	0,261	1,130	1,000
Sobic.001G275500.2	-16,885	0,000	1,530	0,959
Sobic.001G275800.1	1,000	1,000	10000,000	0,959
Sobic.001G276100.1	2,580	0,541	-1,716	1,000
Sobic.001G276400.1	1,000	1,000	1,000	1,000
Sobic.001G276600.1	8,292	0,031	-1,015	1,000
Sobic.001G276700.1	-7,179	0,598	-10000,000	0,658
Sobic.001G276800.1	-2,086	0,555	-2,335	0,973
Sobic.001G282000.1	-5,895	0,000	1,394	0,289
Sobic.001G282100.1	1,038	0,335	1,060	1,000
Sobic.001G282600.1	2,456	0,000	1,025	1,000
Sobic.001G282700.1	-1,569	0,000	-1,045	0,973
Sobic.001G288500.1	10000,000	0,018	-1,864	0,959
Sobic.001G297600.2	-1,136	0,097	-1,318	0,001
Sobic.001G307700.1	-17,580	0,000	1,860	0,548
Sobic.001G312900.1	3,756	0,000	1,079	1,000
Sobic.001G323600.1	-4,059	0,402	4,550	0,959
Sobic.001G339600.1	-1,739	0,000	-1,281	0,775
Sobic.001G344500.2	10,501	0,000	-1,248	0,000
Sobic.001G353200.1	-18,715	0,000	2,026	0,527
Sobic.001G359200.1	-1,661	0,062	-1,263	0,585
Sobic.001G359300.1	-11,650	0,127	1,915	0,959
Sobic.001G359400.1	1,180	0,384	1,000	1,000
Sobic.001G359500.2	-1,336	1,000	-1,406	1,000
Sobic.001G362500.1	1,971	0,421	1,326	1,000
Sobic.001G362601.1	1,999	0,002	1,142	1,000
Sobic.001G373600.1	-8,337	0,202	2,227	0,959
Sobic.001G374000.1	-1,058	0,813	-1,106	0,959
Sobic.001G377600.1	-5,060	0,395	-2,136	1,000
Sobic.001G378300.1	1,066	1,000	-1,647	0,551
Sobic.001G379900.3	8,283	0,000	-1,072	1,000
Sobic.001G383400.1	-2,031	0,360	1,591	0,982
Sobic.001G389900.1	-1,802	0,000	1,101	0,934
Sobic.001G406800.1	1,482	0,309	-1,003	1,000
Sobic.001G409900.1	3,565	0,000	1,081	1,000
Sobic.001G415300.1	3,584	0,000	-1,005	1,000
Sobic.001G428600.2	1,024	1,000	1,221	1,000
Sobic.001G439000.1	-3,735	0,000	-1,027	1,000
Sobic.001G444000.1	-3,681	0,352	1,338	0,959
Sobic.001G450000.1	2,588	0,000	-1,003	1,000
Sobic.001G451500.1	1,239	0,834	2,154	0,000
Sobic.001G454100.1	1,178	0,907	1,231	1,000
Sobic.001G454900.1	-2,598	0,000	-1,007	1,000
Sobic.001G465100.1	-10,846	0,000	1,363	1,000
Sobic.001G469500.1	-1,939	0,550	1,272	1,000
Sobic.001G469600.1	-20,479	0,000	1,416	0,781
Sobic.001G470600.1	1,280	0,690	1,831	0,279
Sobic.001G470800.1	-9,393	0,184	-1,082	1,000
Sobic.001G470900.3	-2,084	0,000	1,241	0,959
Sobic.001G484400.1	1,587	0,010	1,008	1,000
Sobic.001G488700.1	-4,557	0,013	-1,026	1,000
Sobic.001G493300.1	1,000	1,000	1,000	1,000
Sobic.001G496800.1	-3,832	0,000	1,394	0,000
Sobic.001G502000.1	7,967	0,000	-1,351	0,261
Sobic.001G502100.1	-2,648	0,738	-2,140	1,000
Sobic.001G504000.9	-4,562	0,000	1,024	1,000
Sobic.001G505100.1	-2,380	0,240	1,139	1,000
Sobic.001G511000.2	20,715	0,003	-1,014	1,000
Sobic.001G513400.1	10000,000	0,001	-10000,000	0,006
Sobic.001G535900.2	1,000	1,000	1,000	1,000
Sobic.001G539200.1	4,671	0,000	1,757	0,254
Sobic.001G541900.2	4,679	0,003	-2,343	0,223
Sobic.001G543500.1	-7,005	0,000	-1,081	1,000
Sobic.001G543900.1	3,988	0,029	-1,328	1,000
Sobic.002G001800.1	2,276	0,227	1,330	0,990
Sobic.002G004900.2	2,384	0,000	1,281	0,000
Sobic.002G034500.1	4,327	0,002	1,204	1,000
Sobic.002G035200.2	-7,200	0,040	1,040	1,000
Sobic.002G049700.1	10000,000	0,007	-1,776	0,959
Sobic.002G073600.1	-26,556	0,181	1,512	1,000
Sobic.002G083000.1	-2,255	0,001	1,126	1,000
Sobic.002G083100.1	-21,012	0,475	4,181	0,722
Sobic.002G083200.2	-5,186	0,000	-1,705	0,094
Sobic.002G091800.1	10000,000	0,181	-1,984	1,000
Sobic.002G108900.1	10000,000	0,502	-10000,000	0,959
Sobic.002G116100.1	23,575	0,055	-2,274	0,903
Sobic.002G121800.1	1,000	1,000	1,000	1,000
Sobic.002G124700.1	1,013	1,000	-1,001	1,000
Sobic.002G125000.1	1,326	0,993	-1,780	0,959
Sobic.002G125200.1	1,000	1,000	1,000	1,000
Sobic.002G125300.1	1,100	1,000	-2,388	0,242
Sobic.002G125500.1	-10000,000	0,502	10000,000	0,959
Sobic.002G125700.2	1,465	0,464	-1,454	0,849
Sobic.002G125800.1	2,561	0,000	-1,107	0,999
Sobic.002G130800.1	1,768	0,000	-1,353	0,007
Sobic.002G143500.3	-1,320	0,000	-1,025	1,000
Sobic.002G156000.1	3,528	0,000	-1,148	0,033
Sobic.002G156400.1	1,000	1,000	10000,000	0,570
Sobic.002G159900.2	1,787	0,012	-1,159	0,959
Sobic.002G188600.1	1,180	0,050	1,107	1,000
Sobic.002G193800.1	1,153	0,916	1,003	1,000
Sobic.002G197000.1	-10,374	0,000	-1,741	0,968
Sobic.002G201900.1	-2,410	0,000	1,133	0,896
Sobic.002G215800.1	-34,495	0,003	1,892	0,959
Sobic.002G218200.1	1,834	0,304	-1,104	1,000
Sobic.002G230600.1	2,877	0,000	-1,081	0,959
Sobic.002G233600.1	1,152	0,821	1,005	1,000
Sobic.002G243400.1	1,596	1,000	-10000,000	0,959
Sobic.002G256900.1	-1,008	1,000	1,451	0,959
Sobic.002G257001.1	-81,189	0,086	-1,186	1,000
Sobic.002G259301.1	1,000	1,000	1,000	1,000
Sobic.002G281000.1	-4,360	0,054	-1,036	1,000
Sobic.002G286800.1	1,186	0,835	-1,152	1,000
Sobic.002G313900.1	-1,420	0,014	-1,117	0,297
Sobic.002G322000.2	169,466	0,456	-12,525	0,959
Sobic.002G338500.1	-8,010	0,409	3,151	0,959
Sobic.002G353400.1	1,342	0,059	-1,191	0,959
Sobic.002G353900.1	-1,703	0,454	1,792	0,009
Sobic.002G354000.1	-2,901	0,117	1,397	1,000
Sobic.002G354100.2	-2,426	0,504	1,299	1,000
Sobic.002G354200.1	-3,840	0,270	1,633	0,357
Sobic.002G359400.1	-2,221	0,008	1,037	1,000
Sobic.002G367700.1	-1,040	0,965	1,053	1,000
Sobic.002G372100.1	-4,457	0,000	-1,274	1,000
Sobic.002G388800.1	-3,468	0,018	1,735	0,959
Sobic.002G388900.1	-5,328	0,392	1,614	1,000
Sobic.002G389000.1	-13,352	0,395	2,159	1,000
Sobic.002G389100.1	-1,261	0,942	1,035	1,000
Sobic.002G398600.1	-1,916	0,221	1,147	1,000
Sobic.002G402700.1	3,128	0,000	1,476	0,646
Sobic.002G408100.1	2,124	0,502	-1,735	1,000
Sobic.002G411500.1	1,096	0,965	1,312	0,331
Sobic.002G417500.1	1,048	1,000	-1,274	1,000
Sobic.002G419600.2	-20,261	0,000	1,607	0,967
Sobic.002G431000.1	-1,265	0,000	1,302	0,949
Sobic.003G002300.1	1,127	1,000	-1,722	1,000
Sobic.003G002900.1	-29,859	0,060	6,813	0,004
Sobic.003G006600.1	-10000,000	0,502	1,000	1,000
Sobic.003G007200.1	-10000,000	0,000	10000,000	0,959
Sobic.003G015200.1	-3,189	0,000	-1,201	1,000
Sobic.003G026400.1	1,493	0,147	1,246	0,992
Sobic.003G035500.1	-1,343	0,000	1,012	1,000
Sobic.003G038700.1	1,000	1,000	1,000	1,000
Sobic.003G038800.2	1,000	1,000	1,000	1,000
Sobic.003G047900.1	9,229	0,000	-1,119	0,790
Sobic.003G048200.1	-4,649	0,000	1,647	0,959
Sobic.003G050800.1	-4,183	0,000	1,423	0,842
Sobic.003G055500.1	-1,188	0,000	1,123	1,000
Sobic.003G084000.1	2,055	0,000	-1,028	1,000
Sobic.003G098100.1	-44,510	0,000	-1,189	1,000
Sobic.003G107100.1	-9,058	0,000	1,004	1,000
Sobic.003G109832.1	-15,406	0,002	-1,130	1,000
Sobic.003G110800.2	1,436	0,000	-1,045	1,000
Sobic.003G111000.1	-10,572	0,000	-1,193	1,000
Sobic.003G139300.3	-1,587	0,000	1,540	0,623
Sobic.003G145800.1	-2,564	0,000	1,024	1,000
Sobic.003G149000.1	-1,039	1,000	1,043	1,000
Sobic.003G149300.1	-1,158	0,222	1,514	0,000
Sobic.003G156200.1	1,000	1,000	10000,000	0,959
Sobic.003G164400.1	1,000	1,000	1,000	1,000
Sobic.003G180400.1	24,887	0,000	1,057	1,000
Sobic.003G181600.1	-1,526	0,000	1,440	0,000
Sobic.003G182800.2	4,692	0,000	-1,131	1,000
Sobic.003G188400.1	1,002	1,000	1,069	1,000
Sobic.003G192200.1	-5,330	0,317	2,460	0,959
Sobic.003G213000.1	34,038	0,000	-2,921	0,002
Sobic.003G215600.1	10000,000	0,002	-11,712	0,039
Sobic.003G216000.1	10000,000	0,005	-3,003	0,688
Sobic.003G223500.1	-2,287	0,027	-1,214	0,337
Sobic.003G227700.1	2,839	0,001	1,081	1,000
Sobic.003G227800.1	1,000	1,000	1,000	1,000
Sobic.003G227900.1	1,027	1,000	1,190	0,959
Sobic.003G228000.1	1,349	0,014	-1,115	0,959
Sobic.003G228100.1	2,888	0,000	-1,261	0,446
Sobic.003G228200.1	-5,149	0,482	1,113	1,000
Sobic.003G228300.1	9,294	0,000	1,229	1,000
Sobic.003G228400.1	2,483	0,039	1,673	1,000
Sobic.003G228500.1	2,607	0,001	1,278	1,000
Sobic.003G228600.1	2,907	0,016	-1,121	1,000
Sobic.003G228700.1	8,292	0,006	-1,763	0,855
Sobic.003G228900.1	-3,606	0,011	1,860	0,878
Sobic.003G229000.1	2,673	0,053	1,032	1,000
Sobic.003G229100.1	17,423	0,000	-2,185	0,381
Sobic.003G229200.1	-1,396	0,366	1,147	1,000
Sobic.003G235800.1	-4,165	0,323	-10000,000	0,658
Sobic.003G237000.2	-1,116	0,217	-1,019	1,000
Sobic.003G237900.1	6,147	0,000	2,243	0,090
Sobic.003G243400.1	10000,000	0,001	-2,121	0,618
Sobic.003G258800.1	4,997	0,000	-1,000	1,000
Sobic.003G267100.1	-1,172	0,532	1,173	1,000
Sobic.003G267300.1	-1,447	0,000	-1,084	1,000
Sobic.003G267700.1	8,472	0,000	-1,215	0,970
Sobic.003G269300.1	6,071	0,049	-3,493	0,444
Sobic.003G269500.1	17,794	0,000	1,201	1,000
Sobic.003G269600.1	2,268	0,157	1,310	0,365
Sobic.003G271300.1	180,057	0,004	-1,377	1,000
Sobic.003G276700.1	10000,000	0,502	2,834	1,000
Sobic.003G278200.2	10000,000	0,303	1,141	1,000
Sobic.003G278600.1	1,570	0,468	-2,572	0,742
Sobic.003G280400.1	2,150	0,006	1,330	0,368
Sobic.003G281400.1	-1,016	0,634	1,143	1,000
Sobic.003G291800.1	-1,284	0,210	-1,387	0,859
Sobic.003G293300.1	-1,297	0,000	-1,109	0,959
Sobic.003G293600.1	2,117	0,023	-1,164	1,000
Sobic.003G295500.1	10000,000	0,003	-1,228	1,000
Sobic.003G300600.1	-3,840	0,000	1,307	1,000
Sobic.003G304000.1	1,000	1,000	10000,000	0,959
Sobic.003G304100.1	18,841	0,024	-2,640	0,687
Sobic.003G310200.1	-10000,000	0,000	1,000	1,000
Sobic.003G327500.1	3,986	0,000	-1,722	0,869
Sobic.003G343300.1	10000,000	0,421	-10000,000	0,959
Sobic.003G344700.1	-21,218	0,254	2,467	0,919
Sobic.003G348100.1	11,009	0,000	1,387	0,959
Sobic.003G352600.1	1,315	0,141	1,203	0,000
Sobic.003G361300.1	-1,264	0,645	-1,011	1,000
Sobic.003G370200.1	1,579	0,000	-1,050	1,000
Sobic.003G370400.1	7,318	0,001	1,121	1,000
Sobic.003G371000.1	-2,247	0,305	1,926	0,204
Sobic.003G371100.1	-5,131	0,334	1,025	1,000
Sobic.003G371400.1	4,359	0,412	3,499	0,694
Sobic.003G371600.2	-12,926	0,109	2,677	0,386
Sobic.003G372000.1	-1,414	0,000	1,043	1,000
Sobic.003G372500.1	-1,142	0,089	-1,040	1,000
Sobic.003G377700.1	3,384	0,001	1,659	1,000
Sobic.003G378400.2	1,028	1,000	1,044	1,000
Sobic.003G386000.1	-1,604	0,062	-1,003	1,000
Sobic.003G392700.1	-9,880	0,000	1,063	1,000
Sobic.003G392800.1	11,066	0,001	-1,205	1,000
Sobic.003G403000.1	-20,006	0,000	-2,687	0,622
Sobic.003G403300.1	-1,236	0,252	-1,637	0,000
Sobic.003G403900.1	-3,555	0,720	-10000,000	0,959
Sobic.003G413600.1	31,605	0,000	-2,162	0,078
Sobic.003G413700.1	66,121	0,000	-1,078	1,000
Sobic.003G418100.1	1,400	0,000	-1,044	1,000
Sobic.003G421201.1	-1,373	0,000	1,024	1,000
Sobic.003G427000.1	-2,380	0,546	-1,745	1,000
Sobic.003G440900.1	-1,712	0,001	1,351	0,033
Sobic.003G443700.1	10000,000	0,145	1,040	1,000
Sobic.003G445300.2	-2,526	0,804	-1,207	1,000
Sobic.003G445500.1	1,580	0,000	1,133	0,000
Sobic.004G004800.1	-1,670	0,685	1,959	0,959
Sobic.004G011800.1	1,000	1,000	10000,000	0,959
Sobic.004G011900.1	-1,098	0,995	1,269	0,959
Sobic.004G013500.1	-1,683	0,540	1,687	0,959
Sobic.004G019800.2	1,010	1,000	-1,111	1,000
Sobic.004G049300.1	-1,802	0,973	1,532	1,000
Sobic.004G057400.1	1,320	0,000	1,025	1,000
Sobic.004G059800.1	-12,321	0,000	1,923	1,000
Sobic.004G068400.1	1,155	0,375	1,018	1,000
Sobic.004G073500.2	-27,229	0,000	1,051	1,000
Sobic.004G073600.1	1,496	0,186	2,035	0,064
Sobic.004G079900.1	4,381	0,000	1,166	0,959
Sobic.004G086400.1	-2,149	0,195	1,303	1,000
Sobic.004G087700.1	5,594	0,000	-1,052	1,000
Sobic.004G087800.1	7,952	0,000	-1,056	1,000
Sobic.004G099300.2	2,564	0,000	1,125	1,000
Sobic.004G102200.1	-6,755	0,374	-1,359	1,000
Sobic.004G107500.1	1,657	0,796	2,533	0,115
Sobic.004G124500.1	-1,082	1,000	-1,021	1,000
Sobic.004G133500.1	-8,579	0,000	1,093	1,000
Sobic.004G133600.2	10000,000	0,099	-10000,000	0,433
Sobic.004G136600.1	1,577	0,203	-1,176	1,000
Sobic.004G151300.4	-2,104	0,015	-1,466	0,310
Sobic.004G151800.2	1,337	0,013	-1,291	0,290
Sobic.004G157100.1	-2,328	0,590	2,938	0,757
Sobic.004G160000.1	-1,087	1,000	-3,717	0,959
Sobic.004G163800.1	2,140	0,000	-1,242	0,493
Sobic.004G173200.2	-3,155	0,000	1,353	0,831
Sobic.004G176800.1	-1,931	0,000	-1,105	1,000
Sobic.004G188701.1	-47,017	0,000	3,447	0,223
Sobic.004G190500.1	1,016	1,000	1,127	1,000
Sobic.004G193100.1	1,000	1,000	1,000	1,000
Sobic.004G217800.1	-1,051	1,000	1,725	0,083
Sobic.004G221300.1	-1,444	0,020	-1,187	1,000
Sobic.004G222000.1	2,269	0,003	-1,096	1,000
Sobic.004G224000.2	-1,119	0,019	1,004	1,000
Sobic.004G238100.1	2,408	0,009	1,321	0,175
Sobic.004G240900.1	-8,099	0,000	-1,280	0,857
Sobic.004G243200.1	-29,048	0,000	1,386	0,959
Sobic.004G247000.1	-2,183	0,000	1,100	0,895
Sobic.004G250400.2	-10000,000	0,226	1,000	1,000
Sobic.004G250700.1	3,326	0,000	1,108	1,000
Sobic.004G256100.1	2,147	0,425	1,054	1,000
Sobic.004G260500.1	-2,259	0,746	1,291	1,000
Sobic.004G274600.1	1,000	1,000	1,000	1,000
Sobic.004G276200.1	-5,139	0,000	1,083	1,000
Sobic.004G284700.1	-13,543	0,000	-1,067	1,000
Sobic.004G288700.1	-1,162	0,495	-1,086	1,000
Sobic.004G295100.1	1,526	0,409	1,261	0,820
Sobic.004G299500.1	10,080	0,118	3,471	0,068
Sobic.004G299600.2	2,385	0,502	3,440	0,100
Sobic.004G299700.1	1,000	1,000	10000,000	0,959
Sobic.004G301500.2	1,267	1,000	-2,978	1,000
Sobic.004G301600.1	-10000,000	0,286	1,000	1,000
Sobic.004G301650.1	1,000	1,000	1,000	1,000
Sobic.004G309300.1	-6,824	0,545	-2,096	0,057
Sobic.004G332700.1	-36,040	0,297	-10000,000	0,959
Sobic.004G350000.1	-3,412	0,000	1,450	0,445
Sobic.004G351200.2	7,994	0,000	1,107	1,000
Sobic.004G352000.1	-5,262	0,215	-1,153	1,000
Sobic.004G353600.1	-1,262	0,561	-1,120	0,608
Sobic.004G357600.1	1,820	0,502	-2,632	0,715
Sobic.004G358100.2	-18,578	0,150	-4,234	0,430
Sobic.004G358600.1	-3,083	0,000	1,120	0,526
Sobic.005G003200.1	23,452	0,384	-8,126	0,959
Sobic.005G005000.2	13,828	0,036	-2,120	0,927
Sobic.005G005400.1	1,211	0,039	-1,194	0,748
Sobic.005G015450.1	4,321	0,000	1,076	0,959
Sobic.005G015600.1	1,382	1,000	-1,597	1,000
Sobic.005G022100.1	-1,054	0,513	-1,108	0,662
Sobic.005G025900.1	-2,663	0,454	-1,192	1,000
Sobic.005G040700.1	1,000	1,000	1,000	1,000
Sobic.005G046250.1	1,311	0,000	-1,231	0,959
Sobic.005G046600.2	1,000	1,000	1,000	1,000
Sobic.005G052500.1	-1,436	0,805	1,466	1,000
Sobic.005G059300.1	10000,000	0,103	-10000,000	0,444
Sobic.005G073200.1	-1,125	0,981	1,183	0,959
Sobic.005G089600.1	-9,274	0,000	-2,365	0,242
Sobic.005G090600.1	-7,245	0,000	-2,218	0,000
Sobic.005G091600.1	1,259	0,079	-1,067	1,000
Sobic.005G107500.1	-1,204	0,004	1,063	1,000
Sobic.005G110466.1	-1,433	1,000	-4,433	0,657
Sobic.005G123000.1	10000,000	0,085	-2,104	0,959
Sobic.005G123500.1	-3,262	0,011	-1,053	1,000
Sobic.005G124600.1	4,204	0,000	1,546	0,959
Sobic.005G168200.1	13,498	0,000	1,022	1,000
Sobic.005G171200.1	4,197	0,019	1,216	1,000
Sobic.005G191900.1	-1,493	0,126	-1,011	1,000
Sobic.005G195900.1	1,000	1,000	1,000	1,000
Sobic.005G196000.2	-62,462	0,436	2,282	1,000
Sobic.005G196100.1	-20,426	0,020	-1,014	1,000
Sobic.005G196200.2	1,000	1,000	1,000	1,000
Sobic.005G196300.1	-37,465	0,000	-1,324	1,000
Sobic.005G196700.1	-14,163	0,148	-2,550	1,000
Sobic.005G201000.1	-1,192	0,002	1,097	1,000
Sobic.006G003600.1	10000,000	0,502	-10000,000	0,959
Sobic.006G003601.1	10000,000	0,364	1,238	1,000
Sobic.006G014800.1	25,474	0,001	-1,144	1,000
Sobic.006G026800.1	10000,000	0,003	-1,213	1,000
Sobic.006G026900.1	10000,000	0,000	-1,814	0,150
Sobic.006G027300.1	-1,776	0,950	1,557	1,000
Sobic.006G053100.1	2,656	0,000	1,330	0,101
Sobic.006G053200.2	-1,288	0,304	1,087	1,000
Sobic.006G061300.1	3,428	0,000	1,145	0,959
Sobic.006G061700.1	32,913	0,000	-1,442	0,451
Sobic.006G062100.1	1,000	1,000	1,000	1,000
Sobic.006G074100.1	-10000,000	0,359	1,000	1,000
Sobic.006G075200.1	-1,846	0,926	-3,925	0,828
Sobic.006G075300.1	1,000	1,000	10000,000	0,959
Sobic.006G075350.1	10000,000	0,017	-10000,000	0,111
Sobic.006G089300.1	-10,075	0,075	1,402	1,000
Sobic.006G093400.1	-8,236	0,184	1,376	1,000
Sobic.006G101900.1	-5,430	0,000	2,424	0,019
Sobic.006G102300.1	2,006	0,697	1,314	1,000
Sobic.006G102400.1	-8,324	0,000	-1,434	1,000
Sobic.006G102500.1	-4,684	0,000	1,048	1,000
Sobic.006G102800.1	-2,375	0,015	1,135	1,000
Sobic.006G105600.1	-5,255	0,033	1,804	0,416
Sobic.006G123100.1	5,800	0,004	1,100	1,000
Sobic.006G130400.1	33,111	0,000	1,253	0,076
Sobic.006G130501.1	1,661	0,041	1,497	0,871
Sobic.006G130700.1	-10000,000	0,502	1,000	1,000
Sobic.006G130800.1	1,417	0,000	1,062	1,000
Sobic.006G143100.1	-5,803	0,000	1,702	0,001
Sobic.006G143900.1	2,259	0,558	1,688	0,863
Sobic.006G149400.1	-1,029	1,000	-1,080	1,000
Sobic.006G150100.1	1,681	0,196	1,555	1,000
Sobic.006G152500.1	3,938	0,000	1,678	0,727
Sobic.006G152600.1	1,332	0,465	1,886	0,000
Sobic.006G152700.2	-1,276	1,000	-1,300	1,000
Sobic.006G152801.1	-1,026	1,000	1,248	1,000
Sobic.006G152900.1	10000,000	0,001	1,453	1,000
Sobic.006G155200.1	-3,229	0,745	-10000,000	0,959
Sobic.006G155300.1	1,000	1,000	1,000	1,000
Sobic.006G156600.2	2,372	0,000	1,108	0,959
Sobic.006G161800.1	-963,376	0,000	1,512	1,000
Sobic.006G164200.1	2,239	0,000	1,182	0,749
Sobic.006G164300.1	1,354	0,544	-1,016	1,000
Sobic.006G165400.1	1,185	0,477	1,315	1,000
Sobic.006G170300.1	1,733	0,380	-1,100	1,000
Sobic.006G170500.2	-10000,000	0,502	1,000	1,000
Sobic.006G170600.1	-2,580	0,201	1,674	0,927
Sobic.006G173700.1	2,641	0,000	-1,080	1,000
Sobic.006G173800.1	-3,051	0,000	-1,262	0,457
Sobic.006G176700.1	-1,080	1,000	1,116	1,000
Sobic.006G181900.1	-12,451	0,148	2,134	1,000
Sobic.006G201100.1	-4,909	0,000	1,361	0,794
Sobic.006G201200.1	14,023	0,039	-7,701	0,299
Sobic.006G208100.2	10000,000	0,083	2,426	1,000
Sobic.006G210700.1	2,163	0,000	-1,021	1,000
Sobic.006G212200.1	-2,142	0,279	4,706	0,959
Sobic.006G213500.1	-1,077	1,000	-2,071	0,354
Sobic.006G220800.1	-2,830	0,000	1,028	1,000
Sobic.006G221000.1	2,606	0,044	-1,308	1,000
Sobic.006G232300.1	2,315	0,691	-1,359	1,000
Sobic.006G236700.1	-1,499	0,373	1,206	1,000
Sobic.006G244000.1	-2,996	0,071	2,958	0,842
Sobic.006G249400.1	1,654	0,433	-2,123	0,009
Sobic.006G251200.1	-1,728	0,502	-1,169	1,000
Sobic.006G255600.1	-6,729	0,003	1,274	0,959
Sobic.006G259300.1	10000,000	0,006	-1,187	1,000
Sobic.006G264700.1	1,000	1,000	10000,000	0,959
Sobic.006G268800.1	-13,133	0,205	-1,808	0,831
Sobic.006G276300.1	-10000,000	0,145	1,000	1,000
Sobic.006G279700.1	-5,869	0,000	1,199	0,547
Sobic.006G280500.1	-1,898	0,197	1,201	0,446
Sobic.006G281700.2	-2,147	0,000	-1,169	0,510
Sobic.007G004300.1	-1,236	0,794	-1,054	1,000
Sobic.007G014700.1	-1,070	0,692	-1,040	0,256
Sobic.007G020600.1	-1,571	0,000	-1,245	0,710
Sobic.007G030700.1	1,592	0,003	1,004	1,000
Sobic.007G038500.1	2,532	0,754	-1,183	1,000
Sobic.007G039500.1	10000,000	0,005	1,222	1,000
Sobic.007G039600.1	10000,000	0,090	-1,281	1,000
Sobic.007G042600.1	166,312	0,000	-2,921	0,106
Sobic.007G042700.1	10000,000	0,005	-1,047	1,000
Sobic.007G044300.1	-5,246	0,000	1,224	0,050
Sobic.007G061800.1	1,000	1,000	1,000	1,000
Sobic.007G064500.1	10000,000	0,015	-1,562	1,000
Sobic.007G064900.1	3,392	0,491	-1,765	1,000
Sobic.007G065500.1	3,150	0,000	-1,170	0,000
Sobic.007G068200.1	4,141	0,000	-1,399	0,558
Sobic.007G075100.2	1,038	1,000	-1,486	0,016
Sobic.007G075700.1	-5,965	0,000	-1,021	1,000
Sobic.007G081300.1	-4,955	0,019	1,044	1,000
Sobic.007G095100.1	10000,000	0,001	-1,193	1,000
Sobic.007G123500.3	4,311	0,000	1,012	1,000
Sobic.007G124600.1	1,000	1,000	1,000	1,000
Sobic.007G130500.1	2,001	0,000	1,042	1,000
Sobic.007G133600.1	-4,059	0,050	-1,190	1,000
Sobic.007G153001.1	-1,236	1,000	-9,360	0,540
Sobic.007G160900.1	1,000	1,000	1,000	1,000
Sobic.007G163800.1	-4,228	0,000	1,362	0,023
Sobic.007G164400.2	4,190	0,092	-8,610	0,193
Sobic.007G191200.1	10000,000	0,002	1,033	1,000
Sobic.007G202700.1	-1,838	0,566	-1,418	1,000
Sobic.007G209900.1	1,000	1,000	1,000	1,000
Sobic.007G213200.1	-1,970	0,931	1,555	1,000
Sobic.007G214500.1	18,839	0,000	1,171	1,000
Sobic.007G225000.2	2,245	0,000	1,123	0,864
Sobic.008G006100.1	-2,431	0,008	-1,268	1,000
Sobic.008G014700.1	-7,876	0,257	1,759	0,997
Sobic.008G018200.1	1,874	0,061	-1,051	1,000
Sobic.008G081200.1	2,728	0,283	1,584	0,927
Sobic.008G081400.1	-4,172	0,319	1,621	0,812
Sobic.008G081500.1	-29,272	0,525	9,044	0,295
Sobic.008G094000.1	-3,403	0,000	-1,391	1,000
Sobic.008G094300.1	1,000	1,000	1,000	1,000
Sobic.008G094400.1	-4,362	0,000	1,046	1,000
Sobic.008G111100.1	-1,842	0,521	1,481	1,000
Sobic.008G111300.2	-41,407	0,359	-1,511	1,000

Name	Impact of mycorrhization				Impact of phosphate			
	in Low Phosphate (Myc+ LP VS. Myc- LP)		in High Phosphate (Myc+ HP VS. Myc- HP)		in non mycorhized plants (Myc- HP VS. Myc- LP)		in mycorrhized plants (Myc+ HP VS. Myc+ LP)	
	Baggerley Fold Change	Baggerley FDR	Baggerley Fold Change	Baggerley FDR	Baggerley Fold Change	Baggerley FDR	Baggerley Fold Change	Baggerley FDR
Potri.001G013400.1	1,000	1,000	1,000	1,000	1,000	1,000	1,000	1,000
Potri.001G027100.1	1,252	0,755	2,024	0,001	-1,201	1,000	1,347	0,391
Potri.001G045200.1	2,417	0,000	-1,183	0,931	1,293	0,922	-2,211	0,000
Potri.001G046800.1	-1,216	0,866	1,306	0,669	-1,050	1,000	1,512	0,512
Potri.001G060900.1	3,893	0,489	-20,170	0,632	18,658	0,854	-4,208	0,517
Potri.001G068600.1	-2,082	0,584	2,034	0,392	1,246	1,000	5,277	0,018
Potri.001G069600.1	-4,005	0,000	1,165	1,000	-1,571	0,892	2,970	0,000
Potri.001G085900.1	6,046	0,201	22,967	0,000	-2,250	0,554	1,689	0,492
Potri.001G088700.1	2,899	0,000	6,009	0,281	-1,087	1,000	1,906	0,524
Potri.001G097300.1	1,000	1,000	1,000	1,000	1,000	1,000	1,000	1,000
Potri.001G110400.1	-10000,000	0,111	4,824	0,657	-2,641	0,864	10000,000	0,351
Potri.001G123700.1	-4,749	0,000	-1,150	1,000	-1,850	0,717	2,232	0,008
Potri.001G123800.1	1,245	0,507	3,039	0,000	-1,679	0,002	1,453	0,193
Potri.001G124800.1	1,000	1,000	1,000	1,000	1,000	1,000	1,000	1,000
Potri.001G130800.1	-1,072	0,885	3,007	0,000	-1,244	0,942	2,591	0,000
Potri.001G144300.1	-6,542	0,000	-1,007	1,000	-1,161	1,000	5,594	0,038
Potri.001G145200.1	-2,132	0,019	2,010	0,322	-2,312	0,417	1,854	0,022
Potri.001G157000.1	-37,036	0,000	-5,931	0,004	1,270	0,895	7,929	0,000
Potri.001G168800.1	5,773	0,040	4,623	0,000	-1,152	1,000	-1,438	0,597
Potri.001G179400.1	-5,401	0,002	3,045	0,000	-3,815	0,056	4,311	0,000
Potri.001G185300.1	-1,989	0,022	1,040	1,000	-1,221	0,929	1,694	0,049
Potri.001G185700.1	-4,248	0,002	-2,655	0,344	-1,019	1,000	1,571	0,059
Potri.001G186700.1	-3,405	0,000	-1,560	0,771	-1,313	0,942	1,663	0,383
Potri.001G205500.1	-1,316	0,003	1,303	0,527	1,059	1,000	1,816	0,004
Potri.001G217200.1	-10000,000	0,143	-1,936	1,000	-1,232	1,000	10000,000	0,295
Potri.001G217300.1	34,290	0,005	10000,000	0,318	-10000,000	0,563	-2,944	0,170
Potri.001G217400.1	181,839	0,000	10000,000	0,161	-10000,000	0,779	-3,582	0,000
Potri.001G235300.1	-12,099	0,000	-1,106	1,000	-1,429	0,413	7,655	0,000
Potri.001G244100.1	1,111	0,551	1,178	0,741	-1,130	1,000	-1,066	0,803
Potri.001G246000.1	-10000,000	0,489	-10000,000	0,657	-6,349	0,925	1,000	1,000
Potri.001G248600.1	10000,000	0,134	72,004	0,225	10000,000	0,322	3,877	0,329
Potri.001G248900.1	1,000	1,000	1,000	1,000	1,000	1,000	1,000	1,000
Potri.001G253800.1	2,506	0,180	1,664	0,443	1,628	0,902	1,081	1,000
Potri.001G266900.1	1,800	0,269	3,080	0,000	-1,045	1,000	1,637	0,120
Potri.001G272900.1	3,075	0,009	4,125	0,000	-1,170	1,000	1,146	0,880
Potri.001G305400.1	1,871	0,390	12,196	0,000	-1,271	1,000	5,129	0,000
Potri.001G317600.1	1,216	0,867	7,344	0,000	-2,951	0,231	2,047	0,078
Potri.001G318500.1	-10000,000	0,088	-1,219	1,000	-2,240	0,922	10000,000	0,524
Potri.001G326000.1	137,166	0,075	55,289	0,205	2,546	0,947	1,026	1,000
Potri.001G344300.1	1,000	1,000	1,000	1,000	1,000	1,000	1,000	1,000
Potri.001G351200.1	-8,982	0,000	-4,484	0,000	-1,003	1,000	1,997	0,002
Potri.001G355500.1	5,354	0,274	4,835	0,016	-1,016	1,000	-1,125	1,000
Potri.001G383000.1	1,681	0,001	1,063	1,000	1,197	1,000	-1,322	0,148
Potri.001G455000.1	-5,308	0,000	1,303	0,822	-1,537	0,582	4,499	0,004
Potri.002G005500.1	3,546	0,000	3,405	0,000	-1,571	0,131	-1,636	0,000
Potri.002G006200.1	-2,072	0,000	-1,074	0,959	-1,261	0,430	1,531	0,000
Potri.002G029200.1	-3,279	0,215	-5,810	0,000	1,634	0,746	-1,084	1,000
Potri.002G038900.1	-2,729	0,025	-3,716	0,000	2,438	0,000	1,790	0,523
Potri.002G046500.1	2,001	0,139	13,450	0,000	-2,572	0,779	2,613	0,000
Potri.002G049500.1	-14,342	0,000	-28,122	0,092	1,307	1,000	-1,500	0,504
Potri.002G054300.1	1,000	1,000	1,000	1,000	1,000	1,000	1,000	1,000
Potri.002G055100.1	-2,838	0,000	1,589	0,534	-1,762	0,446	2,559	0,001
Potri.002G069600.1	92,600	0,011	230,047	0,047	-2,646	0,655	-1,065	1,000
Potri.002G072600.1	8,997	0,036	25,677	0,394	-3,899	0,732	-1,366	0,910
Potri.002G072700.1	-10000,000	0,463	10000,000	0,006	-10000,000	0,854	10000,000	0,002
Potri.002G072800.1	1,000	1,000	-10000,000	0,657	10000,000	0,854	1,000	1,000
Potri.002G086100.1	1,863	0,389	7,817	0,000	-1,285	1,000	3,264	0,002
Potri.002G092400.1	-17,301	0,000	-2,724	0,317	1,195	1,000	7,591	0,008
Potri.002G092500.1	-7,453	0,000	-3,585	0,334	1,762	0,854	3,664	0,000
Potri.002G095900.1	-2,364	0,000	1,382	0,585	-1,340	0,854	2,439	0,000
Potri.002G096000.1	-1,772	0,385	2,586	0,130	-2,043	0,622	2,244	0,160
Potri.002G097000.1	132,413	0,004	536,324	0,203	-2,479	0,966	1,634	0,714
Potri.002G106900.1	-2,079	0,000	1,098	1,000	-1,255	0,886	1,819	0,117
Potri.002G118900.1	-2,734	0,000	-2,964	0,701	1,993	0,978	1,839	0,000
Potri.002G129500.1	2,779	0,222	12,135	0,000	-3,618	0,318	1,207	0,862
Potri.002G134500.1	-2,192	0,053	1,411	0,443	-1,769	0,498	1,748	0,082
Potri.002G173600.1	-10000,000	0,141	1,000	1,000	-10000,000	0,494	1,000	1,000
Potri.002G174600.1	11,012	0,020	19,265	0,000	-1,145	1,000	1,528	0,488
Potri.002G202300.1	-2,708	0,000	-1,442	0,000	-1,062	0,007	1,769	0,000
Potri.002G212900.1	-2,512	0,000	-1,389	0,434	1,005	1,000	1,819	0,000
Potri.002G217700.1	-10000,000	0,121	-10000,000	0,657	-1,796	1,000	1,000	1,000
Potri.002G221300.1	-2,617	0,000	1,132	1,000	-1,118	1,000	2,649	0,000
Potri.002G225500.1	-3,421	0,129	-7,103	0,657	2,686	0,970	1,294	0,634
Potri.002G227500.1	1,656	0,000	1,589	0,069	-1,433	0,337	-1,494	0,002
Potri.002G237500.1	-1,796	0,023	1,391	0,440	1,140	0,872	2,848	0,004
Potri.002G245600.1	-5,073	0,000	-1,397	0,767	-1,250	0,958	2,905	0,001
Potri.002G249100.1	-3,345	0,000	-1,022	1,000	-1,236	0,783	2,649	0,049
Potri.002G255000.1	1,193	0,956	1,873	0,381	-1,259	1,000	1,248	0,848
Potri.002G255100.1	-10000,000	0,089	-1,219	1,000	-3,152	0,854	10000,000	0,524
Potri.002G258700.1	1,913	0,369	9,510	0,000	-2,154	0,679	2,307	0,004
Potri.002G258900.1	54,628	0,152	10000,000	0,288	-10000,000	0,445	1,587	0,836
Potri.002G259900.1	-3,331	0,000	-1,287	0,673	-1,150	0,978	2,252	0,000
Potri.002G263800.1	-2,411	0,000	1,515	0,132	-1,231	0,866	2,966	0,000
Potri.003G000800.1	-18,582	0,000	-19,279	0,263	1,871	0,898	1,804	0,269
Potri.003G008800.1	-1,170	0,528	1,240	0,358	-1,152	0,931	1,258	0,151
Potri.003G018800.1	-1,633	0,264	2,006	0,108	-2,143	0,419	1,529	0,199
Potri.003G023900.1	-2,489	0,000	1,091	0,970	-1,105	1,000	2,456	0,000
Potri.003G050900.1	-2,874	0,216	-1,757	0,847	-1,812	0,854	-1,108	1,000
Potri.003G056200.1	-3,101	0,000	1,116	0,978	-1,789	0,007	1,934	0,002
Potri.003G065400.1	2,531	0,019	2,478	0,000	-1,134	1,000	-1,158	0,758
Potri.003G077800.1	-6,556	0,000	-10,937	0,020	1,551	0,854	-1,075	1,000
Potri.003G080500.1	87,267	0,108	108,632	0,349	1,131	1,000	1,408	0,952
Potri.003G088800.1	-13,811	0,000	-4,019	0,608	1,021	1,000	3,510	0,092
Potri.003G103100.1	1,217	0,703	4,544	0,000	-2,654	0,062	1,407	0,263
Potri.003G108500.1	-10000,000	0,489	-4,623	0,807	6,993	0,918	10000,000	0,524
Potri.003G109700.1	-1,728	0,000	-1,152	0,514	1,257	0,354	1,885	0,000
Potri.003G109800.1	-1,227	1,000	-1,431	0,964	1,144	1,000	-1,020	1,000
Potri.003G111500.1	-20,727	0,000	-6,458	0,033	1,336	0,958	4,287	0,062
Potri.003G112600.1	-3,466	0,000	-1,746	0,757	1,073	1,000	2,131	0,337
Potri.003G121300.1	1,000	1,000	1,000	1,000	1,000	1,000	1,000	1,000
Potri.003G126300.1	10000,000	0,489	10000,000	0,657	1,000	1,000	-2,637	0,820
Potri.003G128600.1	-1,596	0,002	1,044	0,590	-1,129	0,854	1,476	0,000
Potri.003G133900.1	-2,329	0,000	1,217	0,447	-1,094	1,000	2,590	0,000
Potri.003G134100.1	6,888	0,002	5,942	0,080	-1,849	0,914	-2,143	0,149
Potri.003G138300.1	-2,522	0,000	1,086	0,868	-1,078	1,000	2,542	0,000
Potri.003G142000.1	-1,338	0,519	2,506	0,468	-1,799	0,570	1,864	0,510
Potri.003G143100.1	1,000	1,000	-10000,000	0,657	10000,000	0,854	1,000	1,000
Potri.003G145000.1	1,236	0,821	3,253	0,038	-1,440	1,000	1,827	0,123
Potri.003G148200.1	-1,300	0,042	1,480	0,569	-1,404	0,854	1,370	0,089
Potri.003G166800.1	1,000	1,000	1,000	1,000	1,000	1,000	1,000	1,000
Potri.003G180900.1	-1,610	0,606	1,984	0,608	1,415	1,000	4,520	0,113
Potri.003G197400.1	-10000,000	0,002	-7,567	0,001	2,102	0,354	10000,000	0,118
Potri.003G212900.1	1,000	1,000	10000,000	0,657	1,000	1,000	10000,000	0,524
Potri.004G033600.1	-3,397	0,000	-1,022	1,000	-1,143	0,848	2,907	0,000
Potri.004G039100.1	2,459	0,281	1,740	0,011	-1,105	1,000	-1,562	0,613
Potri.004G063000.1	-4,390	0,000	1,556	0,290	-1,525	0,588	4,479	0,000
Potri.004G064100.1	3893,363	0,028	1786,547	0,054	1,844	1,000	-1,182	1,000
Potri.004G068500.1	-2,054	0,001	1,900	0,059	1,041	1,000	4,064	0,000
Potri.004G081300.1	-6,110	0,000	1,026	1,000	-1,495	0,965	4,192	0,000
Potri.004G083300.1	1,706	0,000	3,206	0,073	1,371	1,000	2,576	0,007
Potri.004G089800.1	-1,223	0,865	3,009	0,004	-1,737	0,599	2,117	0,087
Potri.004G093400.1	-2,324	0,422	1,377	0,826	-1,220	1,000	2,622	0,363
Potri.004G094700.1	-2,374	0,362	4,110	0,372	-1,941	0,421	5,028	0,249
Potri.004G094800.1	-10000,000	0,027	-1,005	1,000	-1,030	1,000	10000,000	0,033
Potri.004G094900.1	-10000,000	0,000	-11,000	0,435	-1,023	1,000	10000,000	0,006
Potri.004G097800.1	-1,818	0,000	-1,597	0,000	1,205	0,227	1,371	0,000
Potri.004G100400.1	-1,164	0,613	2,918	0,173	1,321	1,000	4,486	0,018
Potri.004G101900.1	1,000	1,000	1,000	1,000	1,000	1,000	1,000	1,000
Potri.004G132200.1	-3,490	0,079	-2,728	0,324	-1,014	1,000	1,262	1,000
Potri.004G152300.1	-1,087	0,790	-2,379	0,000	1,430	0,016	-1,531	0,000
Potri.004G167000.1	-11,264	0,048	-1,344	1,000	-1,158	1,000	7,240	0,005
Potri.004G167500.1	-2,613	0,000	1,797	0,043	-1,165	1,000	4,029	0,000
Potri.004G176300.1	-2,590	0,000	1,351	0,544	-1,337	0,854	2,618	0,000
Potri.004G183600.1	1,300	0,011	1,071	0,940	1,174	0,854	-1,034	0,887
Potri.004G186500.1	-1,316	0,025	1,272	0,747	1,098	1,000	1,838	0,000
Potri.004G207100.1	-1,440	0,001	1,403	0,197	-1,147	0,865	1,761	0,001
Potri.004G216500.1	-10000,000	0,061	1,548	1,000	-5,779	0,560	10000,000	0,359
Potri.004G229000.1	-1,469	0,400	3,476	0,032	-2,494	0,097	2,047	0,149
Potri.004G233500.1	-3,650	0,000	-5,549	0,209	1,661	0,872	1,093	1,000
Potri.005G001400.1	9219,190	0,072	1379,751	0,401	5,950	0,929	-1,123	1,000
Potri.005G010800.1	-1,418	0,003	1,292	0,178	-1,257	0,512	1,457	0,001
Potri.005G023900.1	-10000,000	0,250	1,184	1,000	-1,058	1,000	10000,000	0,224
Potri.005G034500.1	-1,063	0,894	1,797	0,000	-1,041	0,854	1,834	0,000
Potri.005G035200.1	-1,130	0,489	1,346	0,153	1,087	1,000	1,653	0,000
Potri.005G037000.1	-1,675	0,001	1,095	1,000	1,088	1,000	1,996	0,000
Potri.005G039900.1	-2,456	0,006	1,551	0,809	-1,953	0,854	1,952	0,004
Potri.005G040000.1	-1,429	0,026	1,095	1,000	1,245	0,877	1,950	0,089
Potri.005G079000.1	-1,796	0,429	9,833	0,005	-6,983	0,001	2,529	0,186
Potri.005G090700.1	-10000,000	0,111	-2,550	0,495	2,552	0,768	10000,000	0,151
Potri.005G095100.1	-18,708	0,000	4,870	0,091	-2,983	0,345	30,536	0,004
Potri.005G095900.1	33,416	0,001	51,271	0,415	1,188	1,000	1,823	0,746
Potri.005G098900.1	-8,929	0,000	-2,105	0,123	-1,218	1,000	3,483	0,010
Potri.005G102800.1	-4,985	0,002	-1,371	0,839	-1,881	0,584	1,933	0,281
Potri.005G106000.1	4,544	0,645	10,038	0,236	4,187	0,854	9,249	0,155
Potri.005G109200.1	54,237	0,465	4,969	0,327	-1,332	1,000	-14,542	0,524
Potri.005G109300.1	10000,000	0,489	1,000	1,000	1,000	1,000	-10000,000	0,524
Potri.005G167300.1	-57,756	0,015	-18,480	0,000	1,164	1,000	3,637	0,087
Potri.005G169300.1	1,000	1,000	1,000	1,000	1,000	1,000	1,000	1,000
Potri.005G175300.1	1,314	0,795	5,895	0,000	-2,329	0,301	1,926	0,074
Potri.005G175500.1	-10000,000	0,489	10000,000	0,657	-10000,000	0,854	10000,000	0,524
Potri.005G175700.1	1,000	1,000	1,000	1,000	1,000	1,000	1,000	1,000
Potri.005G187300.1	-1,169	0,928	2,133	0,016	-1,393	0,905	1,789	0,078
Potri.005G208500.1	-4,796	0,000	1,619	0,801	-1,318	1,000	5,894	0,001
Potri.005G213500.1	-154,130	0,053	-33,659	0,093	1,041	1,000	4,768	0,008
Potri.005G216400.1	4,623	0,036	17,000	0,000	-2,424	0,649	1,517	0,263
Potri.005G223500.1	1,470	0,173	4,288	0,000	-2,812	0,000	1,038	1,000
Potri.005G223600.1	1,234	0,414	3,723	0,000	-5,404	0,000	-1,791	0,025
Potri.005G223700.1	1,000	1,000	10000,000	0,657	1,000	1,000	10000,000	0,524
Potri.005G233500.1	148,508	0,015	40,992	0,092	1,752	0,854	-2,068	0,397
[illegible]	[illegible]	[illegible]	[illegible]	[illegible]	[illegible]	[illegible]	[illegible]	[illegible]
