# Supplementary material for: Imbalanced Regulation of Fungal Nutrient Transports According to Phosphate Availability in a Symbiocosm Formed by Poplar, Sorghum, and Rhizophagus irregularis
Source: Front Plant Sci. 2019 Dec 12;10:1617. doi: 10.3389/fpls.2019.01617 (PMC6920215; doi:10.3389/fpls.2019.01617)
Supplement: Table S5 — Name of fungal genes regulated by P treatment and during the interaction with P. trichocarpa and S. bicolor. [file Table_5.pdf]

Fungal expressed genes up-regulated by P treatment					Fungal expressed genes down-regulated by P treatment					Fungal expressed genes up regulated during interaction with sorghum			Fungal expressed genes up regulated during interaction with poplar		
Effect of HP regardless of the fungal compartment (ERM or IRM) (0/1)	Effect of HP *in planta* (IRM) (2/52)	Effect of HP specifically expressed in sorghum (13/246)	Effect of HP specifically expressed in poplar (237/3110)	Effect of HP specifically expressed in ERM (3/73)	Repressed by HP regardless of the fungal compartment (ERM or IRM) (0/0)	Repressed by HP *in planta* (0/4)	Repressed by HP specifically in sorghum (0/26)	Repressed by HP specifically in poplar (6/264)	Repressed by HP specifically in ERM (7/209)	Effect of sorghum regardless of [Phosphate] (144/1443)	Effect of sorghum in LP (207/3190)	Effect of sorghum in HP (30/600)	Effect of poplar regardless of [Phosphate] (3/389)	Effect of poplar in LP (14/484)	Effect of poplar in HP (1/353)
	Ves18	Transp189	Ves94	MD-2_18				Transp222	SugTrans19	Transp118	Transp2	Transp144	Transp209	Transp222	Ves25
	Ves92	Ves84	Transp280	MD-2_9				Ves73	Transp255	Transp195	GS5	Lip17	Transp157	Transp193	
		Transp224	Lip25	Transp173				Transp248	Transp115	Transp196	Transp280	Lip8	Ves20	Transp223	
		Ves103	Transp26					Transp288	Desat3	Desat6	Transp66	Ves84		Ves16	
		Transp277	Transp93					Ves20	GintAMT2	Transp135	Lip14	MD-2_12		Ves73	
		Ves82	Lip14					Transp157	MD-2_13	Ves21	Lip15	Transp17		Ves23	
		NAR3	Transp149						Transp242	SugTrans47	Lip25	Transp244		Transp258	
		MD-2_25	Transp135							Transp261	Transp112	SugTrans21		Transp115	
		Transp251	TransVes9							Ves116	Lip5	Lip63		SugTrans11	
		Ves7	Transp196							Ves106	Transp39	Transp159		Transp277	
		Transp74	RiPT11							NAR5	Transp26	Transp268		Transp82	
		Ves60	Lip12							TransVes8	Lip53	Transp224		Transp264	
		Transp219	Transp80							Transp167	Transp29	Transp297		Ves61	
			Lip24							Ves24	Desat8	TransVes17		Transp248	
			Ves21							SugTrans6	TransVes9	TransVes21			
			Ves28							Transp273	Ves28	Transp153			
			Ves116							Transp151	Transp110	SugTrans52			
			Desat8							GS3	Transp149	Transp178			
			Ves106							AOAT	Transp80	Lip31			
			Transp296							RiHAK	Lip12	Transp44			
			Transp20							URE	Lip19	Ves72			
			RiPT7							SugTrans1	Transp176	Lip66			
			Transp195							Transp20	Transp182	Transp93			
			AOAT							RiPT6	Desat3	Ves103			
			SugTrans46							Transp98	Transp107	SugTrans25			
			TransVes8							Transp174	NAR4	Transp143			
			Transp207							Transp270	RiPT7	Desat4			
			Transp151							Transp179	RiPT11	Transp251			
			SugTrans6							Transp232	Transp253	Ves100			
			Transp282							RiPT3	Transp111	SugTrans51			
			GS3							Transp259	Lip24				
			NAR1							NAR3	Ves113				
			SugTrans33							SugTrans30	Lip28				
			Ves111							Transp299	Transp282				
			Transp75							Transp218	Ves111				
			Lip31							Transp192	Transp95				
			Transp164							Ves53	Transp281				
			Transp98							Ves35	Transp208				
			SugTrans24							SugTrans33	NIR3				
			Transp259							Ves5	SugTrans46				
			Transp158							Transp75	SugTrans31				
			Ves113							Ves52	GS2				
			Lip28							Transp201	GS1				
			Lip21							Transp3	Transp207				
			Desat3							Transp154	Lip62				
			Transp243							Lip21	Transp124				
			NAR4							Transp128	NAR1				
			Transp261							Transp181	Desat7				
			Transp281							Ves31	Desat2				
			Ves46							SugTrans5	Transp237				
			Transp121							Lip60	Transp202				
			Transp131							Elo1	Desat5				
			SugTrans22							Transp172	SugTrans22				
			SugTrans2							Transp101	Ves77				
			Transp292							Ves99	Ves50				
			Ves52							Transp99	Transp131				
			Transp3							Transp163	Lip41				
			Transp208							RiPT1	Lip56				
			Transp172							Transp90	Lip47				
			Transp210							VBA2	RTC1				
			Transp88							Ves10	GintAMT2				
			Ves50							Lip64	OTA				
			URE							SugTrans34	Transp155				
			Transp79							Ves98	SugTrans2				
			Ves55							Lip69	Transp185				
			Transp22							Ves105	CPS2				
			GintAMT3							Ves96	Lip49				
			Ves53							Transp104	Ves85				
			SugTrans30							Ves11	SugTrans12				
			Transp95							SugTrans57	AGK/AGPR				
			SugTrans1							Transp283	Transp260				
			SugTrans41							SugTrans39	Transp243				
			Ves51							Transp306	Ves51				
			Transp232							Ves56	Transp162				
			Transp237							Ves46	Transp206				
			RTC1							Transp108	Lip4				
			Lip44							Ves119	Transp1				
			Transp273							TransVes3	Lip55				
			SugTrans4							Lip48	SugTrans24				
			Transp123							SugTrans54	Transp24				
			Transp197							Transp19	Transp52				
			Lip47							Ves65	Lip40				
			Transp218							Transp5	Ves91				
			GintAMT1							Transp173	GintAMT1				
			Ves78							Transp238	Transp184				
			Transp122							MST5	Elo2				
			SugTrans54							TransVes12	Ves34				
			NRT1							RTC2	Ves101				
			Ves76							Phosphatase1	Transp83				
			SugTrans5							Lip65	Transp269				
			Ves97							SugTrans14	Transp88				
			Lip60							Transp279	Transp158				
			Transp24							Transp285	Ves115				
			TransVes3							Transp177	Transp171				
			Transp265							SugTrans29	Transp86				
			Ves80							Transp38	Lip52				
			Transp19							GS4	RiPT2				
			Ves11							Transp106	GintAMT3				
			Transp154							Transp290	Transp33				
			Transp92							SugTrans27	MD-2_1				
			Transp171							Transp289	Ves97				
			Transp167							Ves70	Transp215				
			OTA							Transp156	Transp22				
			Lip62							SugTrans50	Transp301				
			SugTrans12							Transp137	Ves87				
			Transp99							Elo4	Ves55				
			Lip56							Ves2	SugTrans4				
			NIR2							Ves36	Transp164				
			Ves115							TransVes10	Polyphosphatase2				
			Transp107							Transp12	NRT1				
			Transp245							Transp231	Ves80				
			Transp179							MST4	Transp226				
			Transp162							SugTrans55	Transp79				
			Transp206							Transp166	Transp197				
			Transp187							Bind1	MD-2_13				
			GS1							SugTrans38	Transp123				
			OCT							Transp276	Transp216				
			Transp111							Transp41	ASL				
			Desat5							SugTrans58	Ves68				
			Ves87							TransVes4	Transp105				
			SugTrans14							Transp130	Transp109				
			Ves101							Transp10	Lip35				
			Lip52							RiPT9	OCT				
			AGK/AGPR							Lip16	Ves76				
			Lip57							Transp142	Ves30				
			Transp247							Transp21	Ves4				
			Transp240							Transp165	Ves110				
			Ves66							SugTrans16	Transp40				
			Ves85							Transp168	Ves93				
			Transp109							Transp140	Transp121				
			Elo2							SugTrans49	Transp298				
			Desat7							Ves107	SugTrans41				
			Ves14							SugTrans40	Transp203				
			SugTrans39							SugTrans32	Ves78				
			Transp101							Transp252	Transp187				
			Ves56							Transp23	Transp296				
			Lip4							Transp234	Ves88				
			Desat2							Transp180	Transp249				
			Transp205							RiPT10	Transp205				
			Transp84							Ves86	Transp242				
			Transp40							Ves38	Ves66				
			SugTrans34							Ves15	Transp78				
			Ves110							Ves90	Ves120				
			Bind1							Ves82	Ves29				
			Lip49								Transp271				
			Polyphosphatase2								SUC1				
			TransVes12								Transp210				
			Ves91								SugTrans15				
			Transp298								Lip36				
			Transp270								RiPT5				
			Transp125								Transp74				
			Ves65								Transp94				
			Ves105								Transp127				
			NIR3								GOGAT				
			Transp181								Transp211				
			Transp174								Transp292				
			Ves68								Transp81				
			Transp231								SugTrans20				
			Transp52								Transp92				
			Ves2								NIR2				
			Ves93								Ves57				
			Transp97								Phosphatase2				
			Transp276								ODC				
			Transp199								Transp186				
			Transp128								Lip57				
			Ves120								Transp245				
			Transp104								SugTrans45				
			Transp283								Transp305				
			Ves112								Ves104				
			MD-2_1								Transp125				
			SugTrans3								Transp200				
			Ves34								Lip38				
			CPS2								TransVes13				
			NAR5								Ves117				
			Lip59								Lip18				
			SugTrans16								Transp146				
			Transp203								Transp254				
			Transp305								Transp122				
			Lip64								Ves54				
			ODC								VTC2-3				
			Transp43								Transp265				
			Transp156								TransVes5				
			Lip36								Lip54				
			Ves4								Transp150				
			Ves10								Transp85				
			Transp192								NRT3				
			GS4								Desat9				
			Transp285								SugTrans3				
			Transp211								TransVes6				
			Transp216								Ves39				
			Lip48								Ves112				
			Transp254								Ves1				
			Lip18								Ves75				
			Ves27								SugTrans7				
			ASL								Transp256				
			Ves70								TransVes11				
			SugTrans20								Lip3				
			AQPF2								Ves27				
			Ves30								VTC4				
			Transp279								Bind4				
			Transp150								Ves95				
			Ves54								Transp97				
			Transp21								MST6				
			Transp127								Transp235				
			Transp177								RiPT8				
			Ves96								Ves45				
			SUC1								Lip68				
			Ves40												
			Transp137												
			Transp41												
			Elo1												
			Lip65												
			SugTrans57												
			Ves1												
			Transp306												
			Desat9												
			TransVes4												
			SugTrans55												
			Lip3												
			SugTrans38												
			SugTrans27												
			MD-2_13												
			Transp256												
			VBA1												
			Transp77												
			OAT												
			GOGAT												
			Ves88												
			Transp103												
			Transp201												
			Bind4												
			Ves58												
			Ves119												
			SugTrans15												
			SugTrans26												
			Transp225												
			MST6												
