# Supplementary material for: Imbalanced Regulation of Fungal Nutrient Transports According to Phosphate Availability in a Symbiocosm Formed by Poplar, Sorghum, and Rhizophagus irregularis
Source: Front Plant Sci. 2019 Dec 12;10:1617. doi: 10.3389/fpls.2019.01617 (PMC6920215; doi:10.3389/fpls.2019.01617)
Supplement: Table S6 — Relative abundances of metabolites detected in the ERM of R. irregularis. Data was log transformed and tested by Wilcoxon rank sum test (p<0.05) in MeV v4.9 (http://www.tm4.org/mev.html). Significant p-values are highlighted in bold. [file Table_6.pdf]

Class	Name	Average abundance of metabolites in poplar				Standard deviation				log2 ratios					2-way ANOVA		
		lP +AM	hP +AM	lP -AM	hP -AM	lP +AM	hP +AM	lP -AM	hP -AM	lP+AM vs hP+AM	lP-AM vs hP -AM	log2ratio lP vs lP ctr	hP +AM vs hP-AM	+AM vs -AM	Effect of mycorrhization	Effect of p-availability	Effect of interaction
Acids	Aconitic acid, cis-	0,001	0,001	0,003	0,004	0,000	0,000	0,001	0,002	0,0	-0,2	-1,5	-1,7	-1,6	**0,006**	0,863	0,934
Acids	Benzoic acid	0,009	0,010	0,019	0,011	0,001	0,001	0,005	0,002	-0,2	0,7	-1,0	-0,1	-0,6	**0,013**	0,182	**0,034**
Acids	Benzoic acid, 3,4-dihydroxy-	0,017	0,018	0,020	0,018	0,007	0,005	0,004	0,009	-0,1	0,1	-0,3	0,0	-0,2	0,750	0,839	0,602
Acids	Benzoic acid, 4-hydroxy-	0,017	0,019	0,022	0,012	0,008	0,003	0,003	0,005	-0,1	0,8	-0,3	0,6	0,1	0,767	0,426	0,162
Acids	Citric acid	0,026	0,025	0,023	0,014	0,004	0,002	0,002	0,007	0,1	0,6	0,2	0,8	0,5	0,054	0,125	0,172
Acids	Fumaric acid	0,011	0,009	0,021	0,016	0,004	0,003	0,003	0,007	0,2	0,3	-0,9	-0,8	-0,9	**0,038**	0,325	0,814
Acids	Glutaric acid, 2-hydroxy-	0,005	0,006	0,021	0,014	0,002	0,004	0,004	0,004	-0,3	0,6	-2,0	-1,1	-1,6	**0,003**	0,546	0,420
Acids	Glutaric acid, 2-oxo-	0,004	0,004	0,020	0,006	0,002	0,002	0,005	0,001	0,2	1,6	-2,2	-0,8	-1,7	**0,009**	0,111	0,180
Acids	Glutaric acid, 3-hydroxy-3-methyl-	0,008	0,011	0,022	0,013	0,004	0,005	0,003	0,005	-0,4	0,8	-1,4	-0,2	-0,9	0,065	0,549	0,199
Acids	Isocitric acid	0,024	0,021	0,020	0,011	0,006	0,004	0,002	0,006	0,2	0,9	0,3	1,0	0,6	0,054	0,084	0,190
Acids	Lactic acid	0,005	0,009	0,017	0,007	0,002	0,002	0,006	0,002	-0,7	1,4	-1,6	0,4	-0,7	0,083	0,389	**0,013**
Acids	Malic acid	0,016	0,019	0,021	0,013	0,006	0,007	0,002	0,001	-0,2	0,7	-0,4	0,6	0,1	0,958	0,412	0,135
Acids	Malic acid, 2-isopropyl-	ND	ND	0,018	0,009	ND	ND	0,005	0,001	ND	1,0	ND	ND	ND	ND	ND	ND
Acids	Malic acid, 2-methyl-	ND	ND	0,021	0,012	ND	ND	0,004	0,002	ND	0,8	ND	ND	ND	ND	ND	ND
Acids	Pyruvic acid	0,021	0,021	0,019	0,015	0,005	0,002	0,002	0,004	0,0	0,3	0,2	0,4	0,3	0,147	0,400	0,454
Acids	Quinic acid	0,003	0,005	0,005	0,003	0,001	0,001	0,002	0,002	-0,8	0,9	-1,0	0,7	-0,1	0,895	0,723	0,092
Acids	Shikimic acid	0,002	0,003	0,016	0,009	0,000	0,001	0,001	0,002	-0,7	0,8	-3,1	-1,5	-2,3	**0,000**	0,658	**0,008**
Acids	Succinic acid	0,008	0,008	0,018	0,011	0,002	0,005	0,003	0,002	0,0	0,7	-1,1	-0,5	-0,8	**0,032**	0,212	0,552
Acids	Vanillic acid	0,015	0,014	0,021	0,015	0,005	0,005	0,003	0,004	0,2	0,5	-0,4	-0,1	-0,3	0,353	0,258	0,631
Alcohols	Benzylalcohol	0,009	0,010	0,016	0,008	0,002	0,001	0,007	0,003	-0,1	1,0	-0,8	0,2	-0,4	0,479	0,180	0,104
Aliphatic	Heneicosane, n-	0,013	0,015	0,013	0,012	0,003	0,001	0,001	0,004	-0,1	0,2	0,1	0,3	0,2	0,372	0,855	0,419
Aliphatic	Tetracosane, n-	0,001	0,001	0,001	0,001	0,000	0,000	0,000	0,000	-0,2	0,2	0,3	0,7	0,5	0,140	0,778	0,471
Aliphatic	Triacontane, n-	0,001	0,001	0,001	0,001	0,000	0,000	0,000	0,000	0,0	-0,1	0,5	0,4	0,5	0,098	0,985	0,968
Amino Acids	Aspartic acid	0,001	0,000	0,019	0,012	0,000	0,000	0,006	0,004	0,8	0,6	-4,4	-4,7	-4,5	**0,000**	0,134	0,845
Amino Acids	Butanoic acid, 4-amino-	0,001	0,001	0,007	0,005	0,000	0,000	0,002	0,000	0,7	0,5	-3,0	-3,1	-3,0	**0,000**	**0,032**	0,572
Amino Acids	Glutamic acid	0,002	0,001	0,009	0,017	0,001	0,001	0,002	0,004	1,3	-0,9	-1,9	-4,0	-2,9	**0,001**	0,574	0,090
Amino Acids	Glycine	0,006	0,004	0,011	0,007	0,001	0,001	0,003	0,002	0,4	0,6	-1,0	-0,8	-0,9	**0,023**	0,112	0,736
Amino Acids	Isoleucine	0,005	0,004	0,018	0,012	0,001	0,001	0,005	0,001	0,5	0,7	-1,8	-1,7	-1,8	**0,000**	**0,041**	0,873
Amino Acids	Leucine	0,007	0,007	0,019	0,015	0,001	0,001	0,003	0,006	-0,1	0,3	-1,4	-1,0	-1,2	**0,001**	0,483	0,306
Amino Acids	Phenylalanine	0,002	0,001	0,019	0,012	0,001	0,000	0,005	0,003	0,8	0,7	-3,5	-3,6	-3,5	**0,000**	**0,048**	0,964
Amino Acids	Pyroglutamic acid	0,006	0,005	0,014	0,015	0,001	0,001	0,002	0,007	0,2	0,0	-1,3	-1,6	-1,4	**0,001**	0,532	0,762
Amino Acids	Serine	0,008	0,006	0,017	0,012	0,001	0,001	0,004	0,001	0,5	0,6	-1,1	-1,0	-1,1	**0,000**	**0,014**	0,765
Amino Acids	Valine	0,003	0,003	0,018	0,011	0,001	0,001	0,006	0,000	-0,1	0,8	-2,7	-1,8	-2,3	**0,000**	0,224	0,118
Aromatic	Catechol	0,004	0,001	0,011	0,003	0,004	0,000	0,010	0,002	1,4	1,9	-1,4	-1,0	-1,3	0,245	0,276	0,598
Fatty Acids	Dodecanoic acid	0,011	0,012	0,011	0,011	0,002	0,003	0,003	0,001	-0,1	0,1	0,1	0,2	0,1	0,571	0,879	0,789
Fatty Acids	Hexadecanoic acid	0,018	0,019	0,020	0,017	0,002	0,002	0,005	0,005	-0,1	0,3	-0,1	0,2	0,0	0,770	0,643	0,431
Fatty Acids	Hexanoic acid	0,017	0,015	0,013	0,017	0,007	0,002	0,002	0,006	0,1	-0,4	0,3	-0,2	0,1	0,812	0,746	0,622
Fatty Acids	Octadecanoic acid	0,017	0,017	0,017	0,014	0,003	0,003	0,006	0,004	0,0	0,3	0,0	0,3	0,1	0,516	0,539	0,622
N- Compounds	Ethanolamine	0,016	0,018	0,017	0,017	0,001	0,001	0,003	0,005	-0,2	0,0	-0,1	0,0	-0,1	0,927	0,722	0,594
N- Compounds	Phenol, 2-amino-	0,006	0,005	0,016	0,009	0,003	0,001	0,004	0,004	0,3	0,9	-1,5	-0,9	-1,3	**0,019**	0,242	0,306
N- Compounds	Putrescine	0,023	0,023	0,022	0,019	0,003	0,004	0,001	0,005	0,0	0,2	0,1	0,3	0,2	0,362	0,499	0,616
N- Compounds	Pyridine, 2-hydroxy-	0,017	0,021	0,023	0,018	0,004	0,004	0,001	0,006	-0,3	0,3	-0,4	0,3	-0,1	0,803	0,872	0,161
Phenylpropanoids	Caffeic acid, cis-	0,016	0,016	0,019	0,010	0,003	0,002	0,002	0,004	0,0	0,9	-0,2	0,7	0,2	0,298	0,070	0,075
Phenylpropanoids	Caffeic acid, trans-	0,015	0,014	0,020	0,009	0,003	0,002	0,002	0,004	0,1	1,1	-0,5	0,6	0,0	0,666	0,056	0,070
Phenylpropanoids	Cinnamic acid, 4-hydroxy-, trans-	ND	ND	0,012	0,008	ND	ND	0,000	0,003	ND	0,6	ND	-0,5	ND	ND	ND	ND
Phenylpropanoids	Epicatechin	0,014	0,016	0,020	0,013	0,003	0,004	0,003	0,004	-0,2	0,6	-0,5	0,3	-0,1	0,668	0,323	0,138
Phenylpropanoids	Ferulic acid, trans-	0,015	0,014	0,021	0,011	0,004	0,004	0,000	0,001	0,1	0,9	-0,5	0,4	-0,1	0,703	0,066	0,121
Phenylpropanoids	Quinic acid, 3-caffeoyl-, cis-	0,018	0,016	0,009	0,008	0,009	0,002	0,003	0,007	0,2	0,1	1,1	1,0	1,0	0,102	0,745	0,572
Phenylpropanoids	Quinic acid, 3-caffeoyl-, trans-	0,016	0,015	0,010	0,007	0,009	0,002	0,001	0,007	0,1	0,4	0,7	1,0	0,8	0,155	0,487	0,347
Phosphates	Fructose-6-phosphate	0,004	0,006	0,016	0,019	0,001	0,002	0,005	0,006	-0,5	-0,2	-2,0	-1,7	-1,9	**0,003**	0,420	0,806
Phosphates	Glucose-6-phosphate	0,002	0,005	0,013	0,018	0,001	0,002	0,005	0,005	-1,1	-0,4	-2,6	-2,0	-2,2	**0,000**	0,065	0,453
Phosphates	myo-Inositol-phosphate	ND	ND	0,001	0,003	ND	ND	0,000	0,002	ND	-1,1	-1,2	ND	ND	ND	ND	ND
Phosphates	Phosphoric acid	0,001	0,000	0,002	0,011	0,000	0,000	0,000	0,014	0,4	-2,9	-1,2	-4,5	-3,5	**0,042**	0,634	0,468
Phosphates	Phosphoric acid monomethyl ester	0,003	0,004	0,017	0,017	0,001	0,001	0,007	0,009	-0,4	0,0	-2,5	-2,2	-2,4	**0,001**	0,648	0,676
Polyhydroxy Acids	Arabinonic acid-1,4-lactone	0,013	0,009	0,019	0,006	0,001	0,002	0,005	0,001	0,5	1,6	-0,5	0,5	-0,2	0,914	**0,001**	**0,041**
Polyhydroxy Acids	Galactaric acid	0,006	0,007	0,020	0,011	0,002	0,003	0,005	0,002	-0,2	0,8	-1,7	-0,6	-1,2	**0,011**	0,455	0,209
Polyhydroxy Acids	Galactonic acid	0,004	0,006	0,017	0,011	0,002	0,003	0,001	0,002	-0,6	0,6	-2,1	-0,8	-1,4	**0,002**	0,963	0,100
Polyhydroxy Acids	Gluconic acid	0,002	0,002	0,007	0,003	0,001	0,001	0,001	0,000	-0,2	1,2	-1,7	-0,3	-1,1	**0,018**	0,217	0,139
Polyhydroxy Acids	Glyceric acid	0,006	0,007	0,020	0,018	0,001	0,001	0,002	0,005	-0,3	0,2	-1,9	-1,4	-1,6	**0,000**	0,900	0,238
Polyhydroxy Acids	Gulonic acid	0,014	0,013	0,021	0,013	0,002	0,002	0,003	0,006	0,0	0,6	-0,6	0,0	-0,4	0,425	0,257	0,311
Polyhydroxy Acids	Lyxonic acid-1,4-lactone	0,015	0,012	0,019	0,010	0,001	0,002	0,005	0,006	0,3	0,9	-0,3	0,3	-0,1	0,772	0,080	0,292
Polyhydroxy Acids	Ribonic acid	0,009	0,007	0,020	0,014	0,002	0,000	0,003	0,004	0,4	0,5	-1,2	-1,1	-1,1	**0,001**	**0,048**	0,686
Polyhydroxy Acids	Saccharic acid	0,004	0,004	0,021	0,008	0,002	0,001	0,004	0,002	0,0	1,4	-2,3	-0,9	-1,8	**0,001**	0,065	**0,039**
Polyhydroxy Acids	Threonic acid	0,004	0,003	0,019	0,009	0,001	0,001	0,003	0,004	0,5	1,1	-2,2	-1,7	-2,0	**0,000**	**0,019**	0,292
Polyols	Arabitol	0,001	0,001	0,002	0,002	0,000	0,000	0,000	0,001	-0,2	0,3	-1,4	-1,0	-1,2	**0,004**	0,722	0,471
Polyols	Galactitol	0,017	0,011	0,013	0,018	0,005	0,002	0,001	0,005	0,5	-0,5	0,3	-0,7	-0,2	0,507	0,853	0,143
Polyols	Glycerol	0,006	0,011	0,007	0,008	0,000	0,006	0,002	0,002	-0,9	-0,1	-0,3	0,5	0,2	0,927	0,307	0,475
Polyols	Inositol, myo-	0,003	0,002	0,019	0,016	0,001	0,000	0,005	0,006	1,0	0,3	-2,6	-3,3	-2,8	**0,000**	0,123	0,422
Polyols	Mannitol	0,001	0,001	0,001	0,001	0,000	0,000	0,000	0,001	0,3	-0,9	0,4	-0,7	-0,2	0,896	0,895	0,468
Sugar Conjugates	Galactinol	0,003	0,002	0,008	0,016	0,001	0,000	0,002	0,012	0,7	-1,0	-1,4	-3,1	-2,3	**0,010**	0,829	0,391
Sugar Conjugates	Salicin	0,006	0,004	0,014	0,005	0,005	0,001	0,008	0,002	0,7	1,6	-1,2	-0,3	-0,9	0,193	0,137	0,275
Sugar Conjugates	Salicylic acid-glucopyranoside	0,002	0,002	0,006	0,012	0,000	0,000	0,000	0,013	-0,1	-1,1	-1,8	-2,9	-2,5	**0,038**	0,913	0,998
Sugars	Arabinose	0,006	0,007	0,017	0,013	0,002	0,004	0,005	0,002	-0,2	0,4	-1,5	-0,9	-1,2	**0,008**	0,776	0,575
Sugars	Fructose	0,018	0,014	0,010	0,010	0,009	0,007	0,006	0,006	0,3	0,0	0,8	0,5	0,6	0,300	0,769	0,986
Sugars	Galactose	0,011	0,015	0,014	0,009	0,005	0,012	0,007	0,001	-0,4	0,6	-0,3	0,7	0,2	0,984	0,813	0,615
Sugars	Glucose	0,009	0,008	0,007	0,008	0,005	0,005	0,003	0,005	0,2	-0,2	0,4	-0,1	0,2	0,728	0,791	0,941
Sugars	Glucose, 1,6-anhydro-, beta-	0,009	0,010	0,020	0,012	0,001	0,001	0,004	0,001	-0,1	0,7	-1,1	-0,3	-0,8	**0,001**	0,076	**0,018**
Sugars	Maltose	0,004	0,003	0,013	0,007	0,001	0,001	0,007	0,002	0,4	0,9	-1,6	-1,1	-1,4	**0,006**	0,139	0,622
Sugars	Mannose	0,008	0,012	0,010	0,010	0,003	0,011	0,004	0,004	-0,7	0,0	-0,3	0,3	0,0	0,800	0,791	0,866
Sugars	Raffinose	0,003	0,001	0,001	0,011	0,001	0,000	0,000	0,014	1,2	-4,1	2,1	-3,3	-1,6	0,602	0,766	0,173
Sugars	Rhamnose	0,007	0,007	0,016	0,012	0,003	0,001	0,004	0,000	0,0	0,5	-1,2	-0,7	-1,0	**0,009**	0,674	0,351
Sugars	Ribose	0,004	0,003	0,017	0,009	0,001	0,001	0,006	0,002	0,1	0,9	-2,3	-1,5	-2,0	**0,001**	0,258	0,258
Sugars	Sucrose	0,023	0,024	0,023	0,021	0,002	0,004	0,002	0,007	0,0	0,1	0,0	0,2	0,1	0,534	0,669	0,658
Sugars	Trehalose, alpha,alpha'-	0,002	0,003	0,001	0,001	0,000	0,001	0,000	0,001	-0,5	-0,2	1,0	1,3	1,2	**0,046**	0,883	0,841
Sugars	Xylose	0,007	0,008	0,014	0,007	0,003	0,004	0,007	0,004	-0,1	0,9	-1,0	0,1	-0,5	0,639	0,526	0,363
Sugars	Xylulose	0,001	ND	0,006	0,003	ND	ND	0,002	0,001	ND	1,1	-2,4	ND	ND	ND	ND	ND
**Unknown Metabolites**																	
MSTs	Unknown#bth-pae-007	0,005	0,005	0,003	0,003	0,002	0,000	0,001	0,001	0,1	-0,1	0,8	0,6	0,7	0,056	0,964	0,918
MSTs	A112003-101	0,022	0,020	0,017	0,015	0,004	0,003	0,001	0,004	0,1	0,2	0,4	0,4	0,4	0,084	0,402	0,773
MSTs	Unknown#bth-pae-013	0,016	0,011	0,017	0,018	0,006	0,001	0,002	0,009	0,6	-0,1	-0,1	-0,7	-0,4	0,285	0,454	0,641
MSTs	Unknown#bth-pae-067	0,002	0,003	0,020	0,016	0,001	0,000	0,005	0,006	-0,6	0,3	-3,6	-2,7	-3,1	**0,000**	0,592	0,150
MSTs	D118786	0,024	0,028	0,022	0,020	0,004	0,002	0,002	0,006	-0,2	0,1	0,1	0,5	0,3	0,138	0,960	0,327
MSTs	A124002-101	0,024	0,015	0,018	0,007	0,007	0,001	0,002	0,003	0,6	1,3	0,4	1,1	0,6	**0,037**	**0,010**	0,169
MSTs	A140003-101	ND	ND	0,019	0,012	ND	ND	0,003	0,002	ND	0,7	ND	ND	ND	ND	ND	ND
MSTs	A141003-101	ND	ND	0,020	0,013	ND	ND	0,004	0,002	ND	0,6	-5,5	ND	ND	ND	ND	ND
MSTs	Unknown#bth-pae-028	0,011	0,011	0,011	0,008	0,002	0,002	0,002	0,003	0,0	0,5	0,0	0,5	0,2	0,293	0,309	0,265
MSTs	A143003-101	0,025	0,027	0,021	0,020	0,002	0,003	0,002	0,006	-0,1	0,1	0,2	0,4	0,3	0,110	0,951	0,547
MSTs	A145005-101	0,025	0,026	0,022	0,020	0,003	0,003	0,002	0,006	-0,1	0,1	0,2	0,4	0,3	0,159	0,719	0,524
MSTs	A145008-101	0,009	0,005	0,005	0,005	0,003	0,002	0,002	0,002	0,6	-0,1	0,9	0,2	0,5	0,198	0,511	0,340
MSTs	NA145015	0,025	0,027	0,022	0,020	0,002	0,002	0,003	0,007	-0,1	0,1	0,2	0,4	0,3	0,135	0,877	0,515
MSTs	A147001-101	0,025	0,025	0,021	0,018	0,002	0,002	0,004	0,006	0,0	0,2	0,2	0,5	0,4	0,095	0,536	0,510
MSTs	A148010-101	0,006	0,005	0,017	0,013	0,002	0,001	0,005	0,009	0,3	0,4	-1,4	-1,3	-1,4	**0,014**	0,242	0,671
MSTs	Unknown#bth-pae-039	ND	ND	0,023	0,014	ND	ND	0,001	0,006	ND	0,7	ND	ND	ND	ND	ND	ND
MSTs	NA160001	0,001	0,001	0,017	0,009	0,000	0,000	0,001	0,002	-0,7	1,0	-4,4	-2,7	-3,6	**0,000**	0,441	**0,009**
MSTs	NA170001	0,009	0,013	0,020	0,010	0,003	0,004	0,004	0,001	-0,5	1,1	-1,2	0,4	-0,4	0,152	0,426	**0,025**
MSTs	A175008-101	0,002	0,003	0,009	0,007	0,001	0,000	0,003	0,005	-0,9	0,3	-2,5	-1,3	-1,9	NaN	NaN	NaN
MSTs	NA176001	0,016	0,013	0,024	0,015	0,006	0,004	0,002	0,006	0,3	0,6	-0,6	-0,3	-0,5	0,203	0,182	0,491
MSTs	similar to Fructose Derivate	0,014	0,018	0,021	0,010	0,009	0,011	0,005	0,007	-0,4	1,0	-0,6	0,8	0,0	0,825	0,618	0,298
MSTs	similar to Fructose Derivate	0,008	0,011	0,017	0,010	0,002	0,000	0,003	0,003	-0,4	0,7	-1,1	0,1	-0,6	0,095	0,584	**0,049**
MSTs	similar to Fructose Derivate	0,014	0,017	0,011	0,006	0,010	0,012	0,003	0,004	-0,3	1,0	0,3	1,6	0,9	0,625	0,715	0,345
MSTs	A186003-101	0,011	0,009	0,017	0,004	0,002	0,003	0,006	0,001	0,3	2,2	-0,7	1,2	-0,1	0,410	**0,005**	**0,025**
MSTs	A190021-101	0,006	0,004	0,005	0,007	0,003	0,000	0,003	0,003	0,5	-0,4	0,0	-0,9	-0,4	NaN	NaN	NaN
MSTs	A196004-101	0,008	0,004	0,009	0,013	0,003	0,001	0,002	0,003	0,9	-0,5	-0,2	-1,6	-0,9	**0,014**	0,721	0,077
MSTs	NA201002	0,003	0,003	0,021	0,006	0,000	0,000	0,004	0,002	0,0	1,7	-2,9	-1,2	-2,3	**0,000**	**0,016**	**0,018**
MSTs	A203003-101	0,009	0,006	0,011	0,011	0,003	0,001	0,003	0,004	0,6	0,0	-0,3	-0,9	-0,6	0,140	0,464	0,583
MSTs	A203005-101	0,006	0,003	0,012	0,010	0,003	0,000	0,003	0,010	1,0	0,2	-1,1	-1,9	-1,4	NaN	NaN	NaN
MSTs	A202007-101	0,009	0,004	0,009	0,011	0,004	0,002	0,003	0,002	1,1	-0,3	-0,1	-1,5	-0,7	0,061	0,390	0,101
MSTs	A204012-101	0,008	0,006	0,022	0,007	0,003	0,002	0,003	0,004	0,5	1,7	-1,4	-0,2	-1,0	0,100	**0,015**	0,093
MSTs	A206002-101	0,004	0,004	0,018	0,009	0,001	0,000	0,006	0,003	0,2	1,1	-2,1	-1,3	-1,8	**0,000**	**0,037**	0,132
MSTs	A207008-101	0,002	0,003	0,003	0,002	0,000	0,000	0,001	0,001	-0,4	0,6	-0,4	0,6	0,1	0,622	0,792	**0,048**
MSTs	NA211001	0,006	0,008	0,018	0,007	0,003	0,002	0,006	0,003	-0,4	1,5	-1,6	0,2	-0,9	0,205	0,290	0,056
MSTs	A212001-101	0,012	0,009	0,015	0,018	0,005	0,004	0,002	0,009	0,4	-0,3	-0,4	-1,0	-0,7	0,158	0,741	0,632
MSTs	NA213001	0,012	0,012	0,010	0,014	0,006	0,005	0,005	0,002	0,0	-0,4	0,2	-0,2	0,0	0,811	0,516	0,625
MSTs	A214003-101	0,009	0,011	0,016	0,010	0,003	0,004	0,007	0,002	-0,3	0,7	-0,9	0,2	-0,4	0,383	0,772	0,212
MSTs	A228001-101	0,016	0,015	0,020	0,011	0,007	0,004	0,004	0,004	0,1	0,8	-0,3	0,4	0,0	0,962	0,268	0,293
MSTs	D226501	0,022	0,020	0,018	0,012	0,005	0,000	0,003	0,003	0,1	0,6	0,3	0,7	0,5	0,082	0,184	0,317
MSTs	A230002-101	0,011	0,007	0,007	0,003	0,005	0,002	0,000	0,001	0,6	1,2	0,8	1,4	1,0	**0,027**	**0,046**	0,275
MSTs	similar to Glycerolaldopyranosid	0,021	0,015	0,014	0,005	0,008	0,002	0,001	0,002	0,5	1,5	0,6	1,5	0,9	**0,011**	**0,014**	0,108
MSTs	A237001-101	0,022	0,012	0,006	0,001	0,009	0,003	0,000	0,001	0,9	2,2	1,7	3,1	2,1	**0,000**	**0,005**	0,074
MSTs	A237002-101	0,015	0,016	0,019	0,011	0,007	0,004	0,005	0,001	-0,1	0,8	-0,3	0,5	0,0	0,968	0,527	0,188
MSTs	A241003-101	0,002	0,004	0,019	0,007	0,000	0,002	0,006	0,003	-1,0	1,5	-3,4	-0,9	-2,2	**0,001**	0,342	**0,028**
MSTs	D255515	0,002	0,005	0,017	0,015	0,000	0,004	0,005	0,007	-1,5	0,2	-3,2	-1,5	-2,2	**0,001**	0,429	0,189
MSTs	D262250	0,003	0,002	0,009	0,012	0,001	0,000	0,003	0,010	0,5	-0,3	-1,5	-2,3	-1,9	**0,030**	0,595	0,914
MSTs	A259008-101	0,007	0,005	0,013	0,005	0,001	0,001	0,009	0,002	0,4	1,3	-1,0	-0,1	-0,7	0,344	0,069	0,373
MSTs	D260482	0,022	0,023	0,022	0,019	0,003	0,002	0,003	0,005	-0,1	0,2	0,0	0,3	0,1	0,447	0,705	0,396
MSTs	A262001-101	0,001	0,002	0,001	0,011	0,001	0,000	0,000	0,014	-0,2	-3,6	0,6	-2,8	-2,0	0,915	0,543	0,828
MSTs	A267001-101	0,011	0,011	0,017	0,013	0,001	0,002	0,007	0,002	0,0	0,4	-0,6	-0,3	-0,4	0,164	0,569	0,680
MSTs	A275003-101	0,005	0,005	0,011	0,013	0,001	0,002	0,001	0,008	0,1	-0,3	-0,9	-1,3	-1,1	**0,016**	0,968	0,758
MSTs	A276008-101	0,004	0,006	0,016	0,008	0,003	0,002	0,006	0,002	-0,4	0,9	-1,8	-0,5	-1,2	**0,016**	0,762	0,121
MSTs	A279007-101	0,018	0,019	0,019	0,017	0,006	0,004	0,003	0,005	-0,1	0,2	-0,1	0,2	0,0	0,927	0,883	0,555
MSTs	D278931	0,023	0,024	0,020	0,021	0,003	0,003	0,001	0,007	0,0	-0,1	0,2	0,2	0,2	0,303	0,999	0,953
MSTs	A284009-101	0,000	0,000	0,000	0,000	0,000	0,000	0,000	0,000	-0,2	0,8	-0,4	0,6	0,1	0,571	0,440	0,193
MSTs	A288003-101	0,009	0,008	0,018	0,012	0,001	0,001	0,006	0,003	0,3	0,6	-0,9	-0,6	-0,8	**0,018**	0,127	0,576
MSTs	A288004-101	0,010	0,009	0,017	0,016	0,003	0,003	0,002	0,010	0,2	0,0	-0,7	-0,9	-0,8	0,108	0,511	0,981
MSTs	A295006-101	0,016	0,017	0,003	0,001	0,007	0,011	0,000	0,000	0,0	1,0	2,7	3,7	3,1	**0,000**	0,289	0,386
MSTs	A295012-101	0,019	0,017	0,019	0,017	0,004	0,005	0,005	0,008	0,1	0,1	0,0	0,0	0,0	0,884	0,581	0,883
MSTs	A302001-101	0,006	0,010	0,022	0,016	0,002	0,005	0,004	0,008	-0,7	0,4	-1,9	-0,7	-1,3	**0,012**	0,988	0,199
MSTs	A306001-101	0,012	0,011	0,015	0,017	0,005	0,004	0,002	0,010	0,1	-0,1	-0,3	-0,6	-0,5	0,339	0,809	0,967
MSTs	A311002-101	0,023	0,014	0,015	0,011	0,003	0,003	0,004	0,008	0,7	0,5	0,6	0,4	0,5	0,116	0,096	0,847
