# Supplementary material for: Imbalanced Regulation of Fungal Nutrient Transports According to Phosphate Availability in a Symbiocosm Formed by Poplar, Sorghum, and Rhizophagus irregularis
Source: Front Plant Sci. 2019 Dec 12;10:1617. doi: 10.3389/fpls.2019.01617 (PMC6920215; doi:10.3389/fpls.2019.01617)
Supplement: Supplementary file 8 [file DataSheet_1.pdf]

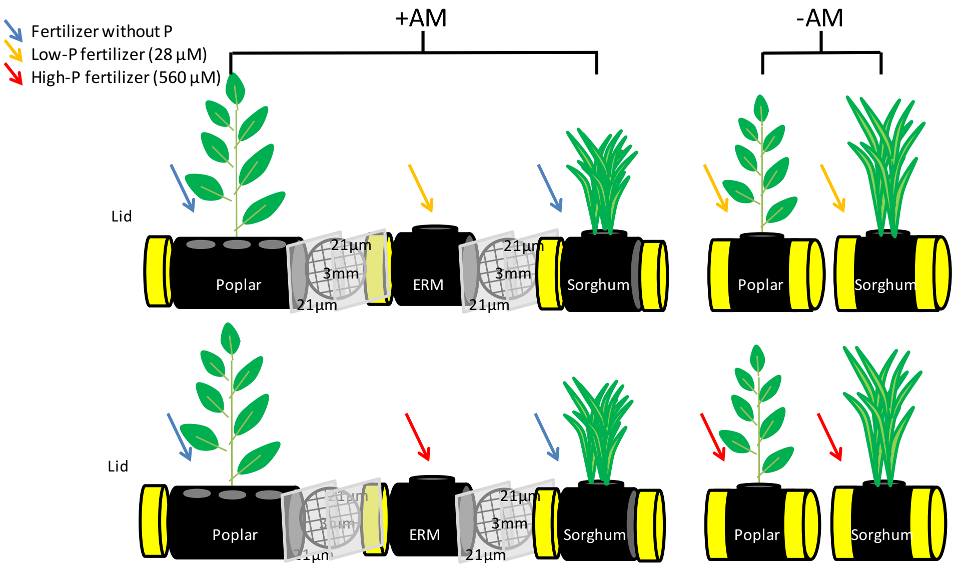

Figure S1 Symbiocosms. For a deeper understanding of symbiotic P transport microcosms with tripartite compartments (mycorrhizal treatment; +AM) or single compartments (non-mycorrhizal treatments; -AM) were set-up. All compartments contained a mixture of sand: zeolithe (1:1; w:w). In the mycorrhizal treatments a *P. trichocarpa* (poplar) cutting was planted in the middle compartment and *S. bicolor* (sorghum) seedlings were planted in the right compartment to create a common mycorrhizal network and to increase poplar root colonization. Both poplar and sorghum were inoculated with the AM fungus *R. irregularis* and were fertilized once a week with Hoagland solution without phosphorus (P). To obtain extraradical mycelium (ERM), P containing Hoagland solution (low-P, 28 µM; high-P, 560 µM) was applied in a third compartment where only the mycorrhizal fungus had access to it. In the non-mycorrhizal treatment poplar cuttings and sorghum seedling grew separately from each other in a single compartment. The control plants received the P containing fertilizer directly into their root compartment.

A.

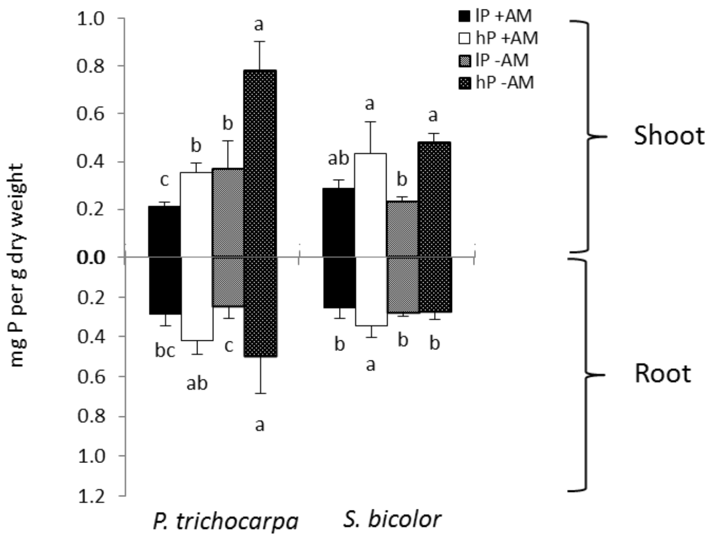

1.0
0.8
0.6
0.4
0.2
0.0
0.2
0.4
0.6
0.8
1.0
1.2
mg P per g dry weight
lP +AM
hP +AM
lP -AM
hP -AM
Shoot
Root
P. trichocarpa
S. bicolor

B.

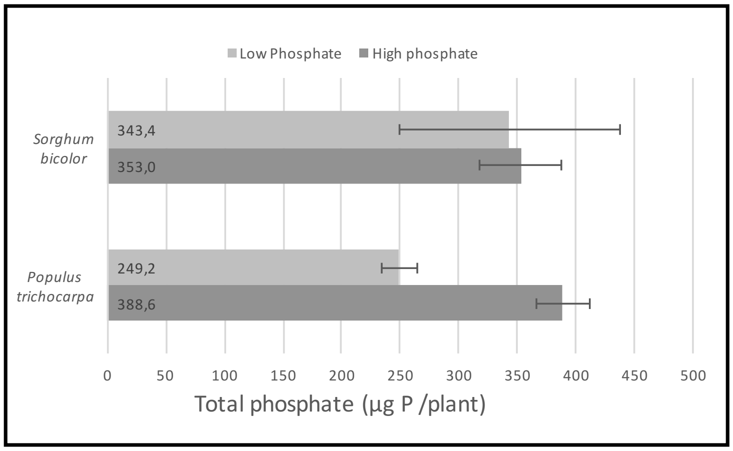

Low Phosphate
High phosphate
Sorghum bicolor
343,4
353,0
Populus trichocarpa
249,2
388,6
Total phosphate (µg P /plant)

C.

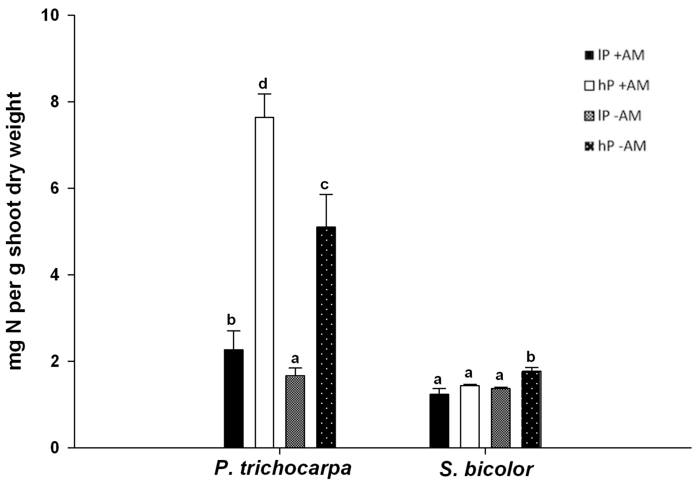

mg N per g shoot dry weight
lP +AM
hP +AM
lP -AM
hP -AM
P. trichocarpa
S. bicolor

Figure S2. P and N content in *P. trichocarpa* and *S. bicolor* plants

A. Shoots and roots P content of mycorrhizal (+AM) and non-mycorrhizal (-AM) *P. trichocarpa* and *S. bicolor* plants. P content was measured in low-P (lP) and high-P (hP) treatments. Values are means of six biological replicates. Differences between treatments were estimated by analysis of variance (ANOVA) per plant species and organ, followed by Tuckey honestly significant test (Tuckey HSD; $p<0.05$). Statistical differences are indicated by lower case letters.

B. Total P content of the mycorrhized plants. Data represent the mean value of both root and shoot content of the different mycorrhizal plants. Error bars represent the standard deviation (7 plants *per* condition)

C. Shoots N content of mycorrhizal (+AM) and non-mycorrhizal (-AM) *P. trichocarpa* and *S. bicolor* plants. N content was measured in low-P (lP) and high-P (hP) treatments. Values are means of six biological replicates. Differences between treatments were estimated by analysis of variance (ANOVA) per plant species and organ, followed by Tuckey honestly significant test (Tuckey HSD; $p<0.05$). Statistical differences are indicated by lower case letters.

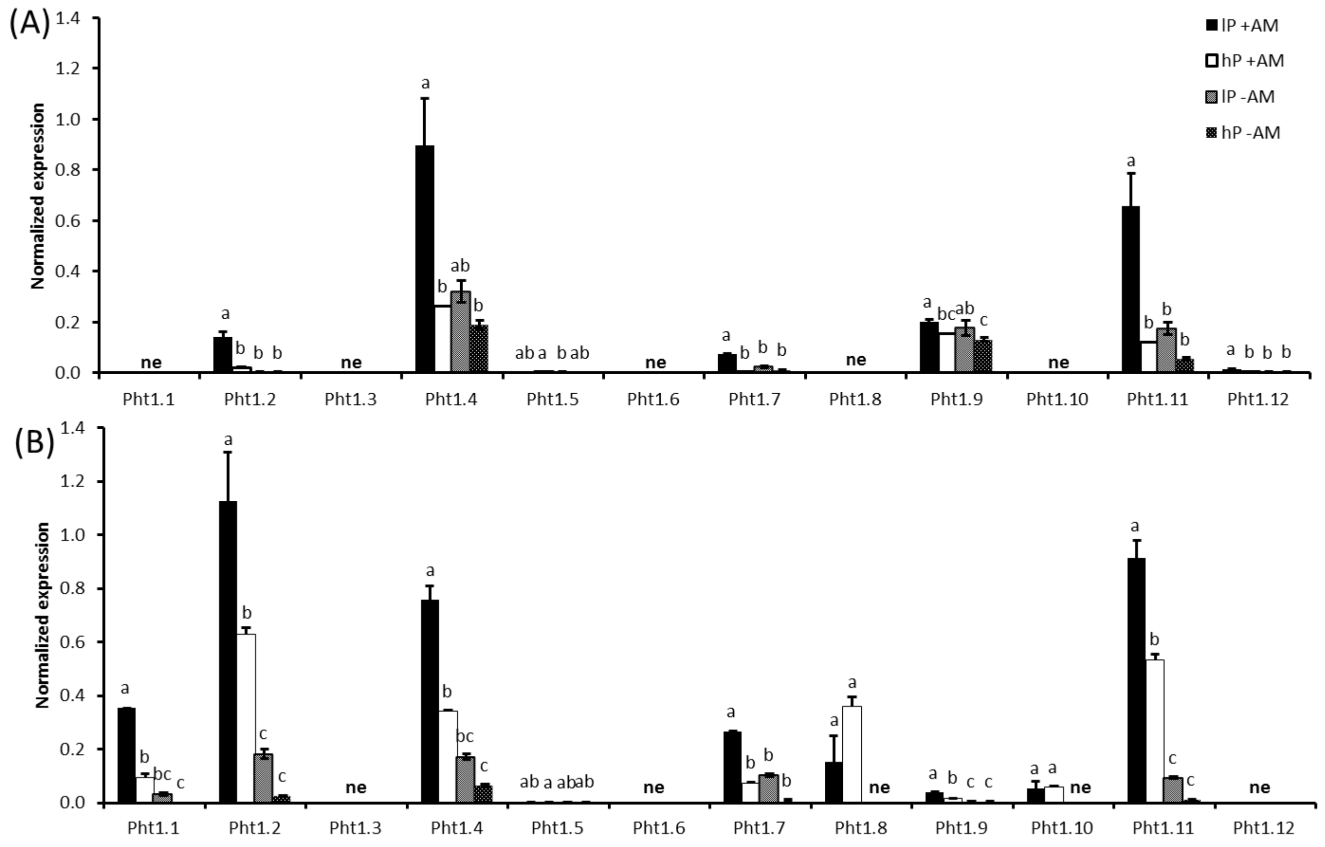

Figure S3. Quantification by qRT-PCR of the transcript abundances of Pht1 genes in *P. trichocarpa.* Quantification of transcript levels of the 12 Pht1 transporter genes in the shoots (A) and roots (B) in *P. trichocarpa* when mycorrhized (+AM) or not mycorrhized (-AM) with *R. irregularis* under low-P (lP) and high-P (hP) conditions. Transporter Pht1.3, Pht1.6 and Pht1.12 were not expressed; Pht1.8 and Pht1.10 only when the plant was mycorrhized and Pht1.1 was root specific. Values are the means of three biological and three technical replicates. Error bars represent the SE. Ubiquitin was used as reference transcript. Statistical analysis was performed by analysis of variance (ANOVA) for each gene, followed by Tuckey honest significant difference test (Tuckey HSD; $p<0.05$).

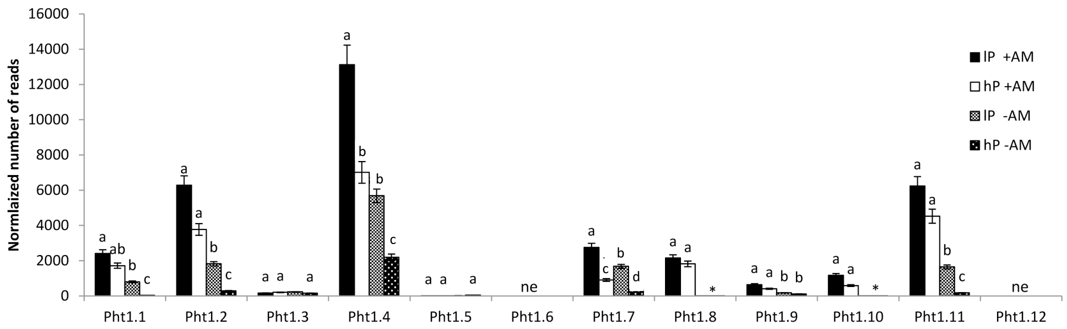

Figure S4 Quantification by mRNA – Sequencing (RNAseq) of the transcript abundances of Pht1 phosphate transporters in the roots of *P. trichocarpa.* Transcript abundances of the 12 Pht genes in *P. trichocarpa* were quantified in mycorrhized (+AM) and non-mycorrhized (-AM) roots in high-P (hP) and low-P (lP) treatment. PtrPht1.6 and PtrPht1.12 were not expressed (ne). Bar labeled with an asterisk had less than 10 reads and were considered as not expressed. Significant differences were estimated per gene using the Wald test. Lower case letters indicate significant differences in transcript abundances (p-value < 0.05).

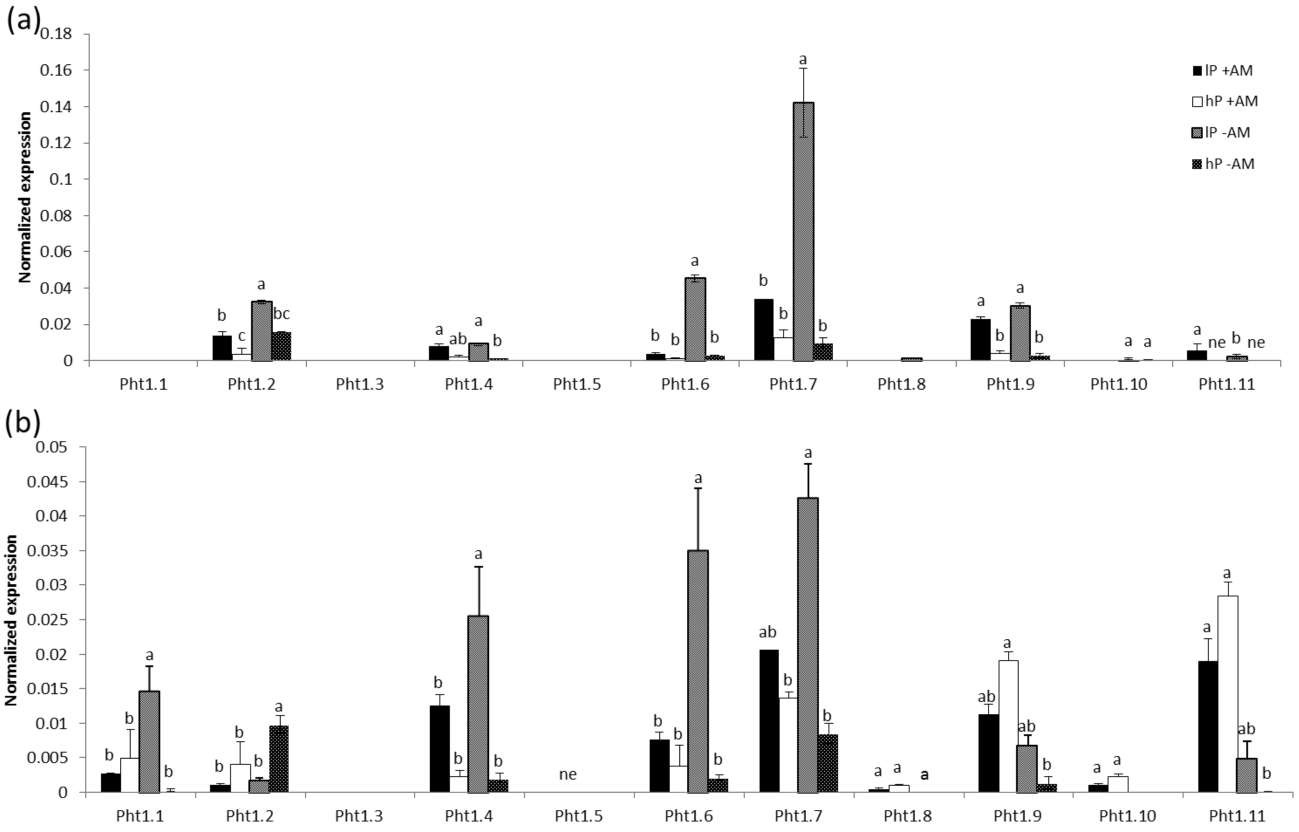

Figure S5 Quantification by qRT-PCR of the transcript abundances of Pht1 phosphate transporter genes in *S. bicolor*. Quantification of transcript levels of the 11 Pht1 transporter genes in the shoots (a) and roots (b) *S. bicolor* when mycorrhized (+AM) or not (-AM) by the AM fungus *R.irregularis* in low-P (lP) and high P (hP) conditions. Transporter Pht1.3 and Pht1.5 were not expressed; Pht1.10 only in roots when mycorrhized and only in shoots when plant was not mycorrhized and Pht1.1 was root specific. Values are the means of three biological and three technical replicates. Error bars represent the SE. Ubiquitin was used as reference transcript. Statistical analysis was performed by analysis of variance (ANOVA) for each gene, followed by Tuckey honest significant difference test (Tuckey HSD; $p<0.05$).

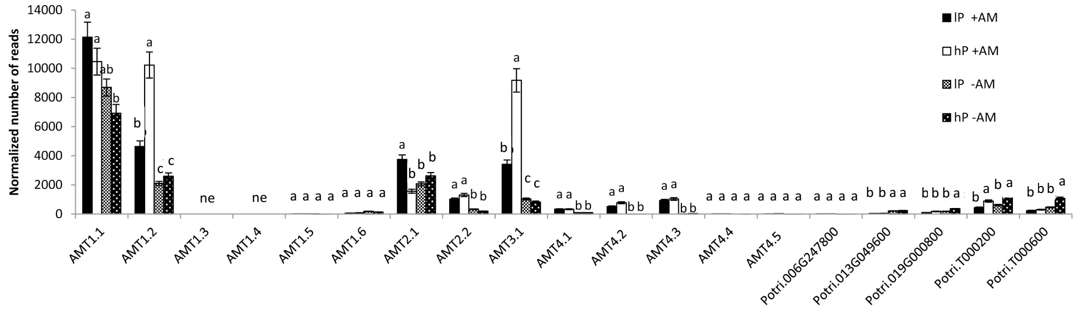

Figure S6. Quantification by mRNA – Sequencing (RNAseq) of the transcript abundances of ammonia transporters in the roots of *P. trichocarpa*. Transcript abundances of 19 AMT genes in *P. trichocarpa* when mycorrhized (+AM) or not (-AM) by the AM fungus *R.irregularis* in low-P (lP) and high-P (hP) conditions. Bars labeled with an asterisk had less than ten reads and were considered as not expressed. Significant differences were estimated per gene using the Wald test. Lower case letters indicate significant differences in transcript abundances (p-value < 0.05). Number of reads in the three biological replicates per condition were normalized per gene using DESeq2.

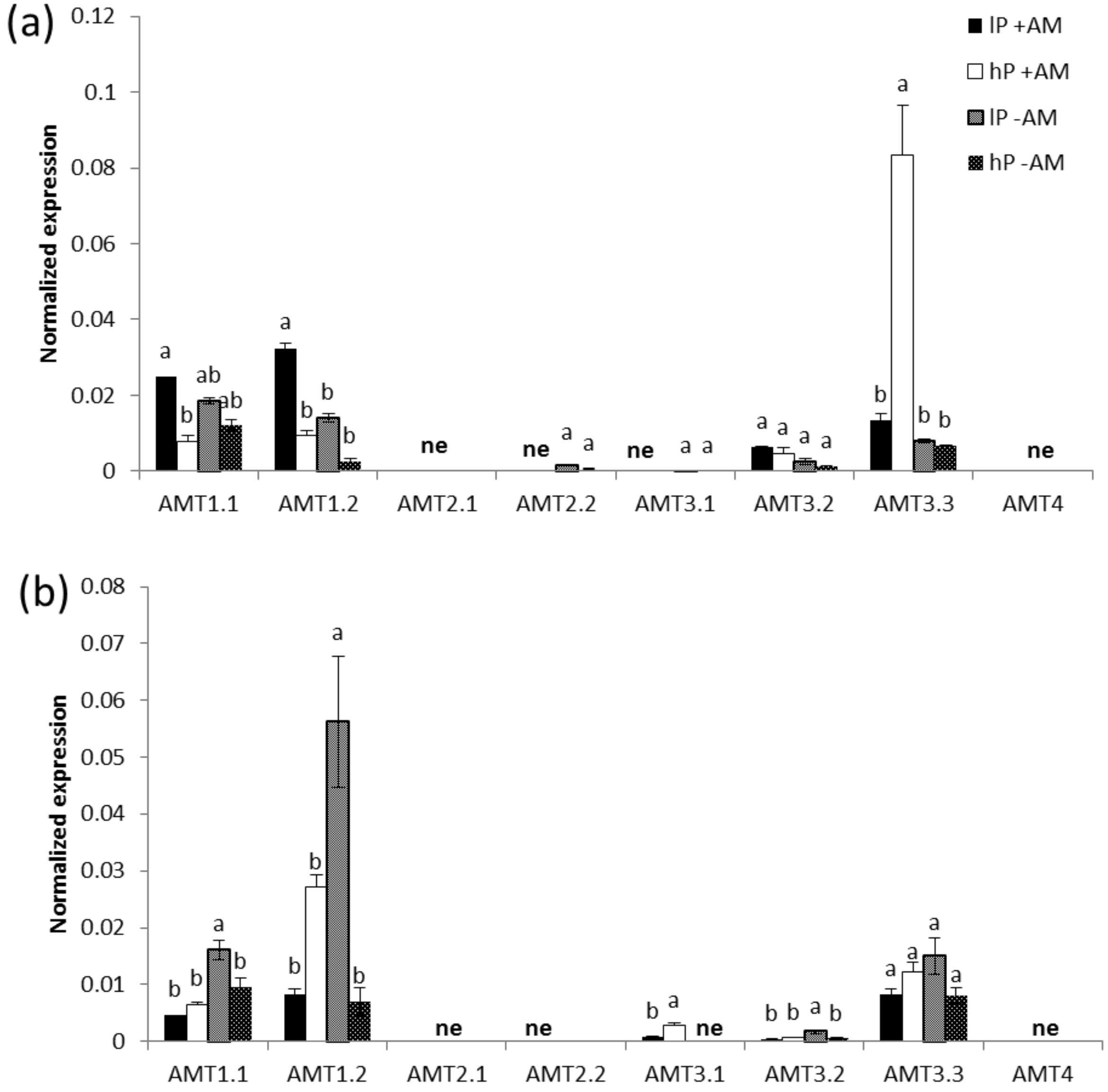

Figure S7 Quantification by qRT-PCR of the transcript abundances of the three ammonium transporters in the shoot (a) and root (b) of mycorrhized (+AM) and non-mycorrhized (-AM) *S. bicolor* under low P (lP) and high P (hP) condition. Values are the means of three biological replicates and three technical replicates, each. Error bars represent the SE. Ubiquitin was used as reference transcript. Statistical analysis was performed by analysis of variance (ANOVA) per gene, followed by Tuckey honest significant difference test (Tuckey HSD; $p<0.05$).

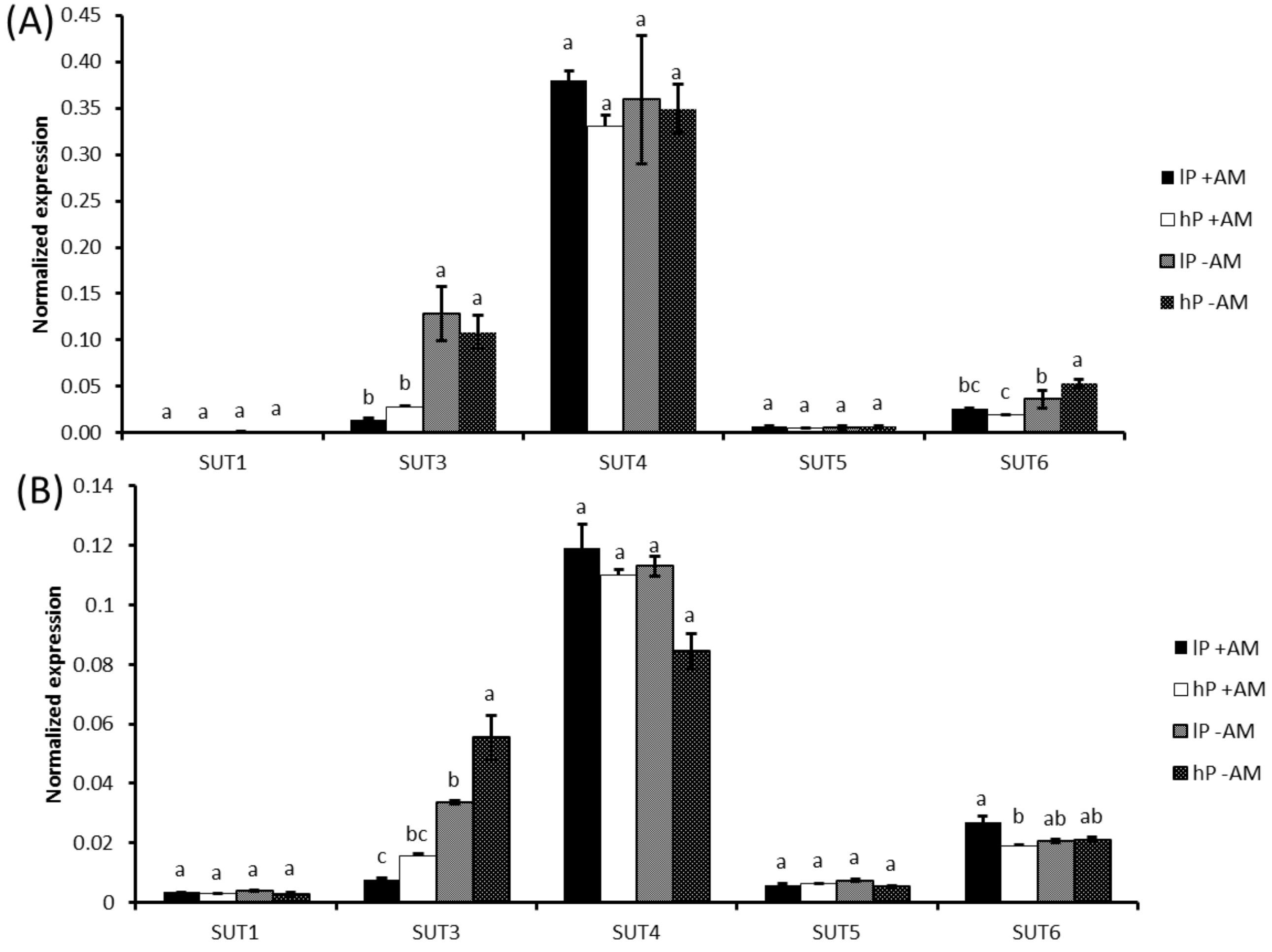

Figure S8. Quantification by qRT-PCR of the transcript abundances of five sugar transporters (SUT) in the shoots (A) and roots (B) of mycorrhized (+AM) and non-mycorrhized (-AM) *P. trichocarpa* under low P (lP) and high P (hP) condition. Values are the means of three biological replicates and three technical replicates, each. Error bars represent the SE. Ubiquitin was used as reference transcript. Statistical analysis was performed by analysis of variance (ANOVA) per gene, followed by Tuckey honest significant difference test (Tuckey HSD; $p<0.05$).

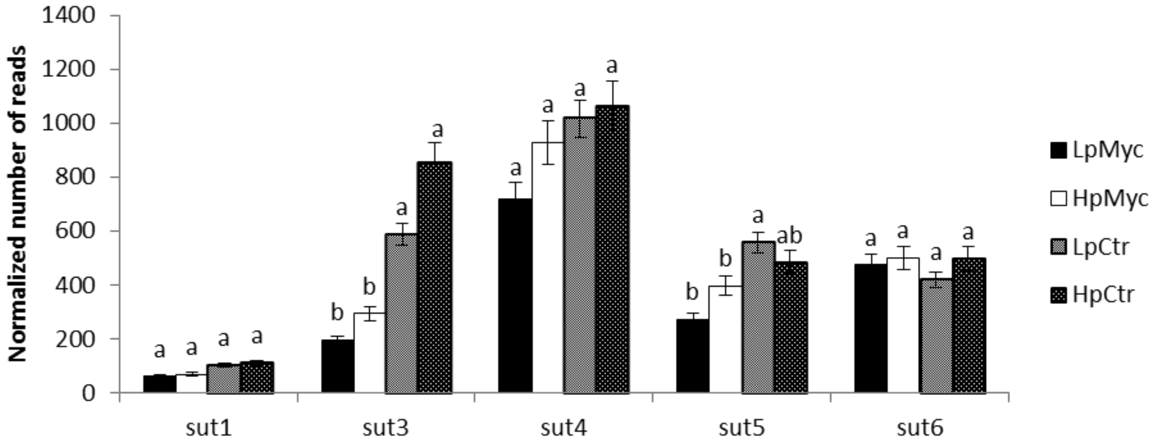

Figure S9: **Quantification by mRNA – Sequencing (RNAseq) of the transcript abundances of sugar transporters (SUT) in the roots of *P. trichocarpa*.** Transcript abundances of six SUT genes in *P. trichocarpa* were quantified in mycorrhized (+AM) and non-mycorrhized (-AM) roots under high P (hP) and low P (lP) availability. Significant differences were estimated per gene using the Wald test. Lower case letters indicate significant differences in transcript abundances (p-value < 0.05). Number of reads in the three biological replicates per condition were normalized per gene using DESeq2.

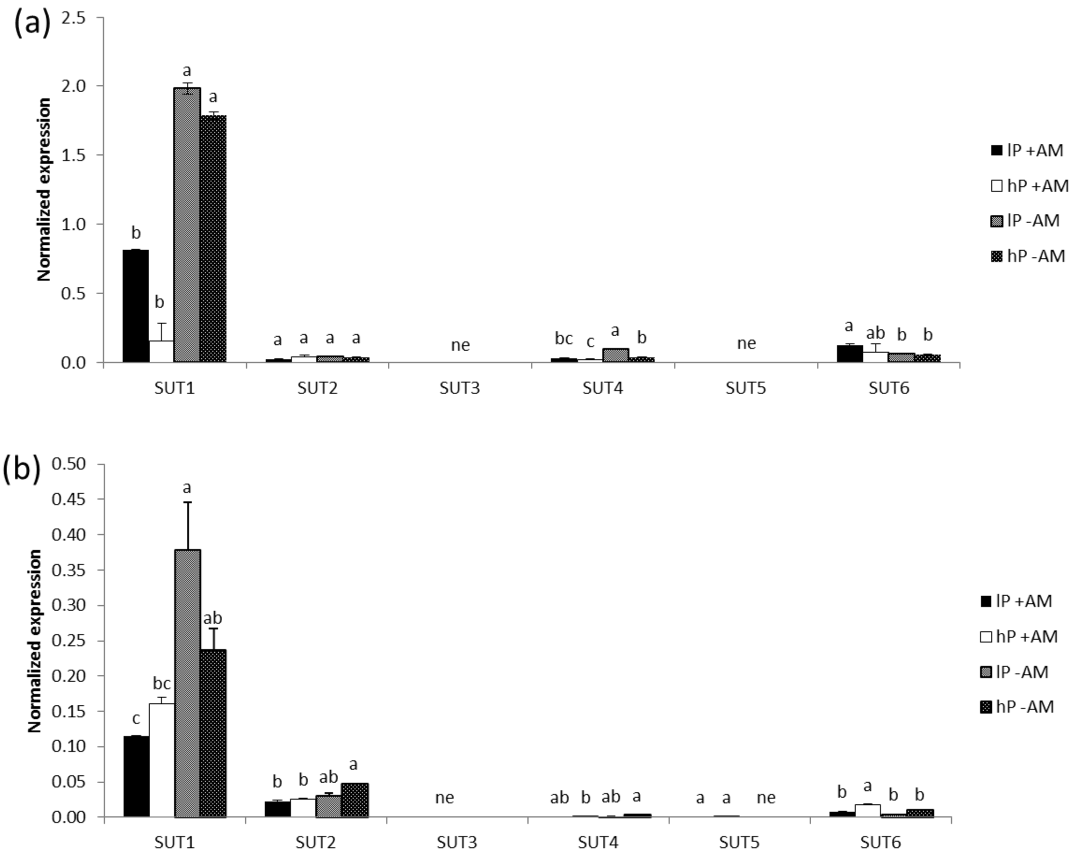

Figure S10 Quantification by qRT-PCR of the transcript abundances of 6 sugar transporters (SUT) in the shoots(a) and roots (b) of mycorrhized (+AM) and non-mycorrhized (-AM) *S. bicolor* under low P (lP) and high P (hP) condition. Values are the means of three biological replicates and three technical replicates, each. Error bars represent the SE. Ubiquitin was used as reference transcript. Statistical analysis was performed by analysis of variance (ANOVA) per gene, followed by Tuckey honest significant difference test (Tuckey HSD; $p<0.05$).
